# Amphiphilic Cell-Penetrating Peptides Containing Natural and Unnatural Amino Acids as Drug Delivery Agents

**DOI:** 10.3390/cells11071156

**Published:** 2022-03-29

**Authors:** David Salehi, Saghar Mozaffari, Khalid Zoghebi, Sandeep Lohan, Dindyal Mandal, Rakesh K. Tiwari, Keykavous Parang

**Affiliations:** 1 Center for Targeted Drug Delivery, Department of Biomedical and Pharmaceutical Sciences, Harry and Diane Rinker Health Science Campus, Chapman University School of Pharmacy, Irvine, CA 92618, USA; dsalehi@chapman.edu (D.S.); mozaf100@mail.chapman.edu (S.M.); zoghebi@chapman.edu (K.Z.); lohan@chapman.edu (S.L.); mandal@chapman.edu (D.M.); 2 Department of Pharmaceutical Chemistry, College of Pharmacy, Jazan University, Jazan 82826, Saudi Arabia; 3 School of Biotechnology, KIIT Deemed to be University, Bhubaneswar 751024, India

**Keywords:** cell-penetrating peptide, cellular uptake, cyclic peptide, diphenylalanine, drug delivery system, siRNA

## Abstract

A series of cyclic peptides, [(DipR)(WR)_4_], [(DipR)_2_(WR)_3_], [(DipR)_3_(WR)_2_], [(DipR)_4_(WR)], and [DipR]_5_, and their linear counterparts containing arginine (R) as positively charged residues and tryptophan (W) or diphenylalanine (Dip) as hydrophobic residues, were synthesized and evaluated for their molecular transporter efficiency. The in vitro cytotoxicity of the synthesized peptides was determined in human epithelial ovary adenocarcinoma cells (SK-OV-3), human lymphoblast peripheral blood cells (CCRF-CEM), human embryonic epithelial kidney healthy cells (HEK-293), human epithelial mammary gland adenocarcinoma cells (MDA-MB-468), pig epithelial kidney normal cells (LLC-PK1), and human epithelial fibroblast uterine sarcoma cells (MES-SA). A concentration of 5–10 µM and 3 h incubation were selected in uptake studies. The cellular uptake of a fluorescent-labeled phosphopeptide, stavudine, lamivudine, emtricitabine, and siRNA was determined in the presence of peptides via flow cytometry. Among the peptides, [DipR]_5_ (10 µM) was found to be the most efficient transporter and significantly improved the uptake of F’-GpYEEI, i.e., by approximately 130-fold after 3 h incubation in CCRF-CEM cells. Confocal microscopy further confirmed the improved delivery of fluorescent-labeled [DipR]_5_ (F’-[K(DipR)_5_]) alone and F’-GpYEEI in the presence of [DipR]_5_ in MDA-MB-231 cells. The uptake of fluorescent-labeled siRNA (F’-siRNA) in the presence of [DipR]_5_ with N/P ratios of 10 and 20 was found to be 30- and 50-fold higher, respectively, compared with the cells exposed to F’-siRNA alone. The presence of endocytosis inhibitors, i.e., nystatin, chlorpromazine, chloroquine, and methyl β-cyclodextrin, did not completely inhibit the cellular uptake of F’-[K(DipR)_5_] alone or F’-GpYEEI in the presence of [DipR]_5_, suggesting that a combination of mechanisms contributes to uptake. Circular dichroism was utilized to determine the secondary structure, while transmission electron microscopy was used to evaluate the particle sizes and morphology of the peptides. The data suggest the remarkable membrane transporter property of [DipR]_5_ for improving the delivery of various small molecules and cell-impermeable negatively charged molecules (e.g., siRNA and phosphopeptide).

## 1. Introduction

Cell-penetrating peptides (CPPs) are versatile systems for drug delivery due to their ability to carry a wide range of molecules. CPPs are short peptides that facilitate the cellular uptake of molecules ranging from small molecules to large fragments of DNA [1,2,3,4]. They can be used in the context of direct peptide conjugation to the cargo through covalent bonds or the incorporation into a multi-component delivery system via intermolecular hydrogen bonding, electrostatic, or hydrophobic interactions. Linear CPPs such as Tat (trans-activator of transcription) [5,6], penetratin [7,8], and oligoarginine [9] have been reported to efficiently enhance the cellular uptake of cargo molecules through different mechanisms [10].

The truncated version of Tat has been used in a number of clinical trials; however, to date, no approved therapy has used Tat [11]. One of the major challenges of Tat is induced cerebrovascular toxicity and pro-inflammatory responses [12]. It has been reported that excess hydrophobicity and/or positive charges such as polyarginine amino acids can lead to membrane lysis at high doses of peptides [13]. To reduce the cytotoxicity, an optimal number of arginine residues was reported to be approximately eight for an efficient translocation [14]. Previous studies of CPPs by us and others indicated that an optimal balance of positive charges and hydrophobicity is required for their interactions with the cell membrane and the deep penetration into the lipid bilayer to increase the cellular uptake safely and efficiently [15,16,17,18,19,20,21,22,23]. An optimal CPP can be used to improve the uptake of many drugs with limited cell permeability, such as siRNA, highly hydrophobic compounds with a limited solubility such as curcumin, and degradable drugs by proteases such as biologic drugs [24]. 

A new class of cyclic CPPs containing alternating W and R residues ([WR]_n_; n = 4, 5) was previously reported, demonstrating diverse applications in the noncovalent and covalent drug delivery [25,26], improving the cellular uptake of phosphopeptides [27] and oligonucleotides [28], generating peptide-capped gold nanoparticles (AuNPs) [29], silver and selenium nanoparticles [30], and acting as surfactants and protein stabilizers [31]. These peptides were used in conjugation with anticancer drugs such as doxorubicin [25,32,33,34], camptothecin [35], and curcumin [36]. These studies concluded that large cyclic peptides containing W and R residues [26,37] could be used as a molecular transporter of compounds.

**Figure 1:**
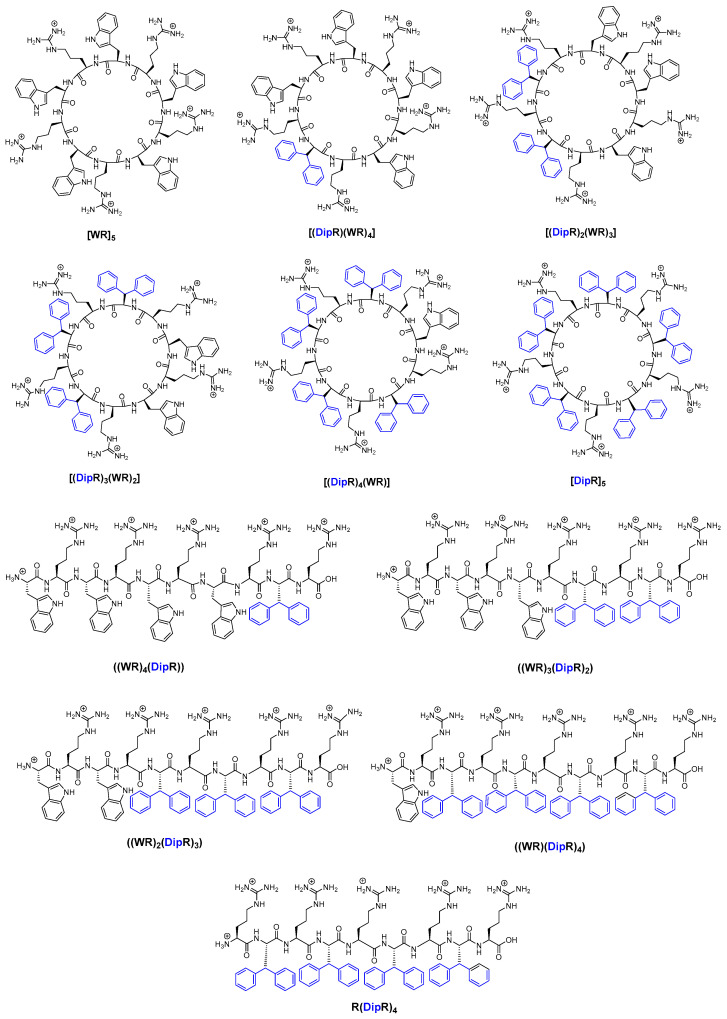
Chemical structures of synthesized cyclic and linear peptides containing R, Dip and/or W residues.

In continuation of our efforts to design cyclic CPPs with improved molecular transporter properties, we report here the design and synthesis of a new class of CPPs, containing R, diphenylalanine (Dip), and W residues (Figure 1), the evaluation of their cytotoxicity, cellular uptake, nanostructure formation, and mechanism of actions. W and Dip have Log P (partition coefficient) values of 1.82 and 2.93, respectively [38], indicating that Dip is more hydrophobic than W. A number of W residues in [WR]_5_ [26,39] was replaced with Dip residues to determine the effect of Dip on the cell penetration and the molecular transporter capability of the peptides. The hypothesis was that by replacing W residues in [WR]_5_ with more hydrophobic Dip residues, peptides with more efficient cell-penetrating and molecular transporter properties could be generated. Thus, [(DipR)(WR)_4_], [(DipR)_2_(WR)_3_], [(DipR)_3_(WR)_2_], [(DipR)_4_(WR)], and [DipR]_5_ peptides were synthesized along with five linear counterparts (Figure 1). The numbers of Dip and W residues were varied to determine the optimal balance of hydrophobicity and hydrophilic residues for the maximum cellular uptake. Previously, dipeptides with two phenylalanine amino acids have been reported as an efficient siRNA delivery system with promising prospects for cancer therapy [40,41,42,43,44,45,46,47,48]. To the best of our knowledge, this is the first report of systematic design and evaluation of CPPs containing R and Dip with or without W residues as molecular transporters. The studies allowed us to determine whether there was a change in the molecular transporter efficiency by replacing different numbers of W with Dip residues.

## 2. Materials and Methods

### 2.1. General

All resins and protected amino acids were purchased from AAPPTEC (Louisville, KY, USA). Fluorescent-labeled antihuman immunodeficiency virus (HIV) drugs [2′,3′-didehydro-2′,3′-dideoxythymidine (stavudine, d4T), 2′,3′-dideoxy-5-fluoro-3′-thiacytidine (emtricitabine, FTC), fluorescent-labeled lamivudine (3TC)], and fluorescent-labeled phosphopeptide (F′-GpYEEI) were prepared according to the previously reported procedures [37,49,50,51,52]. All cell biology reagents were purchased from Wilken Scientific (Pawtucket, RI, USA) or Fisher Scientific (Hanover Park, IL, USA). The final cyclic and linear peptides were characterized by high-resolution matrix-assisted laser desorption/ionization time-of-flight (MALDI-TOF) (Bruker, GT 0264) from Bruker Inc. (Billerica, MA, USA). α-Cyano-4-hydroxycinnamic acid was used as a matrix. 

Human epithelial ovary adenocarcinoma cells (SK-OV-3, ATCC No. HTB-77), human lymphoblast peripheral blood cells (CCRF-CEM, ATCC No. CCL-119), human embryonic epithelial kidney healthy cells (HEK-293, ATCC No. CRL-1573), human epithelial mammary gland adenocarcinoma cells (MDA-MB-468, ATCC No. HTB-132), pig epithelial kidney normal cells (LLC-PK1, ATCC No. CL-101), and human epithelial fibroblast uterine sarcoma cells (MES-SA, ATCC No. CRL-1976) were obtained from American Type Culture Collection (ATCC, Manassas, VA, USA). DAPI was used to stain the cell nuclei and was obtained from Vector Laboratories (Burlingame, CA, USA). Cell Titer 96**^®^** AQueous MTS Reagent was purchased from Promega (Madison, WI, USA). The circular dichroism analysis was performed with a J-1500 Jasco spectrophotometer using nitrogen gas and PM-539 detector. 

A peptide synthesizer (Tribute model, Protein Technologies, Inc., Tuscon, AZ, USA) was used for automated peptide synthesis. All the crude peptides were purified using reversed-phase high-performance liquid chromatography (RP-HPLC) from Shimadzu (LC-20AP) (Canby, OR, USA) by using a gradient system of water and acetonitrile (ACN) and were lyophilized. One of the analytical columns was Phenomenex 00G-4053-E0 Jupiter C18, 5 µm particle size, 250 mm × 4.6 mm, and 300 Å pore size. The second analytical column was Inertsil ODS-3 3U, Length 50 mm, and ID 4.6 mm. The preparative HPLC was LC-20AP Prominence Preparative Liquid Chromatography Shimadzu HPLC. The preparative column was 00G-4436-P0-AX Gemini Prep C18, 10 µm particle size, 250 mm × 21.2 mm, and 110 Å pore size. In analytical and preparative HPLC systems, a gradient system of solvent A (99.9% Millipore water with 0.1% TFA) and solvent B (99.9% ACN with 0.1% TFA) were used. The final peptides had a purity of approximately ≥ 95% (Supplementary Information).

### 2.2. Synthesis of Peptides

The partially protected linear peptides (NH_2_-W(Boc)-R(Pbf)-Dip-R(Pbf)-Dip-R(Pbf)-Dip-R(Pbf)-Dip-R(Pbf)-COOH), (NH_2_-W(Boc)-R(Pbf)-W(Boc)-R(Pbf)-Dip-R(Pbf)-Dip-R(Pbf)-Dip-R(Pbf)-COOH), (NH_2_-W(Boc)-R(Pbf)-W(Boc)-R(Pbf)-W(Boc)-R(Pbf)-Dip-R(Pbf)-Dip-R(Pbf)-COOH), (NH_2_- W(Boc)-R(Pbf)-W(Boc)-R(Pbf)-W(Boc)-R(Pbf)-W(Boc)-R(Pbf)-Dip-R(Pbf)-COOH), and (NH_2_-R(Pbf)-Dip-R(Pbf)-Dip-R(Pbf)-Dip-R(Pbf)-Dip-R(Pbf)-COOH) were synthesized by using NH_2_-Arg(Pbf)-trityl resin (0.572 mmol/g in 0.30 mmol scale) with an automated peptide synthesizer or the manual synthesis. The side-chain-protected linear peptides were synthesized by both automated and manual synthesis, as mentioned in the manuscript. The cyclic peptides were all synthesized in the solution phase from side-chain protected peptides after cleavage from the resin.

The resin was swelled in *N*,*N*-dimethylformamide (DMF) for 30 min. The solvent was filtered. Three equivalents of the Fmoc-protected amino acids, Fmoc-3,3-diphenyl-L-alanine, Fmoc-L-Arg(Pbf)-OH, and Fmoc-Trp(Boc)-OH were used as the building blocks. The coupling reactions were conducted in the presence of 2-(1H-benzotriazol-1-yl)-1,1,3,3-tetramethyluronium hexafluorophosphate (HBTU, 3 equiv.), *N*,*N*-diisopropylethylamine (DIPEA, 6 equiv.), and DMF for 1 h at each step. The solvent was filtered, and the resin was washed with DMF and dichloromethane (DCM) for about 5 min twice. Piperidine in DMF (20% *v*/*v*, 15 mL, 2 × 15 min) was used for the Fmoc deprotection of the assembled amino acids in each step. The solvent was filtered off, and the resin was washed, as described above. The Kaiser test was utilized to ensure that each coupling reaction was completed. The next amino acid was coupled with the same method. 

For the synthesis of linear peptides, trifluoroacetic acid (TFA), anisole, thioanisole, and dithiothreitol (DTT, 12,600 μL:700 μL:420 μL: 514 mg) were used to cleave the peptides from the resin and to remove all the protecting groups. Cold diethyl ether (10 times the amount of TFA) was added to peptides to precipitate followed by centrifuging as described previously [53], and the crude peptides were purified by using reverse-phase HPLC. Very hydrophobic peptides may be dissolved in diethyl ether and may not get precipitated completely. In those cases, the HPLC purification was conducted after evaporation of ether. MALDI-TOF was used to confirm the molecular mass versus charge (*m*/*z*) the peptides.

Linear (W-R-Dip-R-Dip-R-Dip-R-Dip-R): MALDI-TOF (*m*/*z*) C_101_H_129_N_29_O_11_ Calculated: 1878.3113, Found: 1879.3390 [M + H]^+^; Linear (W-R-W-R-Dip-R-Dip-R-Dip-R): MALDI-TOF (*m*/*z*) C_97_H_126_N_27_O_11_ Calculated: 1841.2503, Found: 1842.7980 [M + H]^+^; Linear (W-R-W-R-W-R-Dip-R-Dip-R): MALDI-TOF (*m*/*z*) C_93_H_123_N_28_O_11_ Calculated: 1804.1893, Found: 1805.7200 [M + H]^+^; Linear (W-R-W-R-W-R-W-R-Dip-R): MALDI-TOF (*m*/*z*) C_89_H_120_N_29_O_11_ Calculated: 1767.1283, Found: 1768.5530 [M + H]^+^; Linear (R-Dip-R-Dip-R-Dip-R-Dip-R): MALDI-TOF (*m*/*z*) C_90_H_119_N_24_O_10_ Calculated: 1692.0973, Found: 1693.8660 [M + H]^+^ (Figures S1–S5, Supplementary Materials).

For the synthesis of cyclic peptides, protected assembled linear peptides were first cleaved from the resin in the presence of DCM/trifluoroethanol (TFE)/acetic acid (AcOH) (35 mL:10 mL:5 mL) while agitating for 3 h. The solvent was evaporated using a low-pressure setting after mixing with hexane and DCM to remove AcOH by forming an azeotropic mixture. After adding 15 mL of DCM, enough hexane was added to make the solution cloudy. Once the cloudy solution was formed, it was agitated to avoid any precipitation. Next, the solvent was evaporated in a water bath with a temperature of 60 °C. The steps were repeated to get a homogeneous white powder with no clumps. MALDI-TOF was used to confirm the molecular mass versus charge (*m*/*z*) of linear peptides. 1–2 μL of each dissolved peptide with 50% ACN in water was applied on a steel plate (MTP 384 target plate ground steel BC, Bruker Daltonik, Bremen, Germany). Samples were dried by placing them in an air current after mixing with 1 μL of the matrix solution (20 mg/mL of α-cyano-4-hydroxycinnamic acid). 

The side-chain protected linear peptides (0.3 mmol) were cyclized in the presence of DMF (100 mL), DCM (25 mL), *N*,*N*-diisopropylcarbodiimide (DIC, 1.8 mmol, 279 μL), and 1-hydroxy-7-azabenzotriazole (HOAt, 0.9 mmol, 122.5 mg) overnight. The solvents were removed under low pressure. All the protecting groups were detached by mixing the reaction mixture containing protected peptides with TFA, anisole, thioanisole, and DTT (12,600 μL:700 μL:420 μL: 514 mg) for 3 h. The crude products were precipitated and purified as described above.

Cyclic [Dip-R-W-R-Dip-R-Dip-R-Dip-R]: MALDI-TOF (*m*/*z*) C_101_H_127_N_26_O_10_ Calculated: 1860.2963, Found: 1861.0360 [M + H]^+^; Cyclic [Dip-R-W-R-W-R-Dip-R-Dip-R]: MALDI-TOF (*m*/*z*) C_97_H_124_N_27_O_10_ Calculated: 1823.2353, Found: 1824.0460 [M + H]^+^; Cyclic [Dip-R-W-R-W-R-W-R-Dip-R]: MALDI-TOF (*m*/*z*) C_93_H_121_N_28_O_10_ Calculated: 1786.1743, Found: 1787.4050 [M + H]^+^; Cyclic [Dip-R-W-R-W-R-W-R-W-R]: MALDI-TOF (*m*/*z*) C_89_H_118_N_29_O_10_ Calculated: 1749.1133, Found: 1750.6030 [M + H]^+^; Cyclic [Dip-R-Dip-R-Dip-R-Dip-R-Dip-R]: MALDI-TOF (*m*/*z*) C_105_H_130_N_25_O_10_ Calculated: 1897.3573, Found: 1898.8040 [M + H]^+^ (Figures S6–S10, Supplementary Materials).

To synthesize fluorescent-labeled [KDipR]_5_, F’-[K(DipR)_5_] (Figure S11, Supplementary Materials), the sequence of the linear peptide was assembled on the resin using Fmoc solid-phase methodology in the same manner as described above. An extra amino acid Fmoc-L-Lys(Dde)-OH was coupled in the presence of HBTU (3 equiv.) and DIPEA (6 equiv.) in DMF (10 mL). The last Fmoc was not deprotected, and 5(6)-carboxyfluorescein was attached in the presence of HBTU (3 equiv.) and DIPEA (6 equiv.) in DMF (10 mL) after removing Dde protecting group with hydrazine monohydrate (2% *v*/*v*) solution in DMF (3 × 25 mL, each time 5 min) on the resin. As described before, piperidine in DMF (20% *v*/*v*, 15 mL, 2 × 15 min) was used for the last Fmoc deprotection of the assembled amino acid. The partially protected linear peptide was cleaved from the resin in the presence of DCM/TFE/AcOH (35 mL:10 mL:5 mL) while agitating for 3 h. The side-chain protected peptide (0.3 mmol) was cyclized in the presence of DMF (100 mL), DCM (25 mL), DIC (1.8 mmol, 279 μL), and HOAt (0.9 mmol, 122.5 mg) overnight. The solvents were removed under a low-pressure setting. All the protecting groups were detached by mixing the reaction mixture containing protected peptides with TFA, anisole, thioanisole, and DTT (12,600 μL:700 μL:420 μL: 514 mg) for 3 h. The crude product was precipitated and purified. Q-TOF mass spectrometry was used to confirm the chemical structure of F’-[K(DipR)_5_]. 

Cyclic Fluorescent-labeled [K-Dip-R-Dip-R-Dip-R-Dip-R-Dip-R]: Q-TOF (*m*/*z*) C_132_H_152_N_27_O_17_ Calculated: 2383.8373 Found: 2386.1892 [M + 3H]^+^. 

For HPLC purification, the crude peptides were dissolved in 50% of ACN in water and passed through a filter or centrifuged to make sure the injected liquid was clear for injection. The gradient elution was carried out using the mobile phase composition ranging from 95:5 water/ACN (*v*/*v*) to 5:95 water/ACN (*v*/*v*). The diode array detected the absorbance of the amide bonds and aromatic rings at the given wavelength of 220 nm and 280 nm. The HPLC solvents were delivered by pumps at a variable flow rate of 5 to 12 mL/min. Representative analytical HPLC of compounds is shown in Figure S12. The mass spectra determined the molecular mass versus charge (*m*/*z*) by using a positive reflective mode of the MALDI mass spectrometer. The instrument MALDI-TOF Autoflex Speed (Bruker Daltonik, Bremen, Germany) produced the final spectra. 

### 2.3. In Vitro Cytotoxicity Assays

SK-OV-3, CCRF-CEM, HEK-293, MDA-MB-468, LLC-PK1, and MES-SA were used to determine the cytotoxicity of the synthesized linear and cyclic peptides. About 5000, 10,000, and 50,000 cells were seeded for SK-OV-3, HEK-293, and CCRF-CEM cells, respectively, in 0.1 mL of medium per well of 96-well plates. An appropriate medium was used for each cell line (SK-OV-3: McCoy’s 5A with L-glutamine containing fetal bovine serum (FBS) (10%) and penicillin or streptomycin (1%); CCRF-CEM: RPMI-1640 medium with L-glutamine and sodium bicarbonate containing FBS (10%) and penicillin or streptomycin (1%); MDA-MB-468 cells: DMEM/F12 (1:1) (1×) with L-glutamine and 15 mM HEPES containing FBS (10%) and penicillin or streptomycin (1%); LLC-PK1 cells: Dulbecco’s Modified Eagle’s Medium with 4500 mg/L glucose, L-glutamine and sodium bicarbonate, without sodium pyruvate liquid containing FBS (10%) and penicillin or streptomycin (1%); MES-SA cells: consisting of half SK-OV-3 and half CCRF-CEM media; and HEK-293 cells: Minimum Essential Medium Eagle with Earle’s salts and sodium bicarbonate, without L-glutamine containing FBS (10%) and penicillin or streptomycin (1%)). Then, 20 µL of peptides containing 5% DMSO in water were added to each well. 

Adhesive cells were detached with 0.05% trypsin/EDTA after 15 min. The suspended cells were seeded in the medium 24 h prior to the experiment according to the previously reported procedure [26]. The medium was changed before adding the peptides for adherent cells. Each concentration 5, 10, 25, 50, and 166 µM (containing 0.8% DMSO) had triplicate wells to minimize possible errors. The plates were incubated for 24 h at 37 °C in a humidified atmosphere of 5% CO_2_. The cell viability was measured by utilizing Multi-Mode Microplate Reader (SpectraMax M5 model, Molecular Devices, San Jose, CA, USA) after adding 20 µL of the MTS reagent to each well and incubating for 3 additional hours. The wavelength intensity at 490 nm showed the approximate number of live cells. The protocol manager was selected “endpoint” on the spectrophotometer’s software (SoftMax Pro 7). The 96-well plates were inserted inside spectrophotometer, and the data were acquired. The percentage of cell viability was calculated using the following equation:

(1) $$=\left(\frac{\mathrm{Average}\;\mathrm{OD}\;\mathrm{values}\;\mathrm{of}\;\mathrm{samples}\;\mathrm{treated}\;\mathrm{with}\;\mathrm{the}\;\mathrm{compound}\;}{\mathrm{Average}\;\mathrm{OD}\;\mathrm{values}\;\mathrm{of}\;\mathrm{no}\;\mathrm{treatment}\;\mathrm{samples}\;}\right)*100$$

### 2.4. Cellular Uptake Studies

CCRF-CEM and SK-OV-3 cells were used to measure the cellular uptake of a fluorescent-labeled phosphopeptide (F’-GpYEEI) (2 µM), stavudine (F’-d4T) (2 µM) lamivudine (F’-3TC) (1–2 µM), emtricitabine (F’-FTC) (2 µM), and siRNA (F’-siRNA) (36 nM) with the least toxic concentration for each linear and the cyclic peptide (10 µM). The uptake was measured in the presence and absence of the peptides. The SK-OV-3 (0.5 million) and CCRF-CEM (a million) cells were incubated in each well of 6-well plates with the synthesized peptide and the fluorescent-labeled compound in one well. Another well contained the fluorescent-labeled compound alone for 3 h in an incubator (37 °C in a humidified atmosphere of 5% CO_2_). The media did not contain FBS, and the final volume of each well was 2 mL. Similar to the cytotoxicity study, adherent cells do not receive drugs right away, and a day must pass to allow cells to attach to the surface. For more details, please refer back to the cytotoxicity method described above.

The cells were transferred to FACS analysis tubes after being washed with phosphate-buffered saline (PBS). The cells were centrifuged 2500 RPM for 5 min with Fisher Scientific accuSpin Micro 17 to precipitate the cells and to allow replacing the supernatant. The cells were then vortexed with Nerl Blood Bank Saline pH 7–7.2 (flow cytometry buffer) and transferred to the tube through a sterile cell strainer 40 µm Nylon Mesh. Finally, the cells were resuspended in flow cytometry buffer and analyzed by flow cytometry (FACSVerse model, Becton Dickinson, San Jose, CA, USA) using the FITC channel and the BD FACSuite software. The device was washed before each experiment, and the flow rate was set at medium. Finally, the cells were analyzed by using FITC channel 495 nm argon laser for the excitation and the emitted 519 nm fluorescence for detection. The data presented was based on the FITC-A mean intensity signal for 10,000 cells collected. All assays were performed in triplicates. Results were evaluated to determine whether the peptides are facilitating the fluorescence compound to cross the membrane. 

### 2.5. Mechanism of Cellular Uptake of Cargo

To determine the mechanism of the cellular uptake, F’-[K(DipR)_5_] or the physical mixture of the F’-GpYEEI and [DipR]_5_ were used to measure the fluorescence in the treated CCRF-CEM cells. F’-[K(DipR)_5_] (5 or 10 µM) or the physical mixture of the F’-GpYEEI (2 µM) and [DipR]_5_ (5 or 10 µM) were incubated with CCRF-CEM cells over 3 h. After each incubation, the medium containing the peptide and cells was removed. The same protocol as the cellular uptake study was used to prepare the cells as described above. Finally, the cells were resuspended in the buffer and analyzed by flow cytometry using the FITC channel and the BD FACSuite software. The data presented was based on the percentage of FITC-A mean intensity signal for 10,000 cells collected. All assays were performed in triplicates.

A similar study was conducted in the presence of endocytosis inhibitors, such as nystatin, chlorpromazine, chloroquine, and methyl β-cyclodextrin to determine whether the uptake for F’-[K(DipR)_5_] or the fluorescent-labeled phosphopeptide in the presence of [DipR]_5_ is endocytosis-dependent. The cells were preincubated by various inhibitors including nystatin (50 μg/mL), chloroquine (100 μM), chlorpromazine (30 μM), and methyl-β-cyclodextrin (2.5 mM) for 30 min. The cells were incubated with the physical mixture of F’-GpYEEI and [DipR]_5_ (10 μM) or F’-[K(DipR)_5_] (10 μM) with an inhibitor for 3 h. The flow cytometry study was performed as described above.

### 2.6. Circular Dichroism

Samples were prepared in 100 µM concentration dissolved in water only. The cuvette with the 1 mm path length was used for all the measurements. The circular dichroism analysis was performed with a J-1500 Jasco spectrophotometer using nitrogen gas and PM-539 detector. Parameters were selected as follows: data interval 0.1 nm, data points 701, data pitch 0.1 nm, CD scale 200 mdeg/0.1 dOD, FL scale 200 mdeg/0.1 dOD, D.I.T 2 s, bandwidth 1 nm, cell length 1 mm, start mode immediately, scanning mode continuous, scanning speed 50 nm/min, shutter control auto, and N_2_ flowmeter manual. An average of three readings between 190 and 260 nm were collected for each sample. The analysis was carried out using one of Multivariate Regression Analyses, which is Principal Component Regression (PCR) with a basis set containing 26 proteins reference under the following conditions: standardization of result 100, replaced negative value to zero, and rejection percentage of 1%.

### 2.7. Transmission Electron Microscopy

Transmission electron microscopy (TEM) was done on a LVEM 5D Microscope model (Delong instruments, Brno, Czech Republic) samples were prepared on an Ultrathin Carbon Film on Lacey Carbon Support Film with 400 mesh copper from Ted Pella. Dilute solutions of 30 μM concentration were used for the TEM analysis. Samples were dropped on grids, evaporated overnight, and not stained. They were incubated for approximately 2 h after thawing from a freezer (0 to −18 °C). Samples were air-dried, and images were obtained with a LVEM5 (Delong Instruments, US) 5 keV field emission gun. The camera was Zyla 5.5 Scientific CMOS with specifications of exposure 50–200 [ms], pixel readout 200 MHz—lowest noise, dynamic range 12-bit (low noise), and summing 1.

### 2.8. Dynamic Light Scattering

The samples were prepared in 10 µM concentration and were incubated for approximately 2 h after thawing from a freezer (0 to −18 °C). Samples were analyzed by dynamic light scattering (DLS) for particle sizes and the polydispersity index (PI). The data were acquired by using a Zetasizer Nano ZS (Malvern, Worcestershire, UK), which has a 0.3 nm to 10 µm particle size range and was equipped with a laser beam (λ = 633 nm; 4 mW) and a scattered light detector positioned at an angle of 173° (noninvasive backscatter) in order to unmask scattered light signals of low intensity originated by the smaller particles. The analytical model was based on protein analysis. The sensitivity of the device is reported to be at 0.1 mg/mL (lysozyme). The refractive index and absorption were set at 1.450 and 0.001 for the material. The refractive index, viscosity, and temperature were set at 1.33, 0.8872, and 25 °C for dispersant. Particle sizes and PI measurements were made in disposable cuvettes (ZEN0040) at 25 °C and variable runs per measurement.

### 2.9. Confocal Microscopy

Coverslips were placed in a 6-well plate wells and covered with 2 mL of media. Human epithelial mammary gland/breast adenocarcinoma cells (MDA-MB-231, ATCC No. HTB-26) were used. 200,000 cells per well were seeded with DMEM media low glucose (4 mM L-glutamine, 1000 mg/L glucose, and 110 mg/L sodium pyruvate) overnight on a cover glass in 6-well plates. The cells were treated with the physical mixture of F’-GpYEEI and [DipR]_5_ (10 μM) or F’-[K(DipR)_5_] in DMEM media low glucose without FBS and incubated for 3 h at 37 °C in a humidified atmosphere of 5% CO_2_. Next, the media was removed, and cells were washed three times with 2 mL PBS in each well. Cells were fixed in 2 mL of 3.7% formaldehyde for 10 min and were washed 3 times in PBS (pH 7.6). 1 mL of the Texas red solution was pipetted (40 µL of Texas Red and 100 mg BSA in 10 mL of PBS) in each well to stay for 1 h at room temperature. The cells were washed at least 3 times with 2 mL of PBS for 5 min. The nucleic staining with 13 µL of DAPI on each slide was completed. Cells were covered with a coverslip carefully without air bubbles. The slide dried horizontally overnight in a dark place that had airflow. The cells were photographed and video recorded by using Nikon Instruments A1 Confocal Laser Microscope Series. Operating the software NIS Elements version 4.3, the videos and images were captured showing DAPI expressing as blue, Texas red expressing as red, and FITC expressing as green. Images were taken by using lasers sequentially with a 60× objective lens. A drop of oil was placed on slides. The scan mode was selected as Galvano. The scanning speed and size were selected to be 5 frames/second and 1024 resolutions. Refractive index, pinhole size, and numerical aperture were 1.51, 40.9 µm and 1.4, respectively.

A similar study was conducted in the presence of endocytosis inhibitors. The cells were preincubated by various inhibitors including nystatin (50 μg/mL), chloroquine (100 μM), chlorpromazine (30 μM), and methyl-β-cyclodextrin (2.5 mM) for 30 min. The cells were incubated with F’-[K(DipR)_5_] (10 μM) and an inhibitor for 3 h. The confocal microscopy images were taken using a widely used open-source image analysis tool, called Fiji software. It can be downloaded at https://fiji.sc/ (accessed on 4 March 2022).

### 2.10. Plasma Stability Study

The metabolic stability of [DipR]_5_ was assessed in human plasma by using a high-resolution mass spectrometer (LC-QTOF-MS). To remove the background interference, the data were processed by using a narrower (±0.1 *m*/*z*) extracted ion chromatogram (EIC) window. Test compound (500 µM) was incubated in 25% human plasma in water at 37 °C. 50 µL aliquots were taken at different time points (0, 0.5, 1, 2, 3, and 13 h), and the plasma proteins were precipitated by the addition of cold ethanol (50 µL) and incubated for 5 min in ice and water. The samples were centrifuged at 7000 RPM for 5 min, and the supernatant (10 µL) was injected into LC-QTOF-MS (Impact II model, Bruker Daltonik, Bremen, Germany). The samples were analyzed on Waters Xterra MS C18 5 µm 2.10 mm × 50 mm column at a flow rate of 0.3 mL/min using a linear gradient of aqueous ACN in the presence of formic acid (0.1%, *v*/*v*) as ion pair reagent. The analytes were quantified by their peak areas of EIC. The percentage digestion of the test compound was calculated relative to the peak areas at 0 min.

### 2.11. Data Analysis 

The data are presented as the mean standard deviation for the stated number of samples. A significant difference test was performed using One-way ANOVA (and nonparametric or mixed), assuming equal SDs, assuming Gaussian distribution of residuals, corrected for multiple comparisons using statistical hypothesis testing (Dunnett), and reported multiplicity adjusted *p*-value for each comparison. Each *p*-value is adjusted to account for multiple comparisons. The alpha threshold was set to 0.05 with 95% confidence interval.

## 3. Results and Discussion 

### 3.1. Chemistry

**Figure 2:**
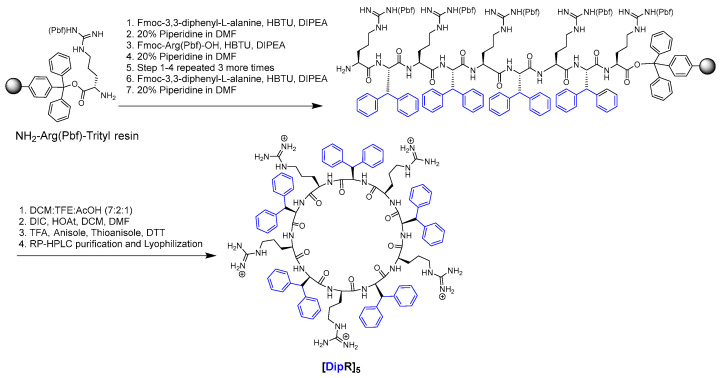
Synthesis of [DipR]_5_.

Fmoc solid-phase peptide synthesis method was used to assemble the sequences on the resin. As representative examples, the synthesis of [DipR]_5_ and fluorescent-labeled [DipR]_5_ are shown in Figure 2 and Figure S11 (Supplementary Materials), respectively. Fmoc-protected amino acids, Fmoc-3,3-diphenyl-L-alanine and Fmoc-L-Arg(Pbf)-OH were coupled to the free amino group on the NH_2_-Arg(Pbf)-trityl resin in the presence of HBTU, DIPEA, and DMF for 1 h, followed by Fmoc deprotection in the presence of piperidine. 

For the synthesis of linear peptides, all protecting groups were removed, and the peptides were cleaved from the resin in the presence of the cleavage cocktail containing TFA, anisole, thioanisole, and DTT. For the synthesis of cyclic peptides, the protected assembled peptides were cleaved from the resin first in the presence of a cleavage cocktail containing DCM/TFE/AcOH while agitating for 3 h. The side-chain-protected linear peptides were cyclized in the presence of DMF, DCM, DIC, and HOAt overnight. To complete the synthesis of cyclic peptides, all protecting groups were removed, and the peptides were cleaved from the resin in the presence of the cleavage cocktail of TFA, anisole, thioanisole, and DTT. 

The synthesis of the fluorescent-labeled [DipR]_5_ was accomplished using a similar procedure except assembling an additional amino acid residue Fmoc-Lys(Dde)-OH before cyclization. The Dde protecting group was removed in the presence of hydrazine monohydrate in DMF. After coupling reaction with 5(6)-carboxyfluorescein in the presence of HBTU and DIPEA, the Fmoc group was removed, and the cyclization was conducted as described above (Figure S11, Supplementary Materials). Cold diethyl ether was added to precipitate the crude peptide before centrifuging. All the peptides were purified using reversed-phase HPLC and characterized by high-resolution MALDI-TOF mass spectrometry (Figures S1–S10, Supplementary Materials) with about ≥95% purity.

### 3.2. Biological Activities

#### 3.2.1. In Vitro Cytotoxicity Assays

**Figure 3:**
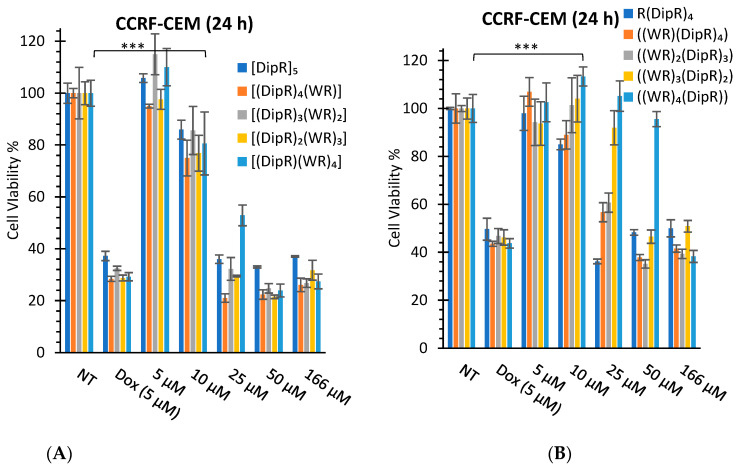

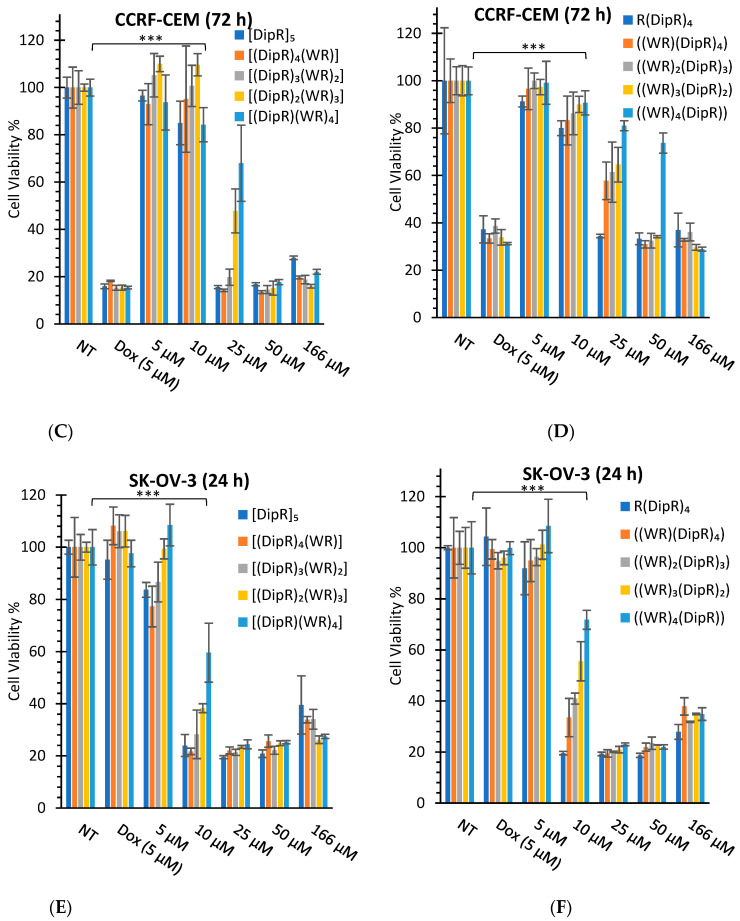

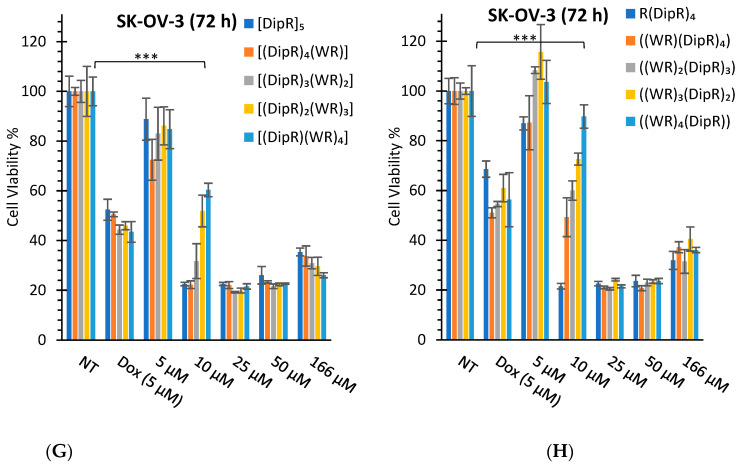
Cytotoxicity study of cyclic (**A**,**C**,**E**,**G**) and linear (**B**,**D**,**F**,**H**) peptides in CCRF-CEM cells and SK-OV-3 after 24 h and 72 h incubation. Results are mean ± SD (n = 3) (*** *p* < 0.001 treatment vs. no treatment (NT)).

**Figure 4:**
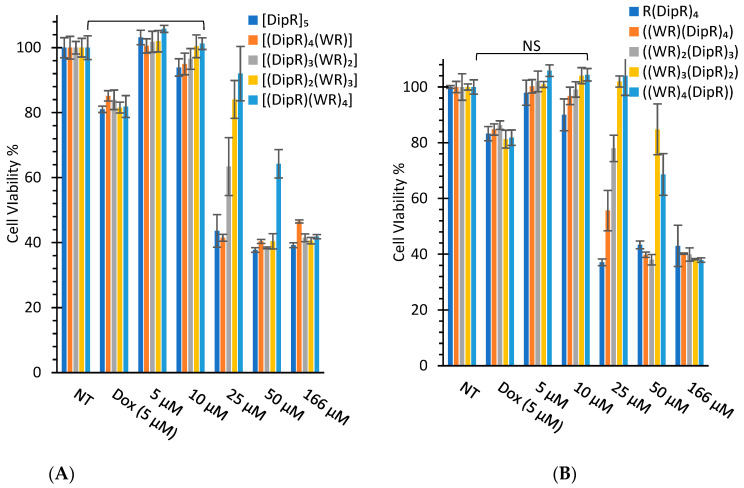
Cytotoxicity study of cyclic (**A**) and linear (**B**) peptides in HEK-293 cells after 24 h incubation (NT = No Treatment) (NS = no significant).

The synthesized peptides were evaluated for their cytotoxicity against selected cancer and normal cell lines (Figure 3 and Figure 4). Kidney cells (HEK-293 and LLC-PK1) are commonly used for the determination of cytotoxicity in normal cells to evaluate general toxicity in different organs. First, all cyclic and linear peptides were tested against SK-OV-3, CCRF-CEM, and HEK-293 cells. SK-OV-3 and CCRF-CEM represent cancer cells, while HEK-293 represents normal cells. SK-OV-3 are adherent cells, while CCRF-CEM are suspended cells. Then, the selected peptide, [DipR]_5,_ was examined further against MDA-MB-468, LLC-PK1, and MES-SA cells. MES-SA is a doxorubicin-resistant cell, while MDA-MB-468 is a triple-negative breast cancer cell line. The data provided information about the potential application of the peptides in different types of cancer cells. 

In general, the replacement of Dip with W attenuated the cytotoxicity, as shown in Figure 3 and Figure 4. Cyclic [DipR]_5_ and linear R(DipR)_4_ significantly reduced the cell proliferation of CCRF-CEM by 64% and 63%, respectively, at a concentration of 25 µM while cyclic [(DipR)(WR)_4_] decreased the cell proliferation by 47% and linear ((WR)_4_(DipR)) did not show any cytotoxicity at a concentration of 25 µM after 24 h incubation (Figure 3 ). The same pattern was observed in SK-OV-3 and HEK-293 cells. Cyclic [DipR]_5_ and linear R(DipR)_4_ significantly reduced the cell proliferation of SK-OV-3 by 81% each, at a concentration of 25 µM while cyclic [(DipR)(WR)_4_] and linear ((WR)_4_(DipR)) decreased the cell proliferation by 76% and 77%, respectively, after 24 h incubation (Figure 3). Cyclic [DipR]_5_ and linear R(DipR)_4_ significantly reduced the cell proliferation of HEK-293 by 56% and 63%, respectively, at a concentration of 25 µM. In comparison, cyclic [(DipR)(WR)_4_] and linear ((WR)_4_(DipR)) decreased the cell proliferation by 8% and 7%, respectively, after 24 h incubation (Figure 4).

The presence of a higher number of Dip residues in the peptide increased the cytotoxicity in CCRF-CEM cells. Cyclic [DipR]_5_ and linear R(DipR)_4_ significantly reduced the cell proliferation of CCRF-CEM by 84% and 66%, respectively, at a concentration of 25 µM while cyclic [(DipR)(WR)_4_] and linear ((WR)_4_(DipR)) decreased the cell proliferation by 32% and 19%, respectively after 72 h incubation (Figure 3). The difference of incubation between 24 h and 72 h in SK-OV-3 was not significant. Cyclic [DipR]_5_ and linear R(DipR)_4_ significantly reduced the cell proliferation of SK-OV-3 by 75% and 78%, respectively, at a concentration of 10 µM while cyclic [(DipR)(WR)_4_] and linear ((WR)_4_(DipR)) decreased the cell proliferation by 40% and 10%, respectively, after 72 h incubation (Figure 3).

In general, the compounds were found to be more cytotoxic against SK-OV-3 cells when compared with CCRF-CEM and HEK-293 cells. For example, cyclic [DipR]_5_ inhibited the cell proliferation of CCRF-CEM, SK-OV-3, and HEK-293 by 14%, 76%, and 6%, respectively, at 10 µM and after 24 h incubation (Figure 3 and Figure 4). The compounds were found to be slightly selective against ovarian cancer cells at specific concentrations and incubation time. The cytotoxicity against normal cells (HEK-293) was significantly less after 24 h incubation and specifically at 10 µM concentration.

The cytotoxicity of the cyclic peptides against CCRF-CEM and HEK-293 cells was minimal at the concentration of 10 μM after 24 h incubation. For example, [DipR]_5_ inhibited cell proliferation by 6% for HEK-293 cells and 14% for CCRF-CEM cells after 24 h incubation at 10 μM concentration. The rest of the compounds showed minimal cytotoxicity at 10 μM concentration in both cell lines.

**Figure 5:**
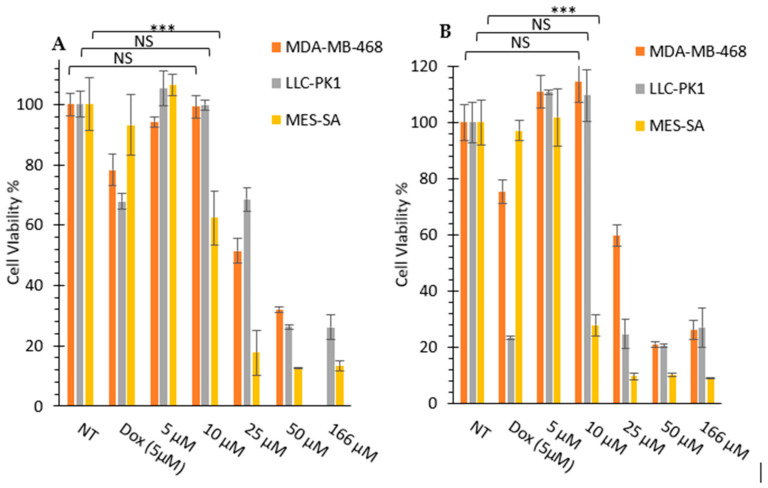
Cytotoxicity study of [DipR]_5_ in MDA-MB-468, LLC-PK1, and MES- SA cells after 24 h (**A**) and 72 h; (**B**) incubation. Results are mean ± SD (n = 3) (NS = not significant, *** *p* < 0.001 treatment vs. no treatment (NT)).

[DipR]_5_ was selected for further cytotoxicity studies in MDA-MB-468, LLC-PK1, and MES-SA cells to determine the concentration with minimal cytotoxicity for further studies. Figure 5 shows that 10 µM concentration of [DipR]_5_ reduced the cell proliferation of MES-SA cells by 38% after 24 h incubation. However, no significant cytotoxicity was observed against MDA-MB-468 and LLC-PK1 cells at 10 µM concentration after incubating 24 h and 72 h. The differential cytotoxicity behavior of [DipR]_5_ in MES-SA cell line versus MDA-MB-468 and LLC-PK1 cells could be due to many factors, such as higher uptake or less efflux in MES-SA cells. More experiments are required to explain this differential behavior. 

**Figure 6:**
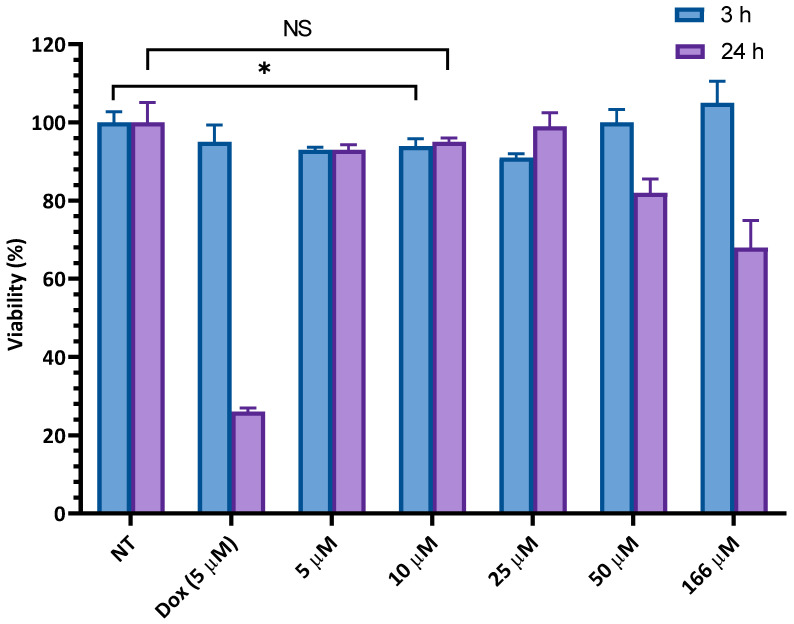
Cytotoxicity of fluorescence-labeled [DipR]_5_ in CCRF-CEM cells for 3 h and 24 h incubations. Results are mean ± SD (n = 3) (* *p* < 0.05 treatment vs. no treatment (NT), NS = not significant).

The cytotoxicity of F’-[K(DipR)_5_] was also examined in CCRF-CEM cells at 3 h and 24 h incubations (Figure 6). At the highest concentration of 166 µM, no cytotoxicity was observed after 3 h incubation. On the other hand, F’-[K(DipR)_5_] inhibited cell proliferation by 18% at 50 µM and 32% at 166 µM in CCRF-CEM cells after 24 h incubation. The data clearly indicated that the F’-[K(DipR)_5_] causes minimal cytotoxicity at 10 µM concentration after 3 h and 24 h incubations.

Based on these studies, 10 µM and 3 h incubation time were selected for further cellular uptake studies to minimize any cytotoxicity related to the compounds. Thus, the physical mixture of the least cytotoxic concentration of peptides with fluorescent-labeled cargos, F’-GpYEEI, F’-d4T, F’-3TC, F’-FTC, or F’-siRNA, and F’-[K(DipR)_5_] alone was utilized in flow cytometry by using the least cytotoxic concentration in CCRF-CEM cells for 3 h incubation.

#### 3.2.2. Cellular Uptake Studies

We have previously reported the cellular uptake of a number of fluorescent-labeled compounds in the presence of CPPs containing W and R amino acids [26,37]. In this study, the replacement of one or more W residues with Dip residues was made to determine whether there was a change in the molecular transporter efficiency of the peptide. The cellular uptake of F’-GpYEEI, F’-d4T, F’-3TC, F’-FTC, or F’-siRNA in the presence of each linear and cyclic peptide and the cellular uptake of F’-[K(DipR)_5_] alone were examined in CCRF-CEM and SK-OV-3 cells. We have previously shown that a ratio of 5:1 of the peptide to cargo is required for maximum uptake [27,28]. The lower ratio of peptide significantly reduced the molecular transporter property of the peptide, possibly due to the requirement of the efficient interaction of the peptide with the cargo.

**Figure 7:**
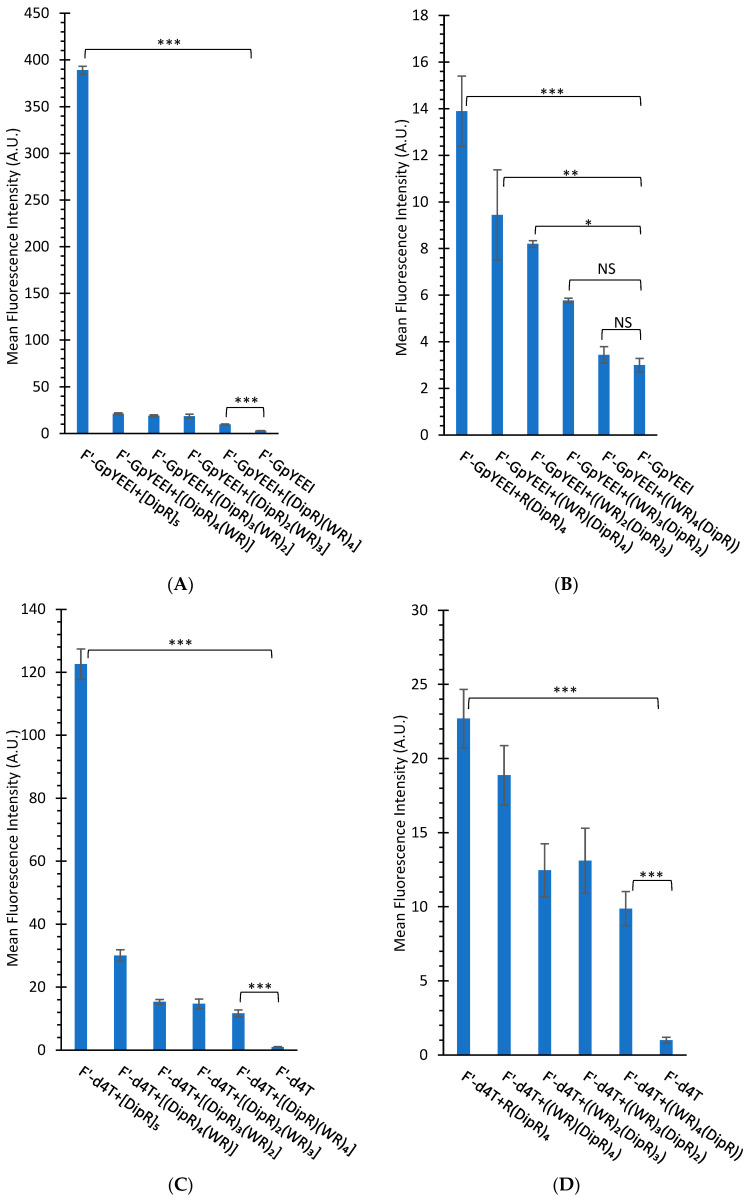

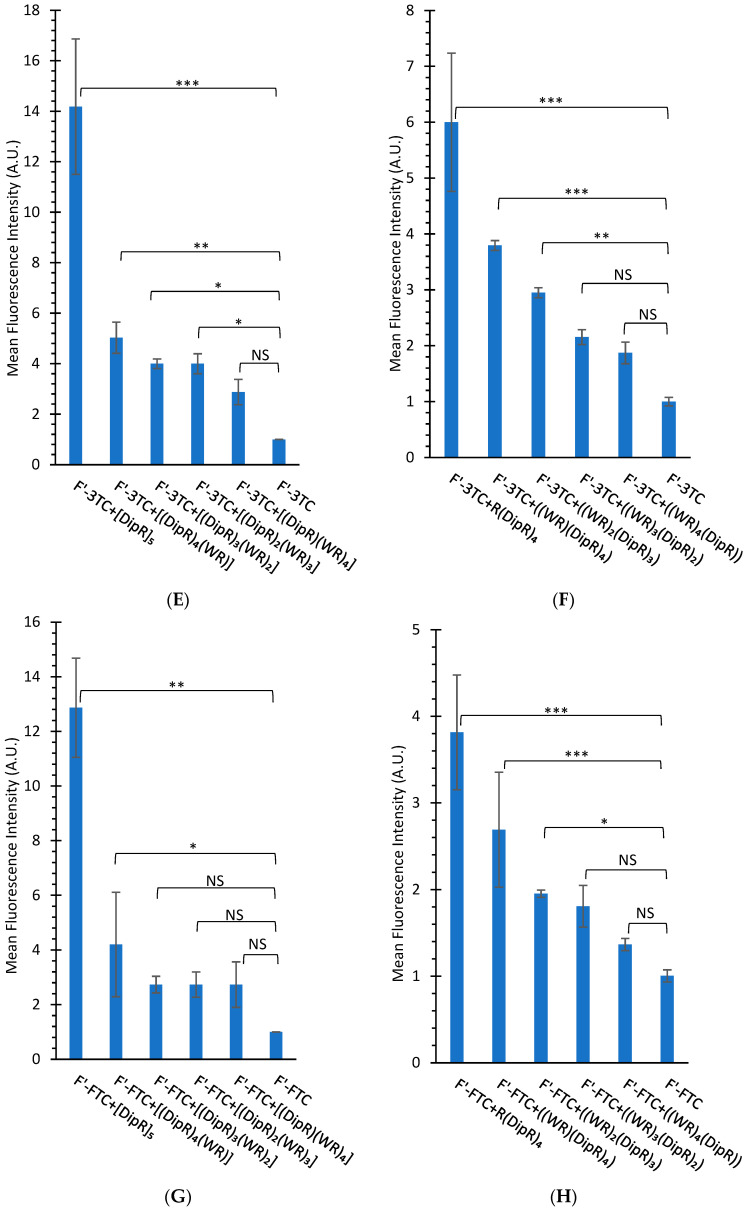
Cellular uptake of F’-GpYEEI (2 µM), F’-d4T (2 µM), F’-3TC (2 µM), and F’-FTC (2 µM) in the presence of cyclic (**A**,**C**,**E**,**G**) and linear (**B**,**D**,**F**,**H**) peptides (10 µM) for CCRF-CEM cells after 3 h incubation. Results are mean ± SD (n = 3) (* *p* < 0.05, ** *p* < 0.01, *** *p* < 0.001, NS = Not Significant).

In general, the higher number of Dip amino acids led to the higher cellular uptake in linear and cyclic peptides. For example, cyclic [DipR]_5_ and linear R(DipR)_4_ significantly increased the cellular uptake of F’-d4T (2 µM) in CCRF-CEM by 122 and 23-fold increase, respectively, at a concentration of 10 µM while cyclic [(DipR)(WR)_4_] and linear ((DipR)(WR)_4_) increased the cell uptake of F’-d4T (2 µM) in CCRF-CEM by only 12 and 10-fold increase, respectively, after 3 h incubation (Figure 7).

In general, cyclic peptides exhibited a higher uptake of the cargo molecule. For example, cyclic [DipR]_5_ and linear R(DipR)_4_ significantly increased the cellular uptake of F’-GpYEEI (2 µM) in CCRF-CEM by 130 and 5-fold increase, respectively, at a concentration of 10 µM. Cyclic [(DipR)_2_(WR)_3_] and linear ((WR)_3_(DipR)_2_) increased the cellular uptake of F’-GpYEEI (2 µM) in CCRF-CEM by approximately 7- and 1.9-fold, respectively, after 3 h incubation (Figure 7).

**Figure 8:**
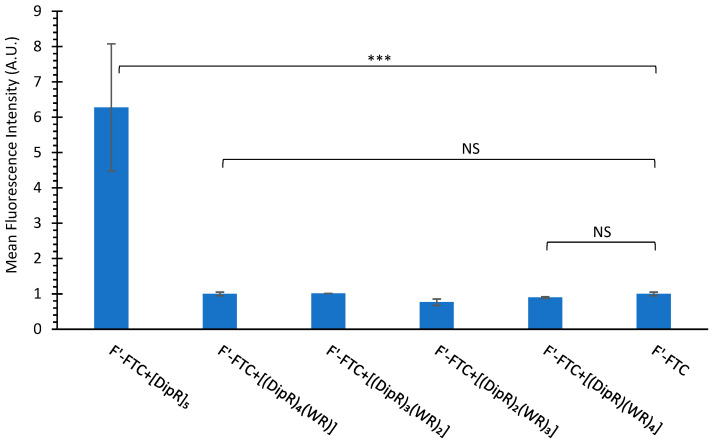
Cellular uptake of F’-3TC (2 µM) in the presence of cyclic peptides (10 µM) for SK-OV-3 cells after 3 h incubation. Results are mean ± SD (n = 3) (*** *p* < 0.001, NS = Not Significant).

The flow cytometry data for SK-OV-3 cells exhibited less uptake of fluorescence-labeled cargo in the presence of peptides when compared with CCRF-CEM cells. For example, [DipR]_5_ increased the cell uptake of F’-FTC (2 µM) in SK-OV-3 by 6-fold at a concentration of 10 µM while it increased the cellular uptake of F’-FTC (2 µM) by 13-fold in CCRF-CEM (Figure 7 and Figure 8). CCRF-CEM are suspended cells, and SK-OV-3 are adherent cells. Thus, these peptides could have fewer interactions with the adherent cells since they only interact with one side of the cells, and the other side is attached to the petri dish. SK-OV-3 cells also are known to have an efflux mechanism and to pump foreign compounds [54].

There was a difference in the cellular uptake of various fluorescent-labeled compounds. For example, cyclic [DipR]_5_ improved the cellular uptake of F’-GpYEEI, F′-3TC, F′-FTC, and F’-d4T by 130-fold, 14-fold, 13-fold, 123-fold, respectively when compared with their parent cargo alone in CCRF-CEM cells (Figure 7). The higher uptake of F’-GpYEEI was presumably due to the more efficient interactions of the positively charged [DipR]_5_ with the negatively charged phosphopeptide. There was also a difference in the cellular uptake of other anti-HIV compounds, possibly due to the differential interaction of [DipR]_5_ with these compounds.

**Figure 9:**
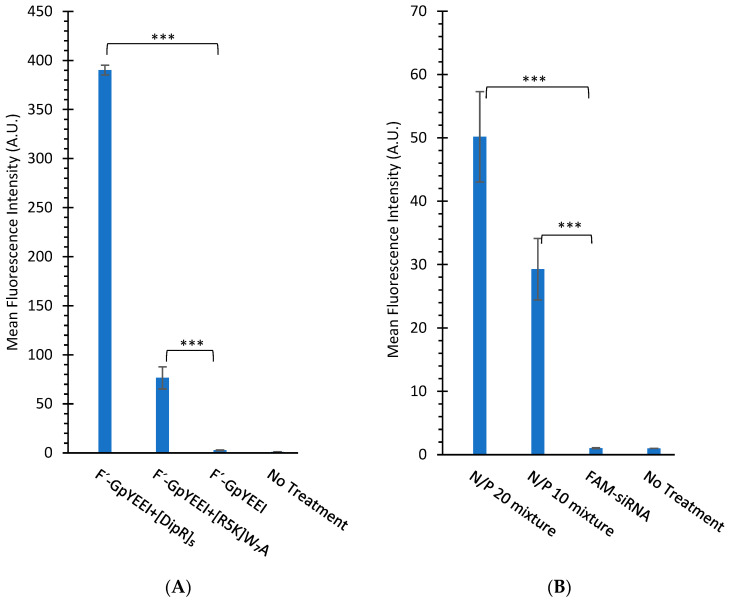
(**A**) Cellular uptake of F’-GpYEEI (2 µM) in the presence of [DipR]_5_ or [R_5_K]W₇A (10 µM) in CCRF-CEM cells after 3 h incubation. (**B**) The cellular uptake of F’-siRNA (36 nM) in the presence of [DipR]_5_ at different N/P ratios in MDA-MB-231 cells after 24 h incubation. Results are mean ± SD (n = 3) (*** *p* < 0.001).

Samara et al. reported the cellular uptake of [WR]_5_ (10 µM) in the presence of F’-GpYEEI (2 µM) increased by 4-fold [37]. We have previously reported [R_5_K]W_7_A peptide as a cargo delivery system [33] that improved the delivery of F’-GpYEEI by 26-fold after 3 h incubation. On a side-by-side comparison of [R_5_K]W_7_A and [DipR]_5_, the data showed [DipR]_5_ had 5-fold higher cellular uptake of F’-GpYEEI under the same experimental conditions after 3 h incubation in CCRF-CEM cells (Figure 9A). Furthermore, [DipR]_5_ improved the cellular uptake of F’-siRNA (36 nM) with N/P ratios of 10 and 20 by 30-fold and 50-fold, respectively when compared with siRNA alone in MDA-MB-231 cells after 24 h (Figure 9B). These data reflect that [DipR]_5_ has the potential to deliver negatively charged phosphopeptides or oligonucleotides in the cancer cells.

#### 3.2.3. Mechanism of Cellular Uptake of Cargo 

**Figure 10:**
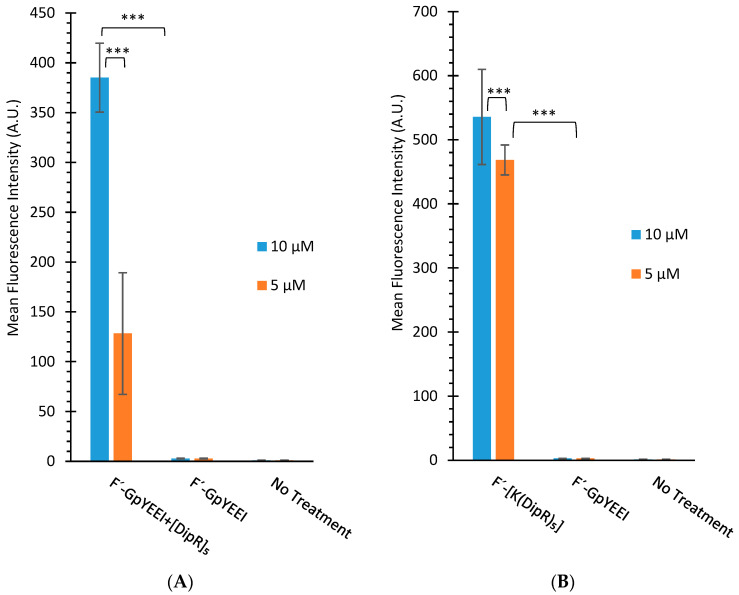
(**A**) The cellular uptake study of F’-GpYEEI (2 µM) in the presence of [DipR]_5_ (5 or 10 µM) in CCRF-CEM cells and (**B**) the cellular uptake of F’-[K(DipR)_5_] (5 or 10 µM) in CCRF-CEM cells after 3 h incubation. Results are mean ± SD (n = 3) (*** *p* < 0.001).

The uptake of F’-GpYEEI was determined in the presence of [DipR]_5_ in different concentrations. The uptake of [DipR]_5_ (10 and 5 µM) and F’-GpYEEI (2 µM) physical mixture have values of 130 and 43-fold increase for 10 and 5 µM within 3 h, respectively (Figure 10A). The data indicated a concentration-dependent uptake for F’-GpYEEI (2 µM) at two different concentrations of [DipR]_5_ (5 and 10 µM).

We also examined the cellular uptake of F’-[K(DipR)_5_] at different concentrations (10 µM and 5 µM). The uptake of F’-[K(DipR)_5_] showed slight concentration dependence uptake. The uptake of F’-[K(DipR)_5_] (10 and 5 µM) have values of 179 and 154-fold increase for 10 and 5 µM within 3 h, respectively, as compared to F’-GpYEEI (2 µM) (Figure 10B).

**Figure 11:**
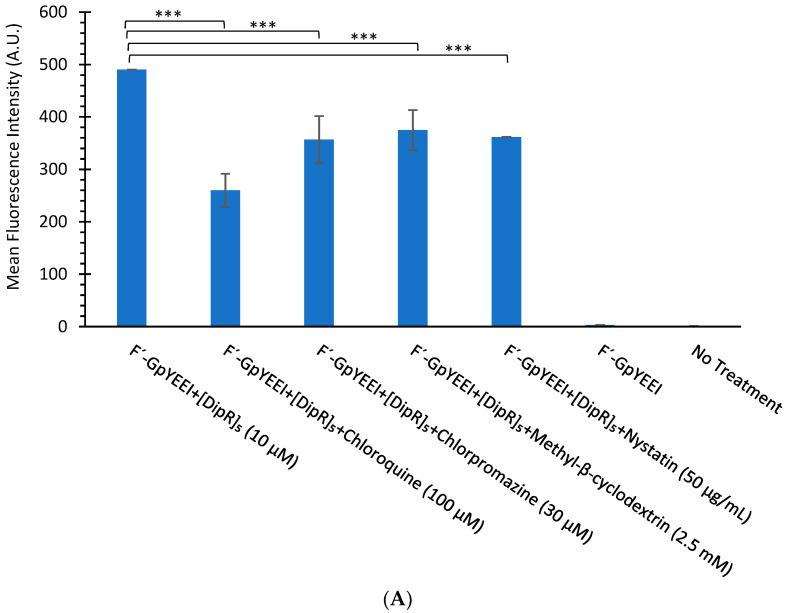

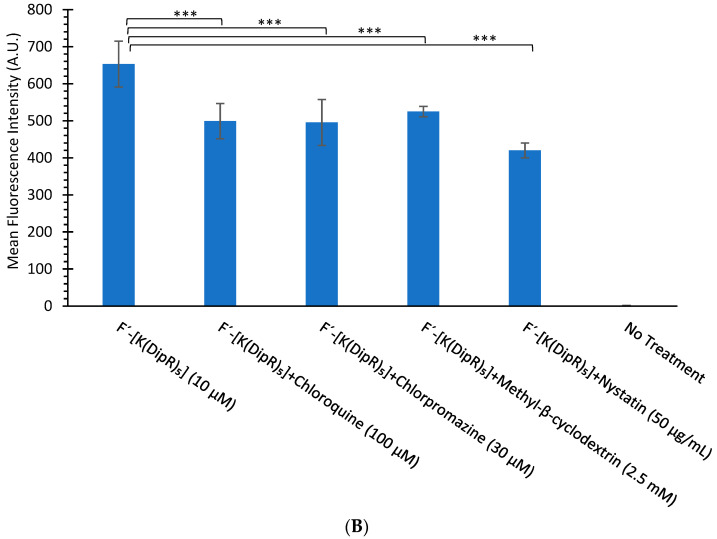
(**A**) The cellular uptake study of F’-GpYEEI (2 µM) in the presence of [DipR]_5_ (10 µM) and endocytosis inhibitors in CCRF-CEM cells after 3 h incubation. (**B**) The cellular uptake study of F’-[K(DipR)_5_] (10 µM) and endocytosis inhibitors in CCRF-CEM cells after 3 h incubation. Results are mean ± SD (n = 3) (*** *p* < 0.001).

To explore the cellular uptake mechanism of action, another study was conducted in the presence of endocytosis inhibitors such as nystatin, chlorpromazine, chloroquine, and methyl β-cyclodextrin to determine whether the cellular uptake of F’-[K(DipR)_5_] alone and F’-GpYEEI in the presence of [DipR]_5_ were endocytosis-dependent. None of the endocytosis inhibitors completely inhibited the cellular uptake. Chloroquine and nystatin reduced slightly 1.9–1.3-fold the uptake of F’-GpYEEI (2 µM) in the presence of [DipR]_5_ (10 µM) (Figure 11A). Likewise, endocytosis inhibitors chloroquine and nystatin reduced the cellular uptake of F’-[K(DipR)_5_] (10 µM) slightly by 1.3 and 1.5-fold, respectively (Figure 11B). The results suggest that a combination of multifaceted mechanisms could contribute to the uptake of F’-[K(DipR)_5_] alone and F’-GpYEEI in the presence of [DipR]_5_ across the plasma membrane. 

#### 3.2.4. Circular Dichroism

The secondary structures of all peptides were investigated with circular dichroism spectroscopy to determine whether there was any correlation between the secondary structures of the peptides and their molecular transporter properties. Figure S13 (Supplementary Materials) shows the CD spectra of peptides at 100 µM in water. In general, an α-helix structure gives rise to two negative bands at 222 and 208 nm of almost equal intensities and a strong positive band at about 193 nm. The highest percentage of α-helix structure for [(DipR)(WR)_4_] and ((WR)(DipR)_4_) with values 28.9% and 29.8%, respectively, were detected (Table S1, Supplementary Materials). The β-sheet structure exhibits a negative band at 218 nm and a positive band at 195 nm [55]. The highest values for the β-sheet structure were detected for R(DipR)_4_ and ((WR)_2_(DipR)_3_) with values 36.5% and 38.0%, respectively (Table S1, Supplementary Materials). [DipR]_5_ showed mostly a random coil formation (49.9%) with a negative band near 195 nm (Figure S13A, Supplementary Materials). [DipR]_5_ was the most effective transporter and did not exhibit any distinct structure. Thus, there was no correlation between the secondary structure of peptides and their molecular transporter properties. The cargo transporter property of [DipR]_5_ is presumably related to the nature of the interactions of this peptide with cargo molecules and the phospholipid bilayer.

#### 3.2.5. Transmission Electron Microscopy

CPPs can self-assemble and generate various types of nanoparticles (NPs) such as spherical or oval morphology [56]. Raymond et al. reported self-assembling β-sheet peptide (QW)_6_ attached to ten lysine residues (K10) to form persistent 50 nm β-sheet nanofibrils nanoparticles [57]. We examined the self-assembling tendency of a number of peptides using transmission electron microscopy (TEM) images. 

**Figure 12:**
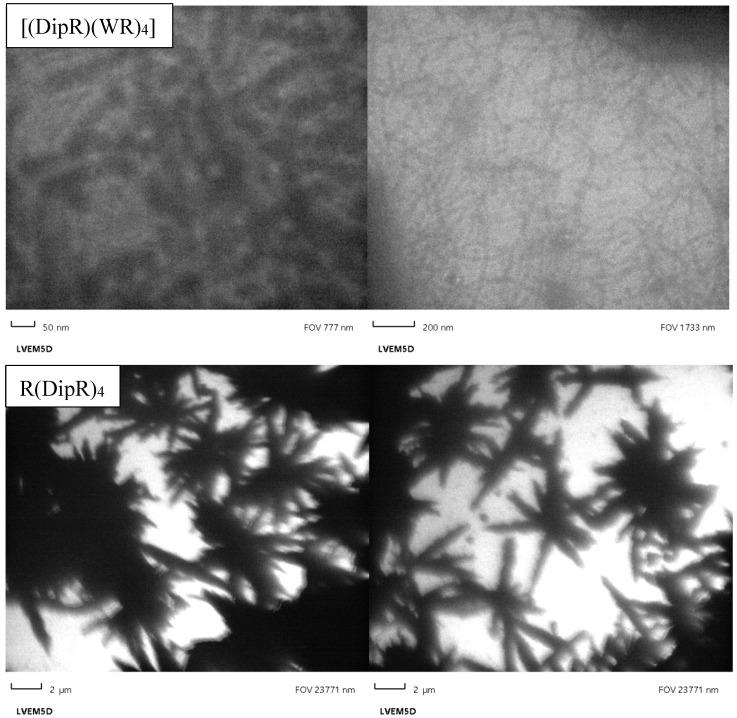

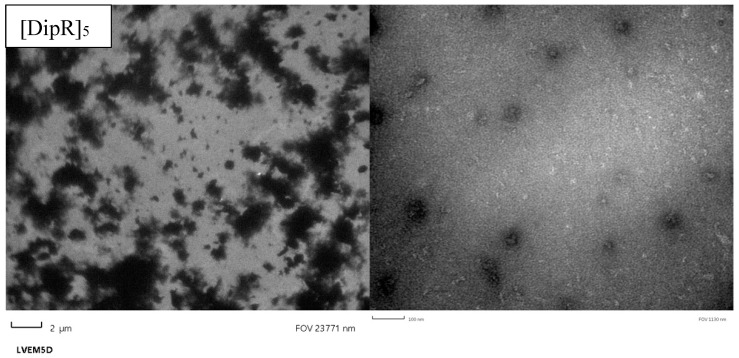
Transmission electron microscopy images of [(DipR)(WR)_4_], R(DipR)_4_, and [DipR]_5_ (different magnifications of left 2 µm and right 100 nm).

[(DipR)(WR)_4_] with the highest numbers of tryptophan residues showed a nanofiber structure with an approximate diameter of 50 nm (Figure 12). These nanofibers are composed of a distorted β-sheet and α-helix secondary structure, as observed in the CD spectrum (Table S1, Supplementary Materials). R(DipR)_4_ (Figure 12) and [(DipR)_2_(WR)_3_] (Figure S14, Supplementary Materials) showed star-shaped particles, while [DipR]_5_ showed an aggregation and nanoparticle sizes of around ≤30 nm (Figure 12). The data suggest increasing the number of diphenylalanine residues could lead to more self-assembly and aggregation. Star-like structures with an approximate size of 1000–2000 nm indicate the formation of a random network, such as nonclassic coacervates reported in the literature through the interaction between positive charges and the aromatic π-system [58].

There was no correlation between the morphology of the peptides and their cellular uptake properties. For TEM, a concentration of 30 µm was used on grids and evaporated overnight. The longer time and higher concentration allow the peptide to self-assemble and generate different structures or larger particles. For cellular uptake studies, a concentration of 10 µM and incubation time of 3 h were used with a cargo molecule. We did not study the morphology of the peptide under the same conditions. However, our earlier work has shown that large size particles are formed with longer incubation time and at a higher concentration. We expect the morphology of the peptide to be different in the presence of cargo and under the conditions used in uptake studies.

#### 3.2.6. Dynamic Light Scattering

Dynamic light scattering (DLS) was used to determine the particle size and polydispersity index (PI) for [DipR]_5_. The data confirmed the formation of nanoparticles. Cyclic [DipR]_5_ exhibited nanoparticles with Z average size of 200.6 nm and PI of 0.359 (Figure S15, Supplementary Materials). The particle formation was confirmed, as shown in TEM images for [DipR]_5_.

#### 3.2.7. Confocal Microscopy

**Figure 13:**
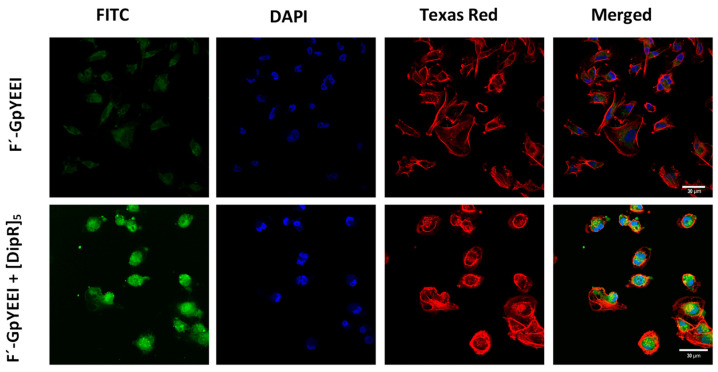
The confocal microscopy images of F’-GpYEEI (2 µM) in the presence of [DipR]_5_ (10 µM) after incubation for 3 h with MDA-MB-231 cells. Videos of the images are available in supplementary materials.

Confocal microscopy was used to determine the localization of the molecular cargo in the presence of [DipR]_5_ within the cells. MDA-MB-231 cells were seeded and treated with F’-[K(DipR)_5_] (10 μM) alone and [DipR]_5_ (10 μM) in the presence of F’-GpYEEI (2 µM) and then incubated for 3 h at 37 °C. The cellular uptake was monitored with confocal microscopy. The top panel of Figure 13 shows the control cells treated with F’-GpYEEI alone, demonstrating the very low uptake. On the lower panel of Figure 13, the confocal image of F’-GpYEEI in the presence of [DipR]_5_ is shown. A higher cellular uptake and higher intensity of the fluorescent-labeled phosphopeptide (green color) were observed in the presence of [DipR]_5_. F’-GpYEEI was mostly localized in the cytosol. In addition, a 3D video and every half micrometer image of the cells can be watched by accessing the links (Supplementary Materials) (watch the videos in 720 p quality or higher). The data suggests the efficient cytosolic delivery of F′-GpYEEI in the presence of [DipR]_5,_ and this delivery system can be beneficial for the delivery of cargos such as siRNA and phosphopeptide to the cytoplasm. 

**Figure 14:**
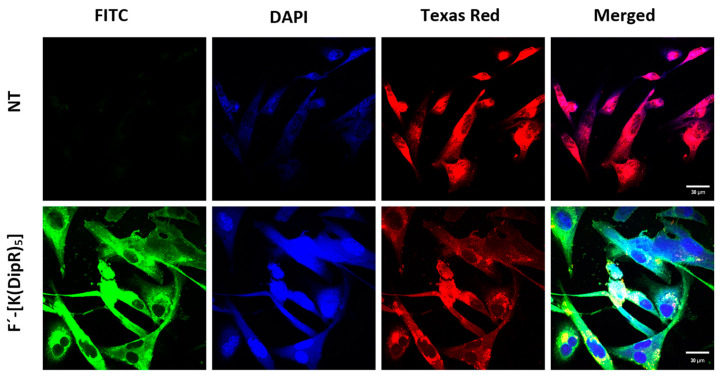
The confocal microscopy images of F’-[K(DipR)_5_] (10 µM) after incubation for 3 h with MDA-MB-231. (NT = No Treatment). Videos of the images are available in supplementary materials.

To determine the location of CPPs, the uptake of F’-[K(DipR)_5_] was monitored by confocal microscopy (Figure 14). The top panel shows the control cells without any treatments and any significant green fluorescence color. The confocal images of cells treated with F’-[K(DipR)_5_] are shown on the lower panel. A higher cellular uptake and higher intensity of F’-[K(DipR)_5_] (green color) were observed. F’-[K(DipR)_5_] was mostly localized in the cytosol. In addition, a 3D video and every half micrometer image of the cells can be watched by accessing the links (Supplementary Materials) (watch the videos in 720 p quality or higher). 

**Figure 15:**
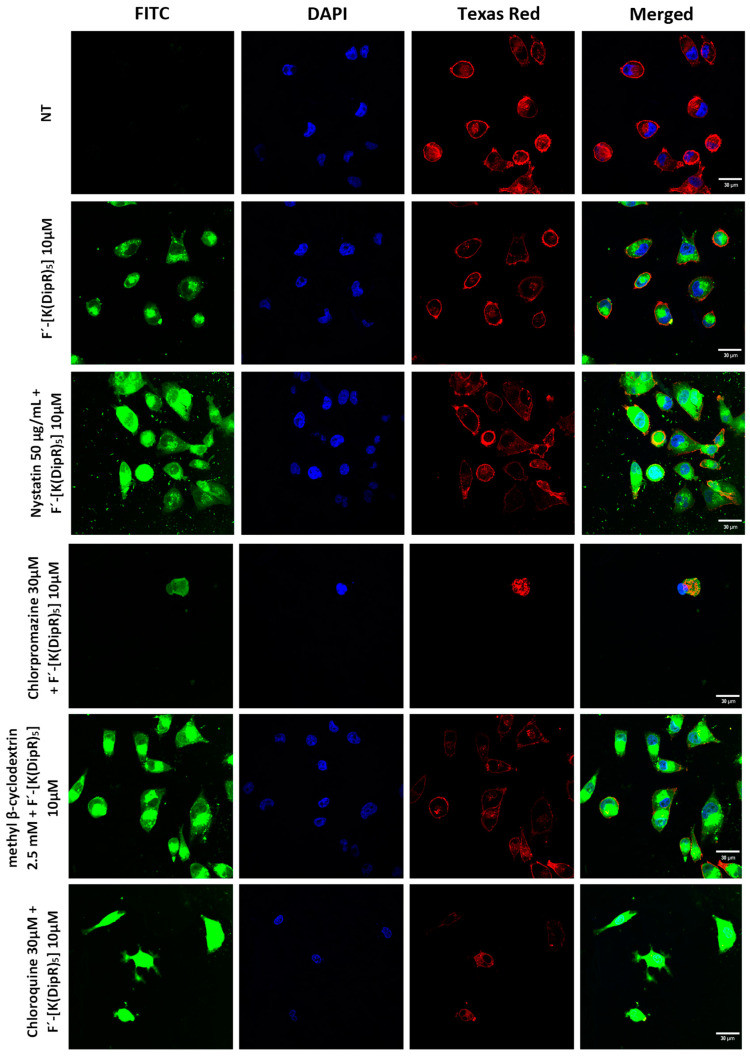
Confocal microscopy of F’-[K(DipR)_5_] (10 µM) and endocytosis inhibitors (nystatin (50 μg/mL), chloroquine (100 μM), chlorpromazine (30 μM), and methyl-β-cyclodextrin (2.5 mM)) in MDA-MB-231 cells after 3 h incubation (NT = No Treatment).

As described in flow cytometry studies above, the uptake of F’-[K(DipR)_5_] was not completely blocked by endocytosis inhibitors. The confocal microscopy studies of F’-[K(DipR)_5_] was conducted in the presence of endocytosis inhibitors, which are nystatin, methyl-β-cyclodextrin, chloroquine, and chlorpromazine to confirm the data (Figure 15). Change of concentrations did not change the intensity of the green color significantly (Figure S16, Supplementary Materials). The data are consistent with flow cytometry studies. Chlorpromazine reduced the uptake slightly. The drug is an inhibitor of clathrin-mediated endocytosis by preventing the assembly and disassembly of clathrin lattices on cell surfaces and on endosomes [59]. Nystatin and chloroquine inhibit caveolae-mediated endocytosis and clathrin-dependent endocytosis, respectively. Thus, a combination of uptake mechanisms contributes to the uptake of F’-[K(DipR)_5_] across the membrane.

#### 3.2.8. Plasma Stability of [DipR]_5_

The stability of [DipR]_5_ was determined in human plasma, and the data were analyzed using Liquid Chromatography Quadrupole Time-of-Flight mass spectrometry (LC-QTOF-MS) (Figure S17, Supplementary Materials). Data are represented in the form of percentage degradation of test compound against time by measuring area under the curve in extracted ion chromatogram (EIC) (Figure S18, Supplementary Materials). Approximately 15% degradation of [DipR]_5_ was observed after 1 h. However, degradation slowed down at the 2 h time point. The results clearly indicate the high stability of the peptide, possibly due to the presence of unnatural amino acids and the cyclic nature of the peptide.

## 4. Conclusions

[DipR]_5_ was discovered as a novel CPP and molecular transporter of small and large cargo molecules. Ten linear and cyclic peptides containing R, W, and Dip were synthesized and evaluated for their molecular transporter properties. The difference in the number of unnatural amino acids and their linear or cyclic structures provided adequate structural diversity for the study of cellular uptake. Cellular uptake studies were conducted at 10 µM and incubation for 3 h by flow cytometry and confocal microscopy techniques. Among the peptides, [DipR]_5_ significantly improved the cellular uptake of F’-GpYEEI, F’-siRNA, F’-3TC, and F’-FTC when compared with cells exposed to cargo alone. Confocal microscopy demonstrated the delivery of F’-[K(DipR)_5_] alone and F’-GpYEEI in the presence of [DipR]_5_, localizing mostly in the cytosol. Endocytosis inhibitors did not completely inhibit the uptake of these molecules, indicating a contribution of a combination of mechanisms in the uptake. Thus, [DipR]_5_ may have broad applications as an efficient CPP and a molecular transporter agent.

## Data Availability

Not applicable.

## Supplementary Materials

Supplementary data to this article can be found online via https://www.mdpi.com/article/10.3390/cells11071156/s1 and provides MALDI-TOF spectra of the peptides, analytical HPLC methods, chromatography spectra of peptides, synthetic Scheme for F’-[K(DipR)_5_], circular dichroism spectra, tabulated CD data, videos of cellular uptake, transmission electron microscopy images of [(DipR)_2_(WR)_3_], dynamic light scattering of [DipR]_5_, plasma stability of [DipR]_5_, and confocal microscopy images of F’-[K(DipR)_5_] treated with different concentrations of endocytosis inhibitors. Table S1. Estimate of peptide secondary structures; Figure S1. Linear (W-R-Dip-R-Dip-R-Dip-R-Dip-R): MALDI-TOF (*m*/*z*) C_101_H_129_N_29_O_11_ Calculated: 1878.3113 Found: 1879.3390 [M + H]^+^; Figure S2. Linear (W-R-W-R-Dip-R-Dip-R-Dip-R): MALDI-TOF (*m*/*z*) C_97_H_126_N_27_O_11_ Calculated: 1841.2503 Found: 1842.7980 [M + H]^+^; Figure S3. Linear (W-R-W-R-W-R-Dip-R-Dip-R): MALDI-TOF (*m*/*z*) C_93_H_123_N_28_O_11_ Calculated: 1804.1893 Found: 1805.7200 [M + H]^+^; Figure S4. Linear (W-R-W-R-W-R-W-R-Dip-R): MALDI-TOF (*m*/*z*) C_89_H_120_N_29_O_11_ Calculated: 1767.1283 Found: 1768.5530 [M + H]^+^; Figure S5. Linear (R-Dip-R-Dip-R-Dip-R-Dip-R): MALDI-TOF (*m*/*z*) C_90_H_119_N_24_O_10_ Calculated: 1692.0973 Found: 1693.8660 [M + H]^+^; Figure S6. Cyclic [Dip-R-W-R-Dip-R-Dip-R-Dip-R]: MALDI-TOF (*m*/*z*) C_101_H_127_N_26_O_10_ Calculated: 1860.2963 Found: 1861.0360 [M + H]^+^; Figure S7. Cyclic [Dip-R-W-R-W-R-Dip-R-Dip-R]: MALDI-TOF (*m*/*z*) C_97_H_124_N_27_O_10_ Calculated: 1823.2353 Found: 1824.0460 [M + H]^+^; Figure S8. Cyclic [Dip-R-W-R-W-R-W-R-Dip-R]: MALDI-TOF (*m*/*z*) C_93_H_121_N_28_O_10_ Calculated: 1786.1743 Found: 1787.4050 [M + H]^+^; Figure S9. Cyclic [Dip-R-W-R-W-R-W-R-W-R]: MALDI-TOF (*m*/*z*) C_89_H_118_N_29_O_10_ Calculated: 1749.1133 Found: 1750.6030 [M + H]^+^; Figure S10. Cyclic [Dip-R-Dip-R-Dip-R-Dip-R-Dip-R]: MALDI-TOF (*m*/*z*) C_105_H_130_N_25_O_10_ Calculated: 1897.3573 Found: 1898.8040 [M + H]^+^; Figure S11. Synthesis of Fluorescence-labeled [K(DipR)_5_]; Figure S12. Chromatography Spectra of peptides; Figure S13. Circular dichroism spectra of (A) [DipR]_5_; (B) [(DipR)_4_(WR)]; (C) [(DipR)_3_(WR)_2_]; (D) [(DipR)_2_(WR)_3_]; (E) [(DipR)(WR)_4_]; (F) R(DipR)_4_; (G) ((WR)(DipR)_4_); (H) ((WR)_2_(DipR)_3_); (I) ((WR)_3_(DipR)_2_); (J) ((WR)_4_DipR)) were conducted using 100 µM peptide concentration; Figure S14. Transmission electron microscopy image of [(DipR)_2_(WR)_3_]; Figure S15. Dynamic light scattering of [DipR]_5_ (10.0 µM), Z Average 200.6, PI 0.359; Figure S16. Confocal microscopy images of F’-[K(DipR)_5_] (10 and 20 µM) in the presence of chloroquine (100 and 50 μM) or chlorpromazine (30 and 15 μM), in MDA-MB-231 cells after 3 h incubation; Figure S17. The plasma stability of [DipR]_5_ chromatograms; Figure S18. The plasma stability of [DipR]_5_.
