# Supplementary material for: Amphiphilic Cell-Penetrating Peptides Containing Natural and Unnatural Amino Acids as Drug Delivery Agents
Source: Cells. 2022 Mar 29;11(7):1156. doi: 10.3390/cells11071156 (PMC8997995; doi:10.3390/cells11071156)
Supplement: Supplementary file 1 [file cells-11-01156-s001.zip › cells-1618540-supplementary.pdf]

Supplementary Materials

Amphiphilic Cell-Penetrating Peptides Containing Natural and Unnatural Amino Acids as Drug Delivery Agents

David Salehi[1], Saghar Mozaffari[1], Khalid Zoghebi[1,2], Sandeep Lohan[1], Dindyal Mandal[1,3], Rakesh K. Tiwari[1,*] and Keykavous Parang[1,*]

[1] Center for Targeted Drug Delivery, Department of Biomedical and Pharmaceutical Sciences, Chapman University School of Pharmacy, Harry and Diane Rinker Health Science Campus, Irvine, CA 92618, USA; dsalehi@chapman.edu (D.S.); mozaf100@mail.chapman.edu (S.M.); zoghebi@chapman.edu (K.Z.); lohan@chapman.edu (S.L.); mandal@chapman.edu (D.M.)

[2] Department of Pharmaceutical Chemistry, College of Pharmacy, Jazan University, Jazan 82826, Saudi Arabia

[3] School of Biotechnology, KIIT Deemed to be University, Bhubaneswar, India 751024

* Correspondence: tiwari@chapman.edu (R.K.T.); parang@chapman.edu (K.P.); Tel.: +1-714-516-5483 (R.K.T.); +1-714-516-5489 (K.P.)

Table of Contents **Page**

Linear (W-R-Dip-R-Dip-R-Dip-R-Dip-R): MALDI-TOF(m/z)...………........……..…..……… S4

Linear (W-R-W-R-Dip-R-Dip-R-Dip-R): MALDI-TOF (m/z)………….......……………….... S5

Linear (W-R-W-R-W-R-Dip-R-Dip-R): MALDI-TOF (m/z)...………..............……..……... S6

Linear (W-R-W-R-W-R-W-R-Dip-R): MALDI-TOF (m/z)……………........…….…..…….... S7

Linear (R-Dip-R-Dip-R-Dip-R-Dip-R): MALDI-TOF (m/z)....………….......………..……..... S8

Cyclic [Dip-R-W-R-Dip-R-Dip-R-Dip-R]: MALDI-TOF (m/z)...……........…………….…......S9

Cyclic [Dip-R-W-R-W-R-Dip-R-Dip-R]: MALDI-TOF (m/z)…………..............…………...S10

Cyclic [Dip-R-W-R-W-R-W-R-Dip-R]: MALDI-TOF (m/z)...…………..............…………...S11

Cyclic [Dip-R-W-R-W-R-W-R-W-R]: MALDI-TOF (m/z)……………..............…………...S12

Cyclic [Dip-R-Dip-R-Dip-R-Dip-R-Dip-R]: MALDI-TOF (m/z)....……..............…………...S13

Synthesis Scheme of F´-[K(DipR)$_5$]….………………..................…….................………...S14

Analytical HPLC Methods….……..……………………...................……..................…..S15-16

Chromatography Spectra of Peptides...………………………………............……….... .S17-26

Circular Dichroism Spectra...………………………………...……........…………...........S27

Transmission Electron Microscopy Images of [(DipR)$_2$(WR)$_3$]…......……...............………...S29

Dynamic Light Scattering of [DipR]$_5$ ....……….......………...…......……..............………..... .S29

Videos of Cellular Uptake...………………………………...….......…………...…………S30

Confocal Microscopy Images of F´-[K(DipR)$_5$] Treated with Different Concentrations of Endo-cytosis Onhibitors.. S31-33

Plasma Stability of [DipR]$_5$.. S34

Estimate of Peptide Secondary Structures.. S35

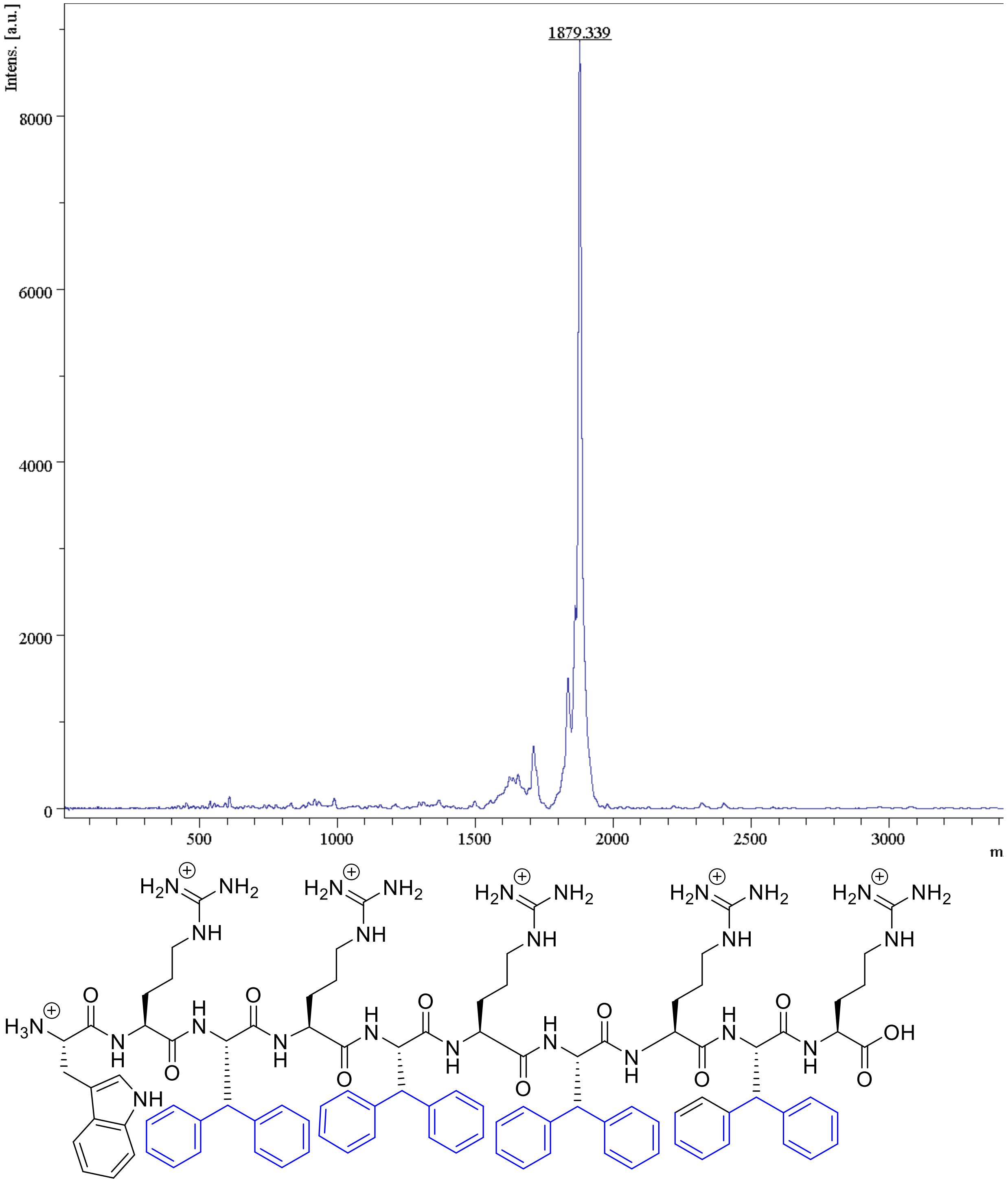

Figure S1. Linear (W-R-Dip-R-Dip-R-Dip-R-Dip-R): MALDI-TOF (m/z) $C_{101}H_{129}N_{29}O_{11}$ Calculated: 1878.3113 Found: 1879.3390 $[M+H]^+$.

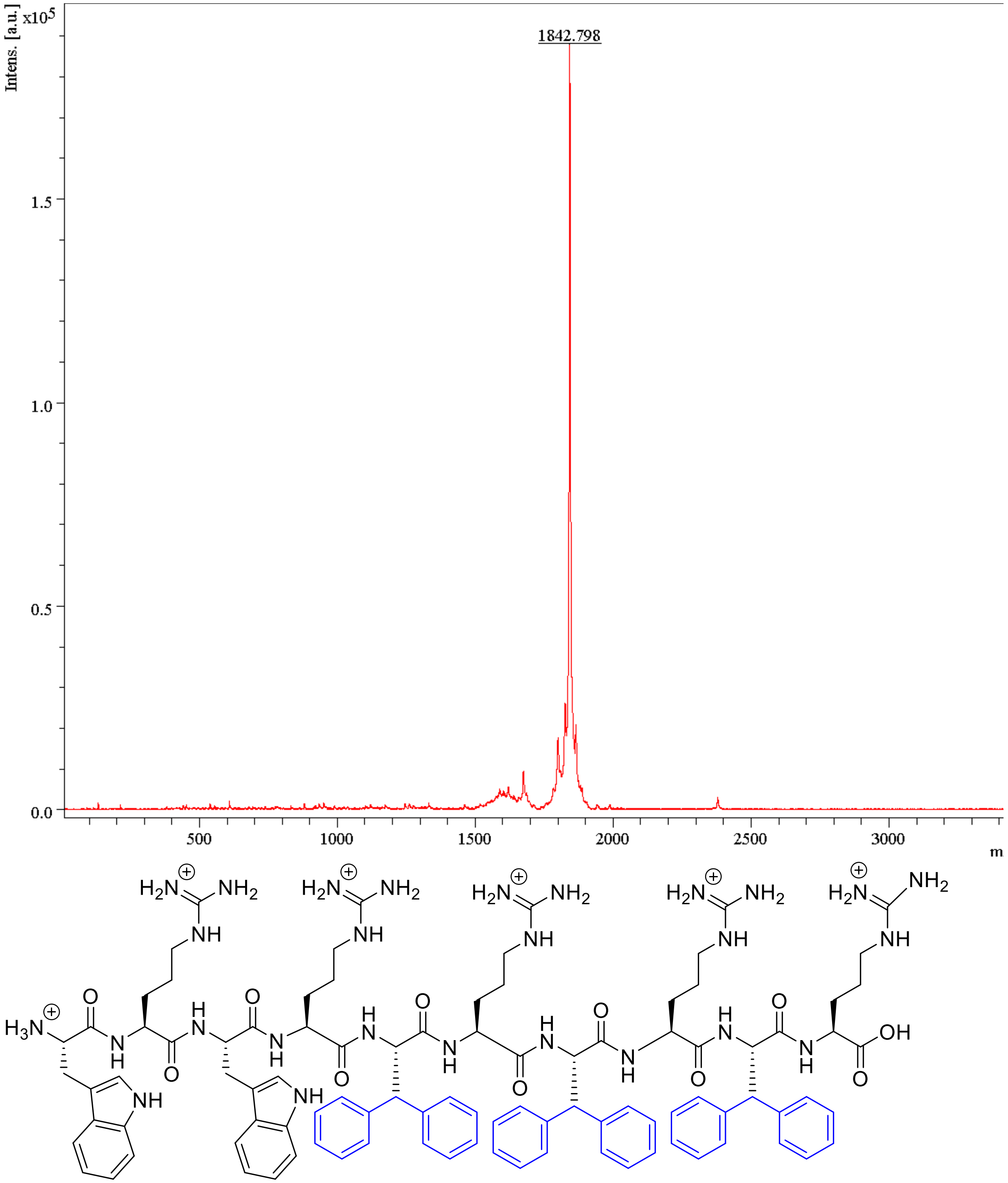

Figure S2. Linear (W-R-W-R-Dip-R-Dip-R-Dip-R): MALDI-TOF (m/z) $C_{97}H_{126}N_{27}O_{11}$ Calculated: 1841.2503 Found: 1842.7980 $[M+H]^+$.

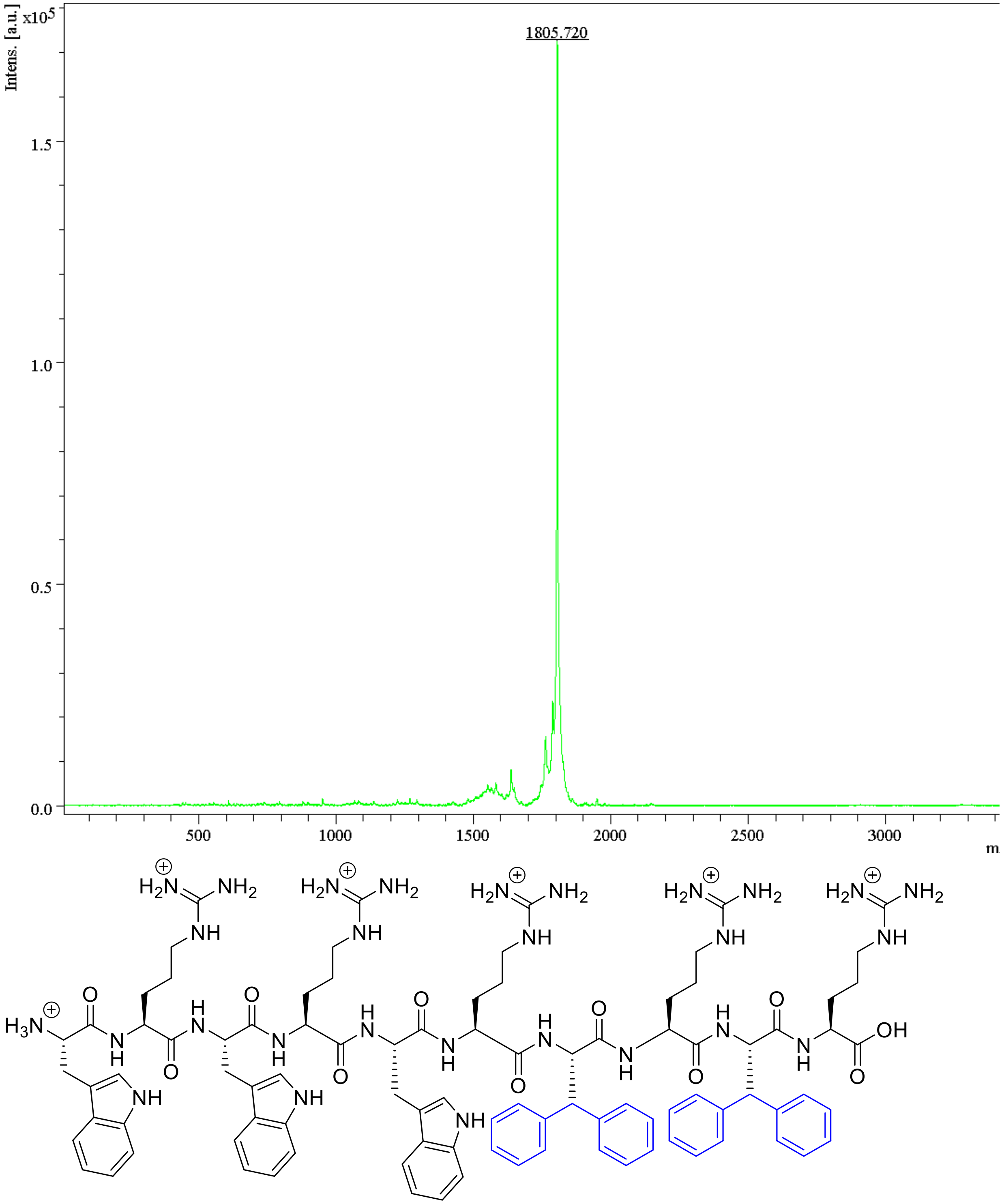

Figure S3. Linear (W-R-W-R-W-R-Dip-R-Dip-R): MALDI-TOF (m/z) $C_{93}H_{123}N_{28}O_{11}$ Calculated: 1804.1893 Found: 1805.7200 $[M+H]^+$.

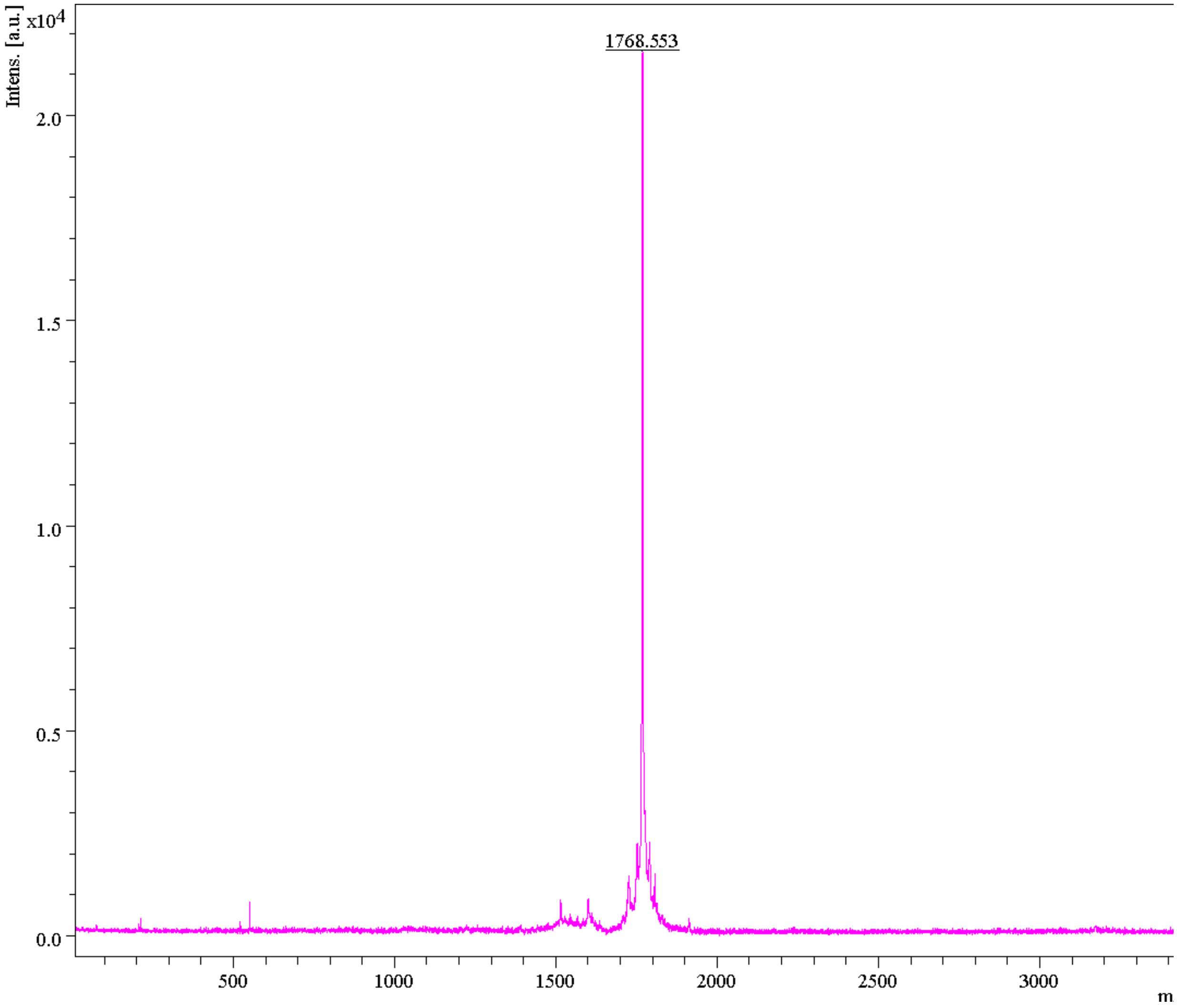

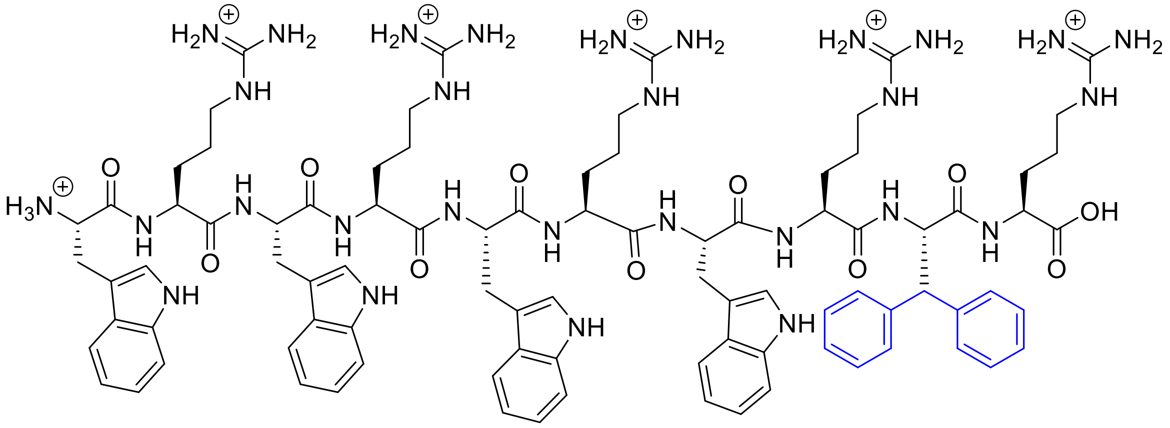

Figure S4. Linear (W-R-W-R-W-R-W-R-Dip-R): MALDI-TOF (m/z) $C_{89}H_{120}N_{29}O_{11}$ Calculated: 1767.1283 Found: 1768.5530 $[M+H]^+$.

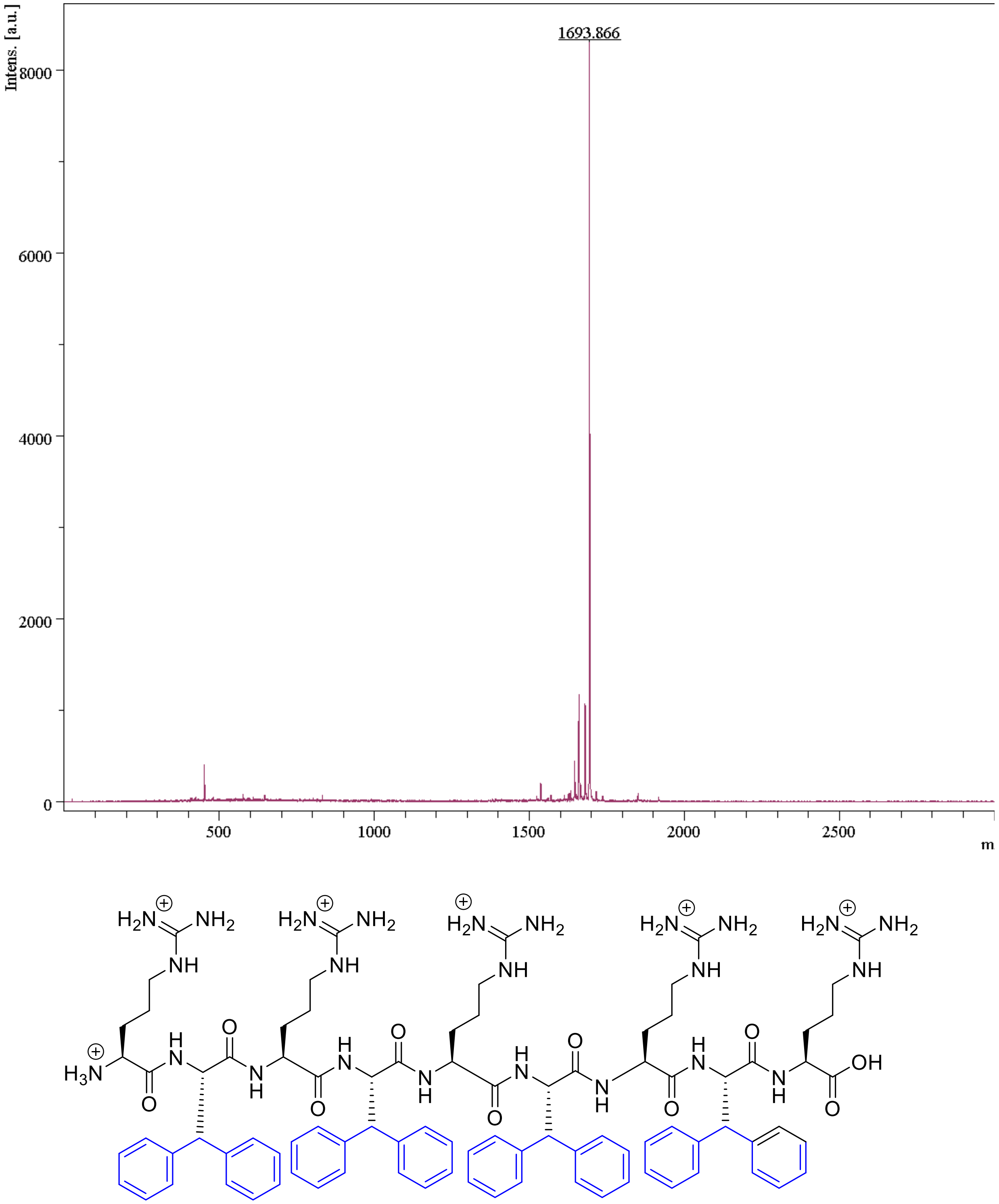

Figure S5. Linear (R-Dip-R-Dip-R-Dip-R-Dip-R): MALDI-TOF (m/z) $C_{90}H_{119}N_{24}O_{10}$ Calculated: 1692.0973 Found: 1693.8660 $[M+H]^+$.

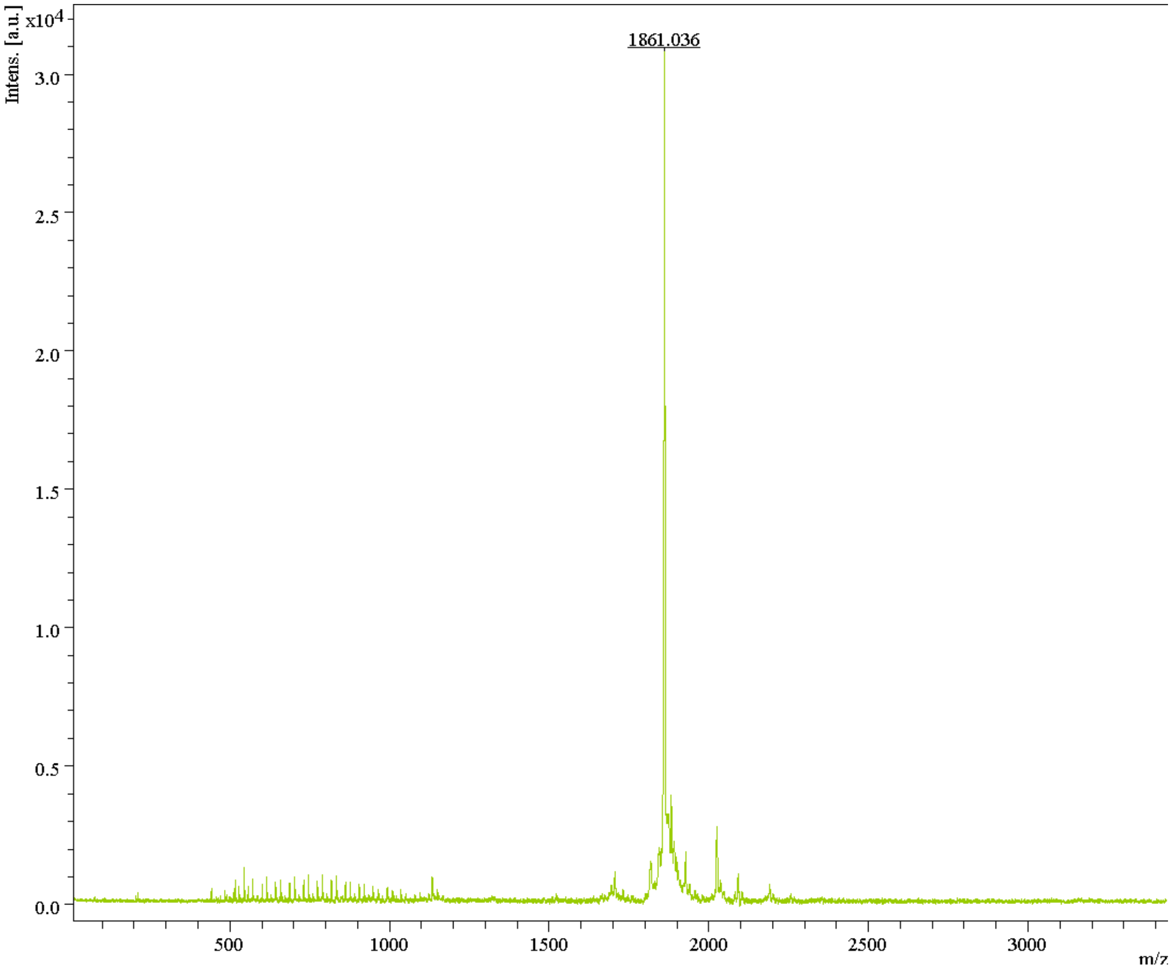

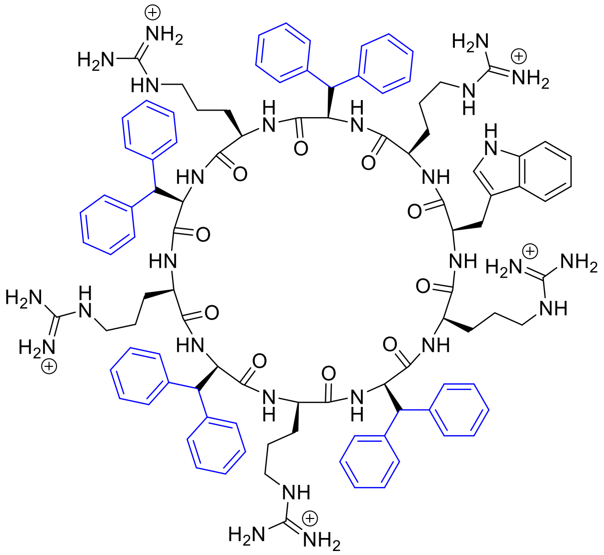

Figure S6. Cyclic [Dip-R-W-R-Dip-R-Dip-R-Dip-R]: MALDI-TOF (m/z) $C_{101}H_{127}N_{26}O_{10}$ Calculated: 1860.2963 Found: 1861.0360 $[M+H]^+$.

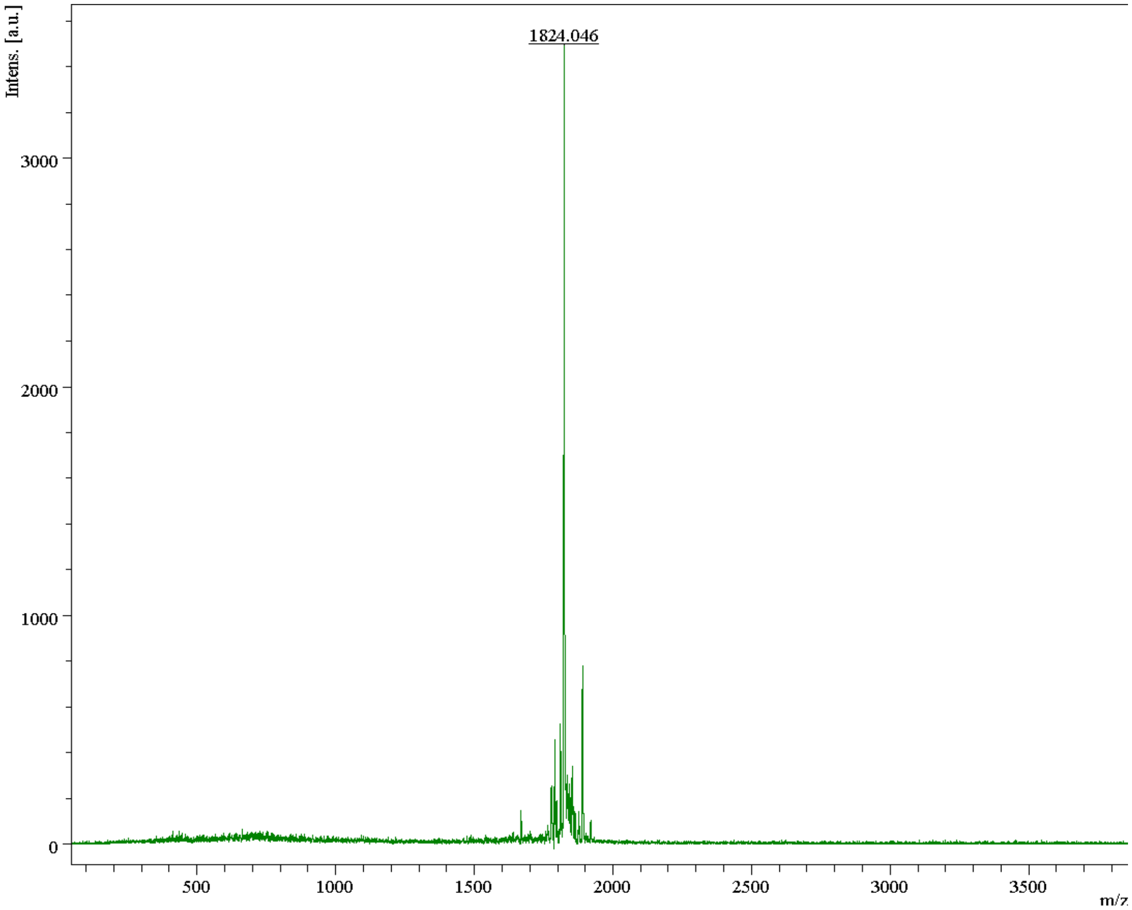

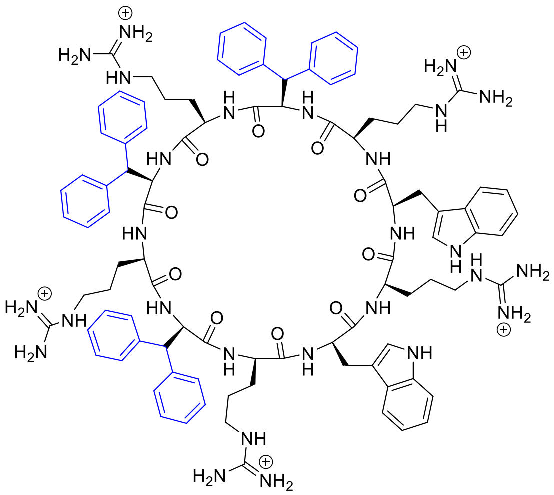

Figure S7. Cyclic [Dip-R-W-R-W-R-Dip-R-Dip-R]: MALDI-TOF (m/z) $C_{97}H_{124}N_{27}O_{10}$ Calculated: 1823.2353 Found: 1824.0460 $[M+H]^+$.

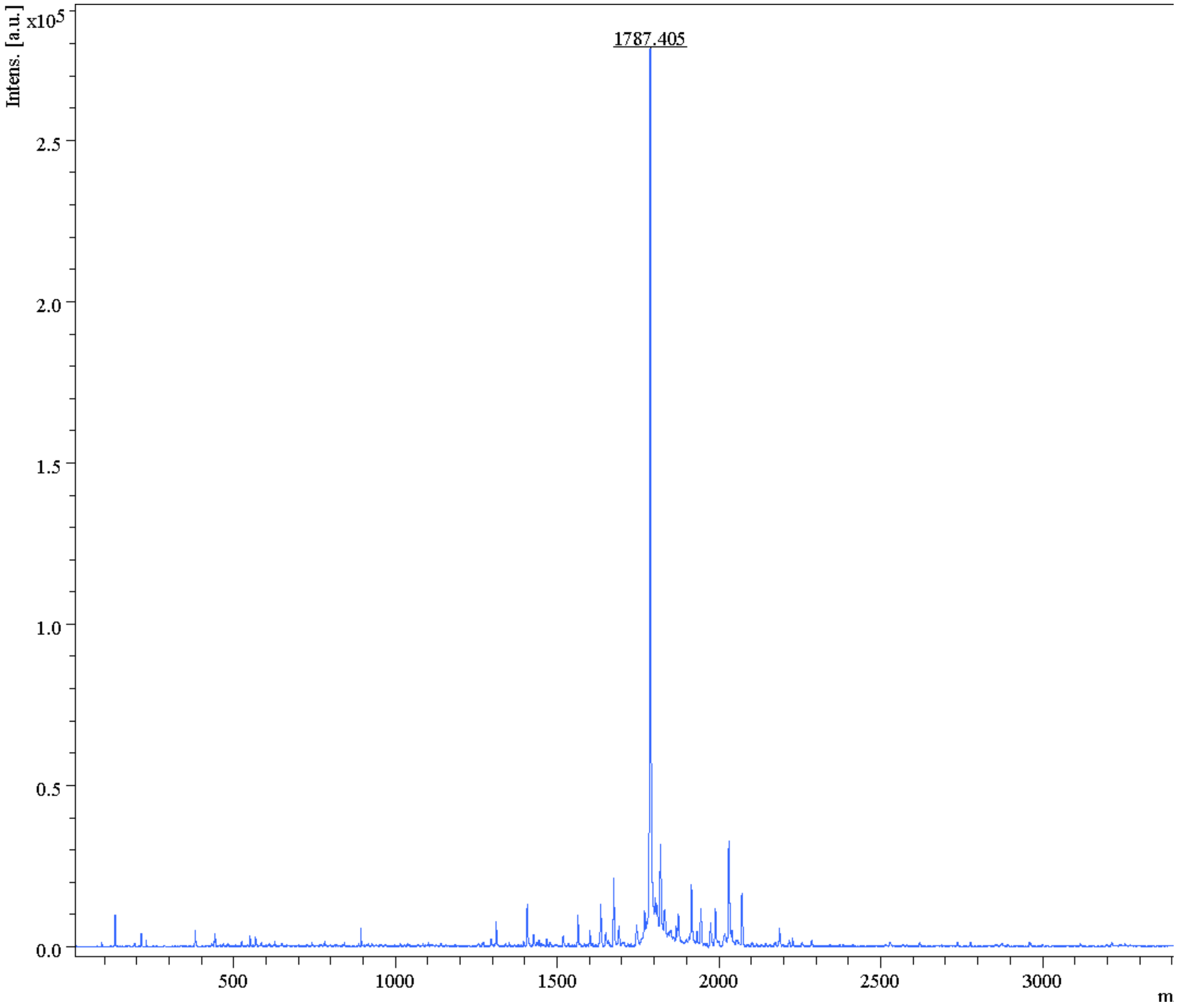

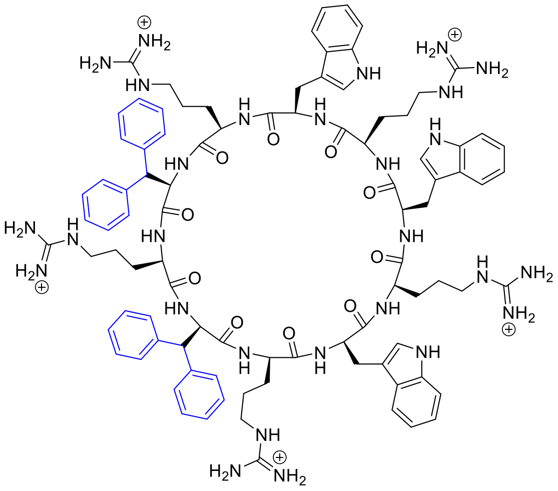

Figure S8. Cyclic [Dip-R-W-R-W-R-W-R-Dip-R]: MALDI-TOF (m/z) $C_{93}H_{121}N_{28}O_{10}$ Calculated: 1786.1743 Found: 1787.4050 $[M+H]^+$.

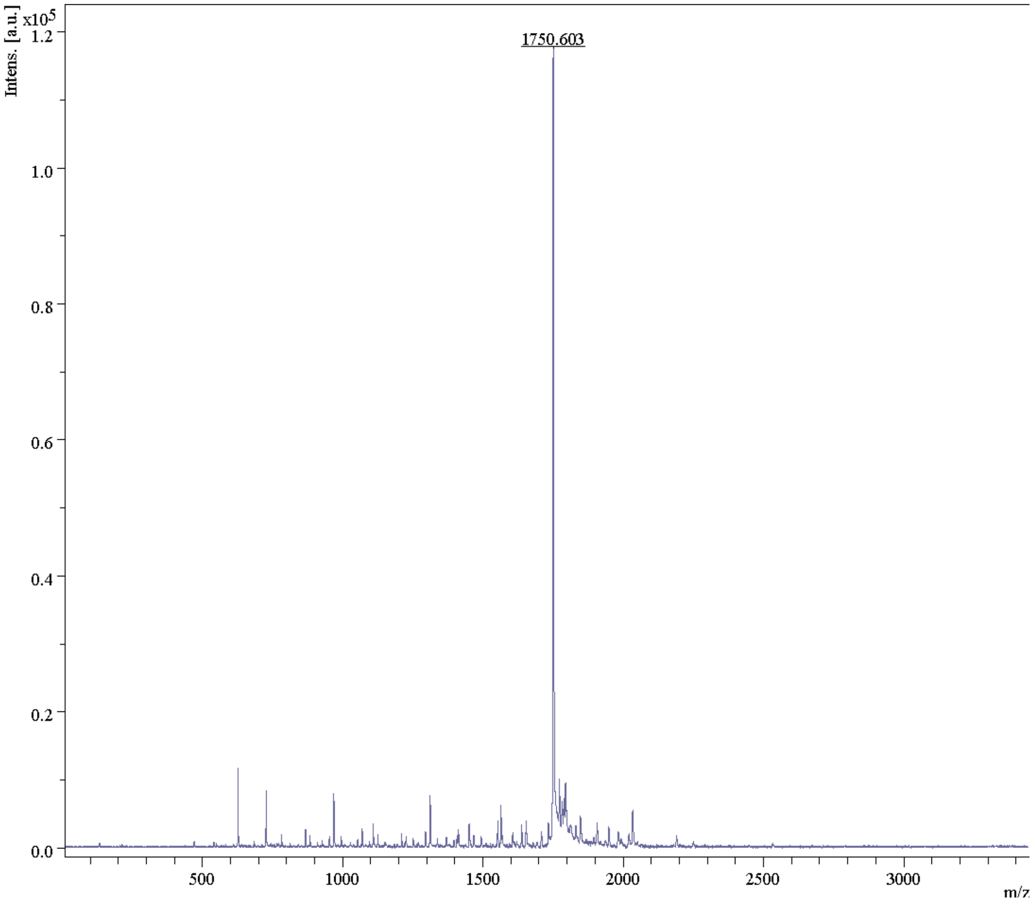

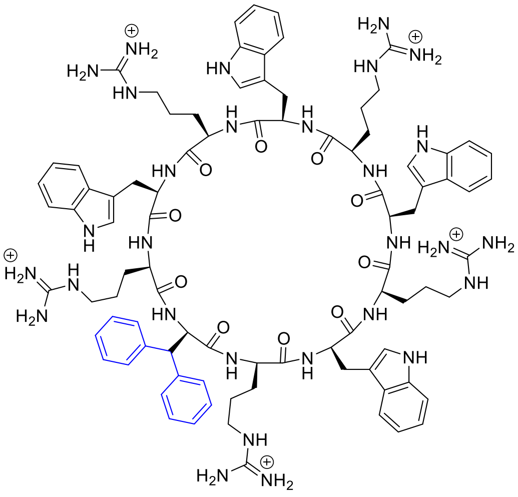

Figure S9. Cyclic [Dip-R-W-R-W-R-W-R-W-R]: MALDI-TOF (m/z) $C_{89}H_{118}N_{29}O_{10}$ Calculated: 1749.1133 Found: 1750.6030 $[M+H]^+$.

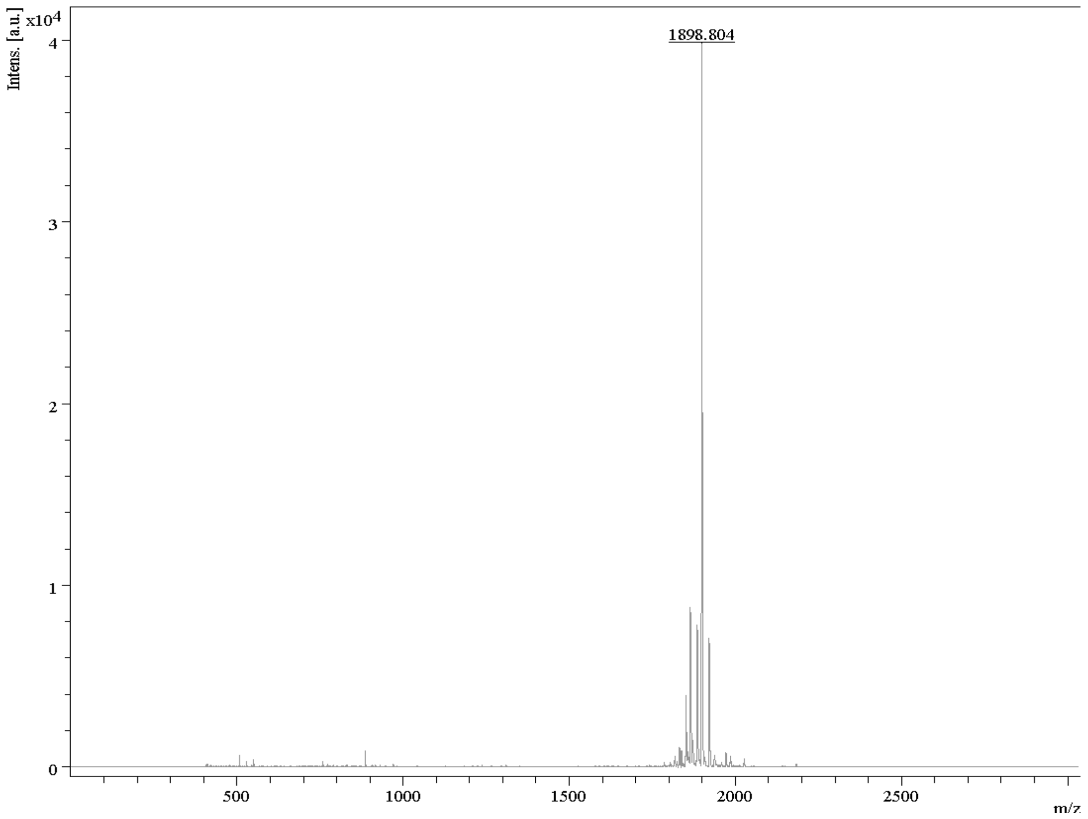

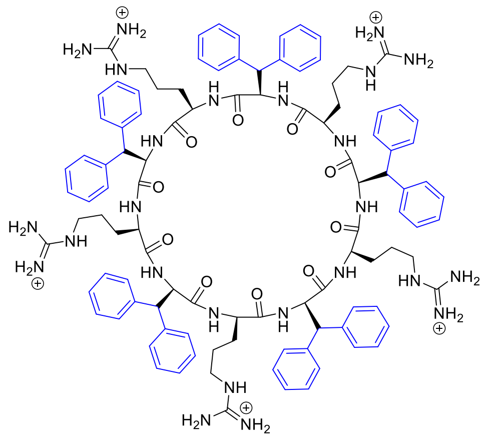

Figure S10. Cyclic [Dip-R-Dip-R-Dip-R-Dip-R-Dip-R]: MALDI-TOF (m/z) $C_{105}H_{130}N_{25}O_{10}$ Calculated: 1897.3573 Found: 1898.8040 [M+H]$^+$.

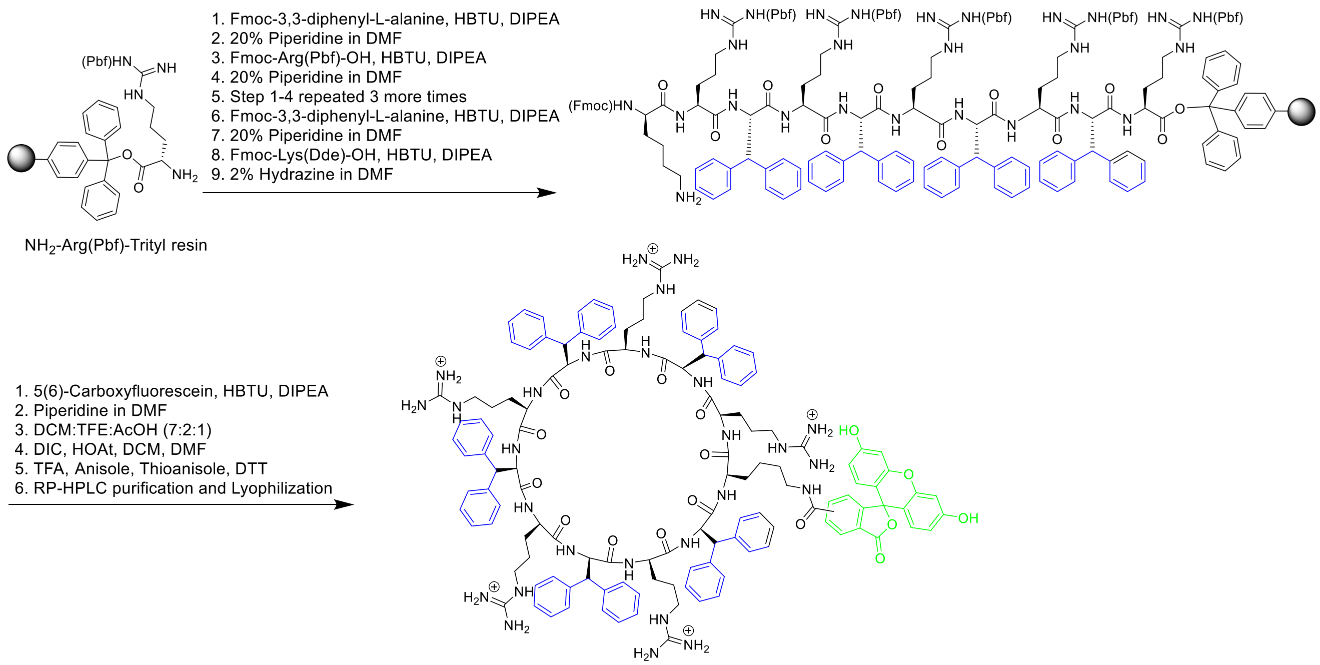

Figure S11. Synthesis of Fluorescence-labeled [K(DipR)5].

Analytical HPLC Methods

Method A

Pump A: 0.1% Trifluoroacetic in 100% Water

Pump B: 0.1% Trifluoroacetic in 100% Acetonitrile

Total Flow: 1 ml/min

Time	Module	Action	Value
0.00	Pumps	B.Conc	20
25.00	Pumps	B.Conc	80
25.01	Pumps	B.Conc	100
30.00	Pumps	B.Conc	100
30.01	Pumps	Stop	

Method B

Pump A: 0.1% Trifluoroacetic in 100% Water

Pump B : 0.1% Trifluoroacetic in 100% Acetonitrile

Total Flow: 1 ml/min

Time	Module	Action	Value
0.00	Pumps	B.Conc	25
25.00	Pumps	B.Conc	85
25.01	Pumps	B.Conc	100
30.00	Pumps	B.Conc	100
30.01	Pumps	Stop	

Method C

Pump A: 0.1% Formic Acid in 100% Water

Pump B: 0.1% Formic Acid in 100% Acetonitrile

Total Flow: 2 ml/min

Time	Module	Action	Value

2.00	Pumps	B.Conc	5
15.00	Pumps	B.Conc	95
20.00	Pumps	B.Conc	95
21.00	Pumps	B.Conc	5
23.00	Pumps	B.Conc	5
23.00	Pumps	Stop	

Method D

Pump A: 0.1% Formic Acid in 100% Water

Pump B : 0.1% Formic Acid in 100% Acetonitrile

Total Flow: 1 ml/min

Time	Module	Action	Value
5.00	Pumps	B.Conc	5
30.00	Pumps	B.Conc	95
35.00	Pumps	B.Conc	95
36.00	Pumps	B.Conc	5
40.00	Pumps	B.Conc	5
40.00	Pumps	Stop	

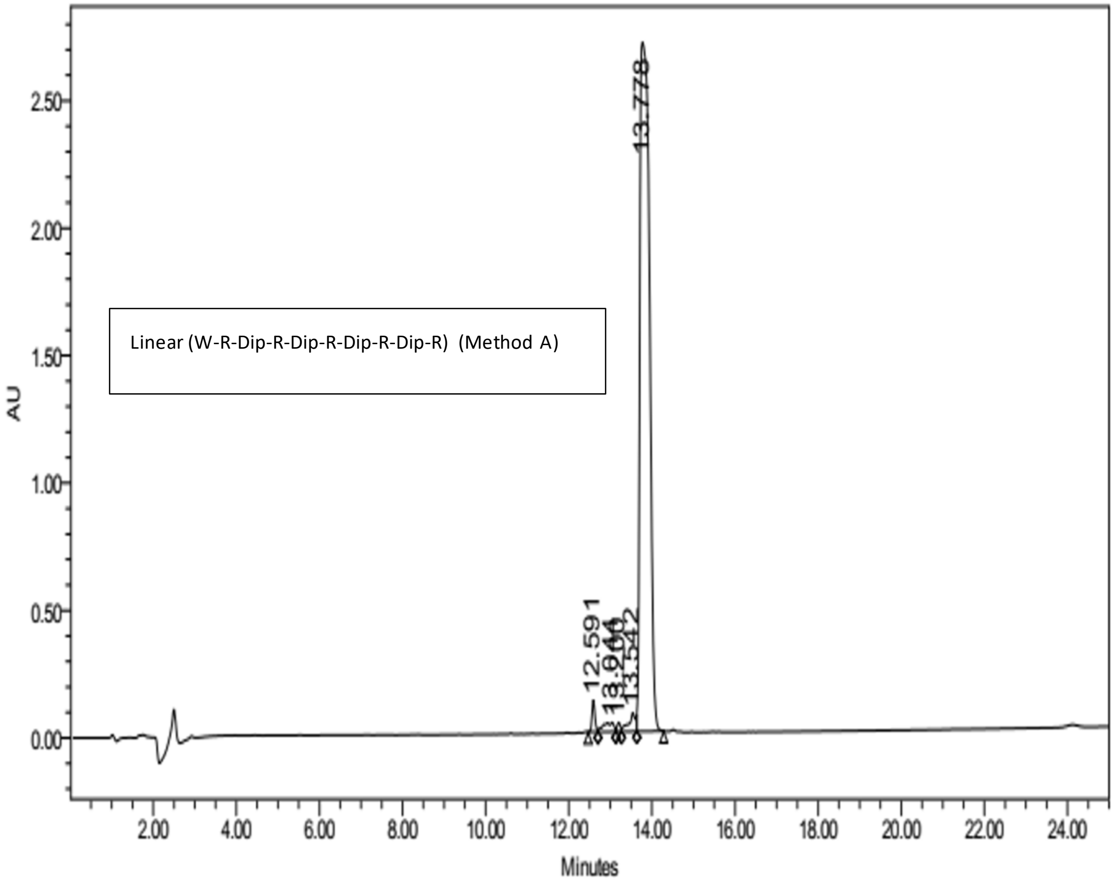
Linear (W-R-Dip-R-Dip-R-Dip-R-Dip-R) (Method A)
AU
Minutes
13.778
12.591
13.542
2.50
2.00
1.50
1.00
0.50
0.00
2.00
4.00
6.00
8.00
10.00
12.00
14.00
16.00
18.00
20.00
22.00
24.00

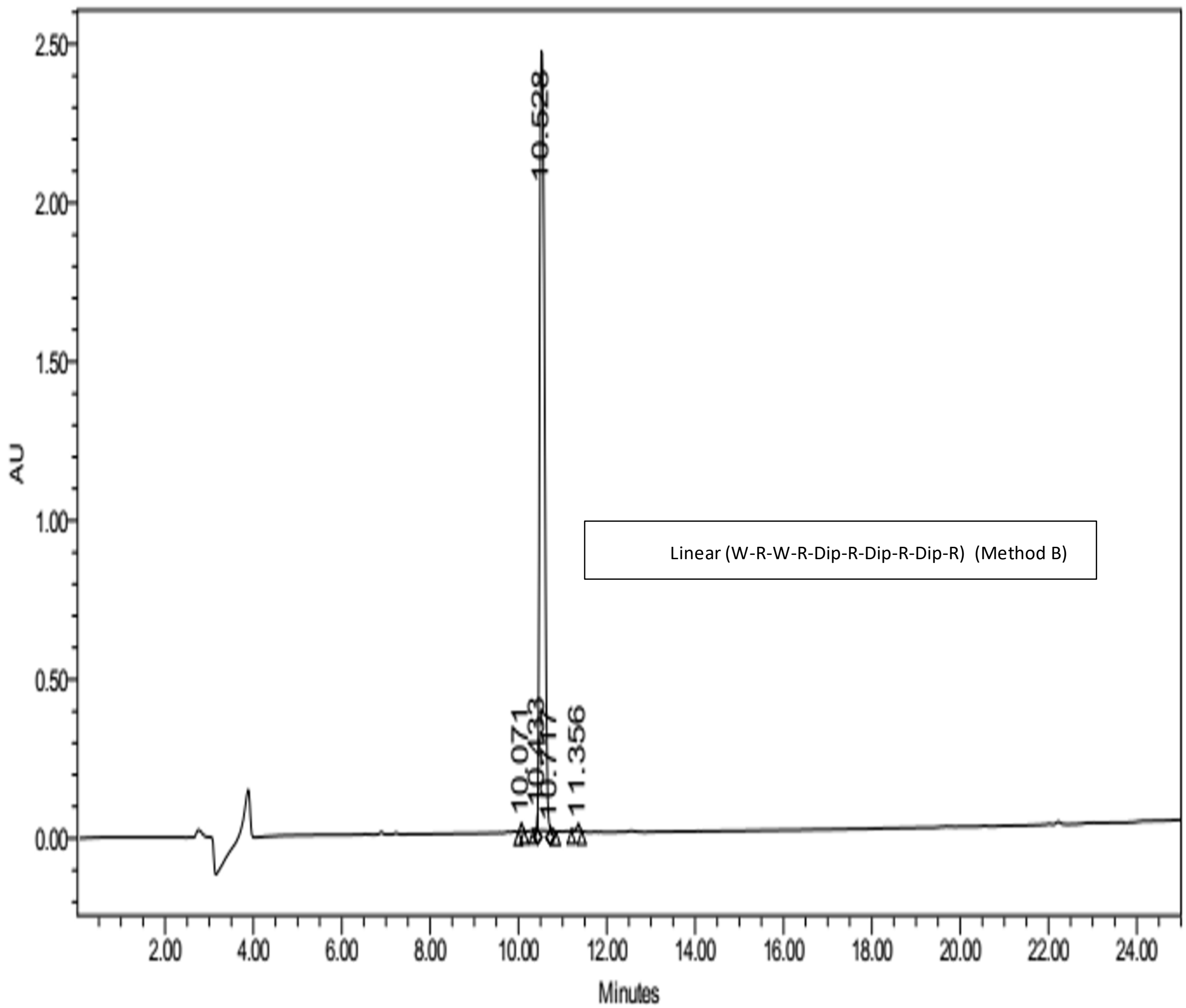
10.528
10.071
10.433
10.717
11.356
AU
Minutes
Linear (W-R-W-R-Dip-R-Dip-R-Dip-R) (Method B)

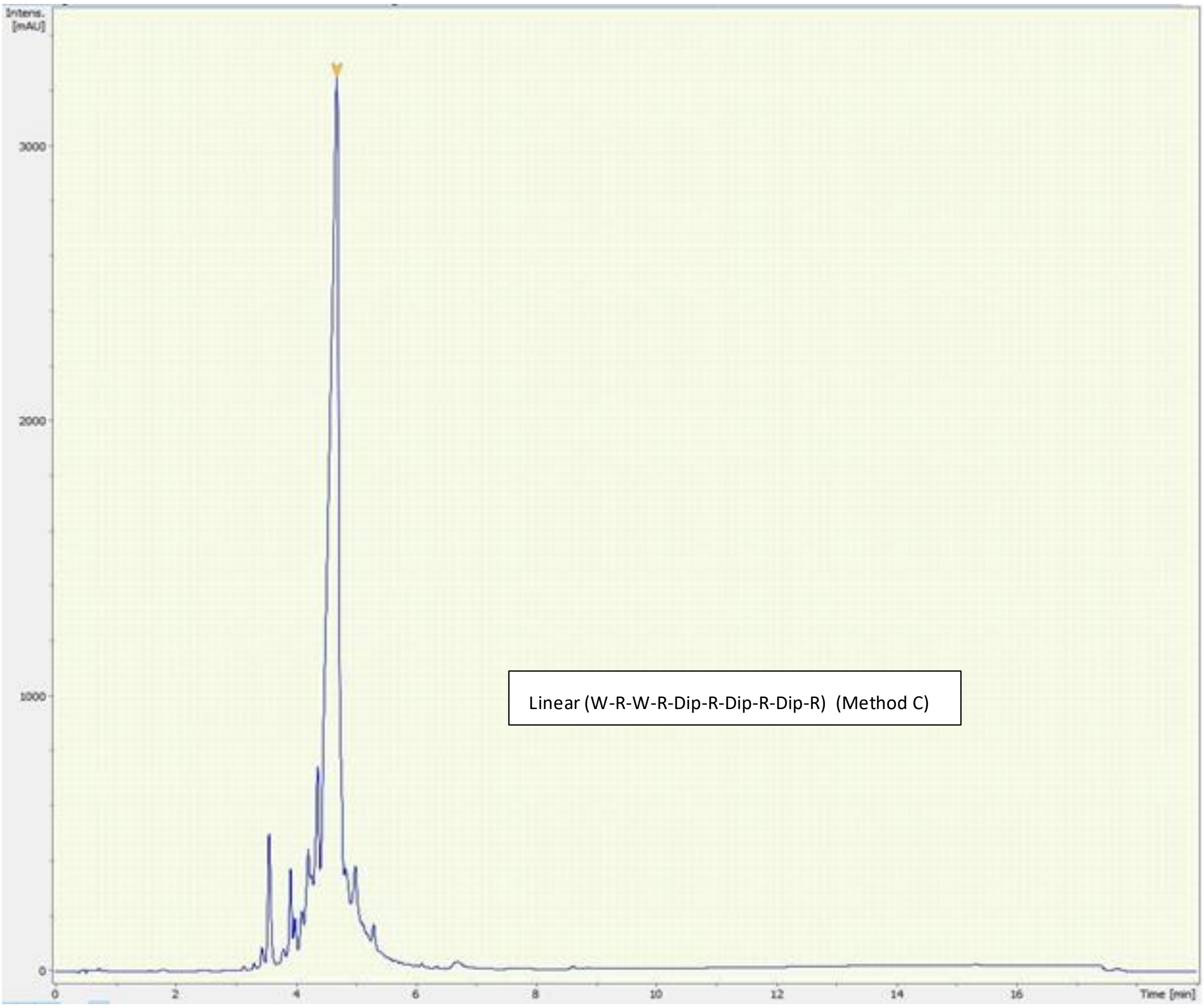
Intens. [mAU]
3000
2000
1000
0
0
2
4
6
8
10
12
14
16
Time [min]
Linear (W-R-W-R-Dip-R-Dip-R-Dip-R) (Method C)

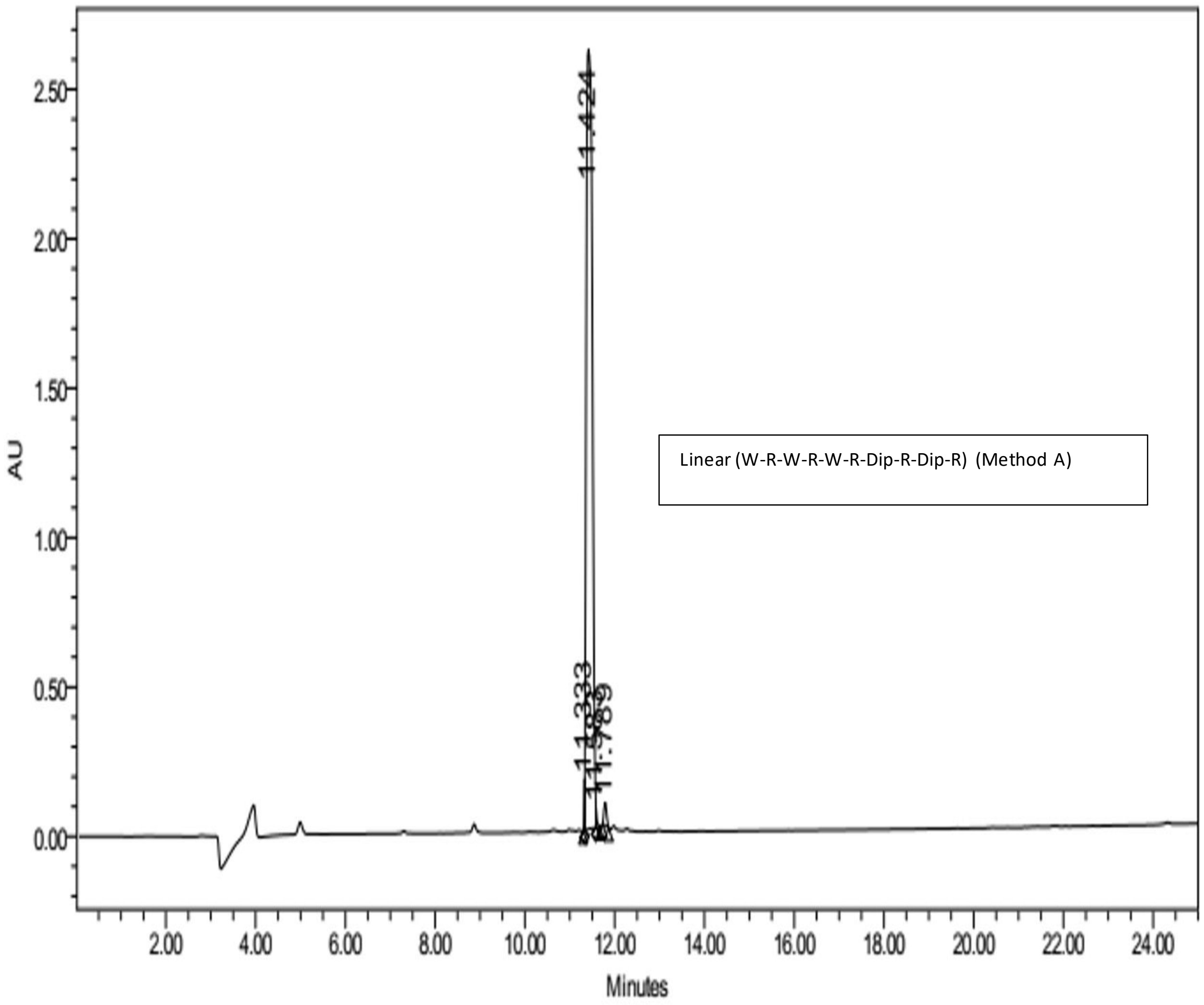

2.50
2.00
1.50
1.00
0.50
0.00
AU
11.424
11.333
11.789
Linear (W-R-W-R-W-R-Dip-R-Dip-R) (Method A)
2.00
4.00
6.00
8.00
10.00
12.00
14.00
16.00
18.00
20.00
22.00
24.00
Minutes

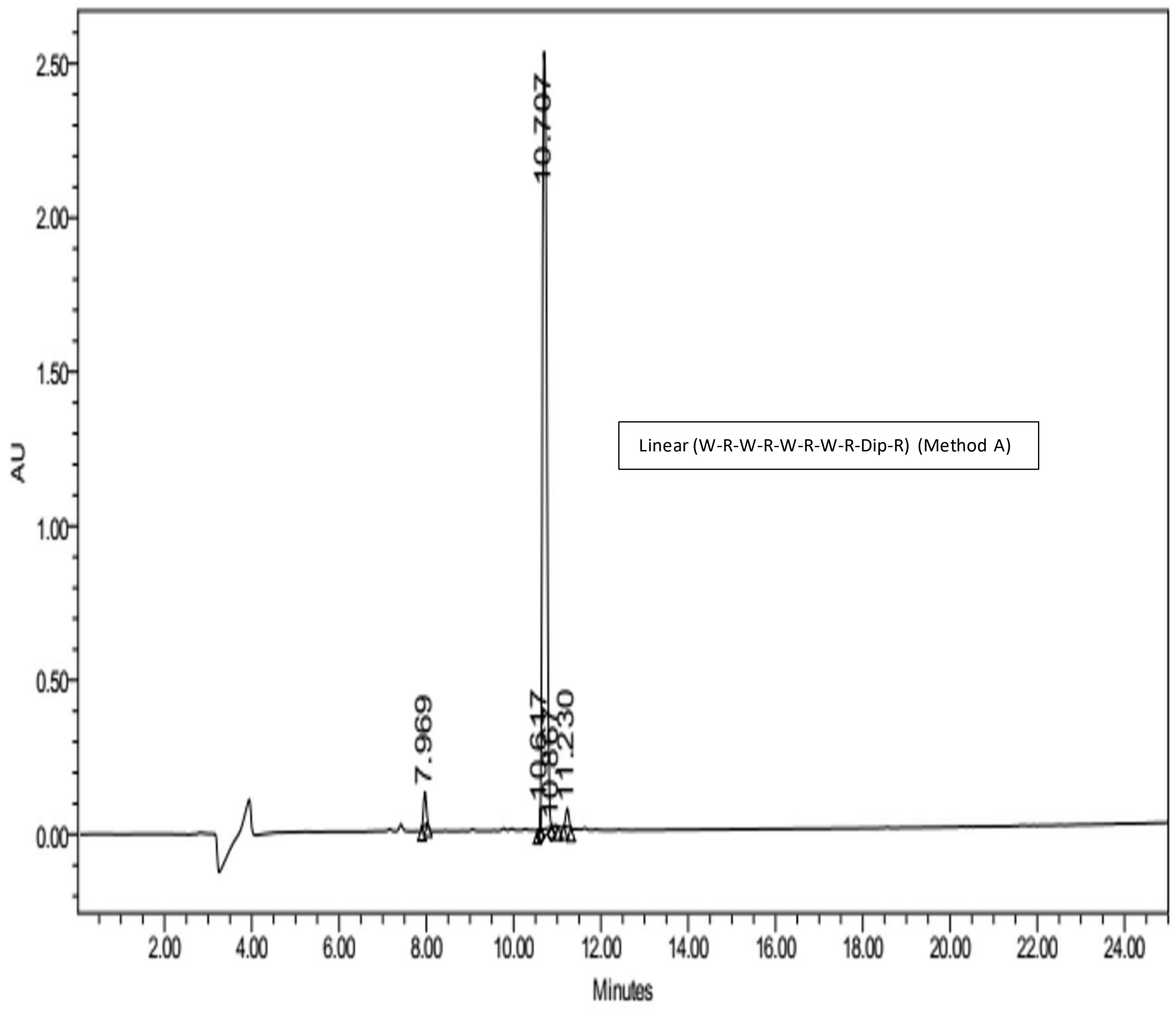

2.50
2.00
1.50
1.00
0.50
0.00
AU
10.707
7.969
10.617
10.867
11.230
Linear (W-R-W-R-W-R-W-R-Dip-R) (Method A)
2.00
4.00
6.00
8.00
10.00
12.00
14.00
16.00
18.00
20.00
22.00
24.00
Minutes

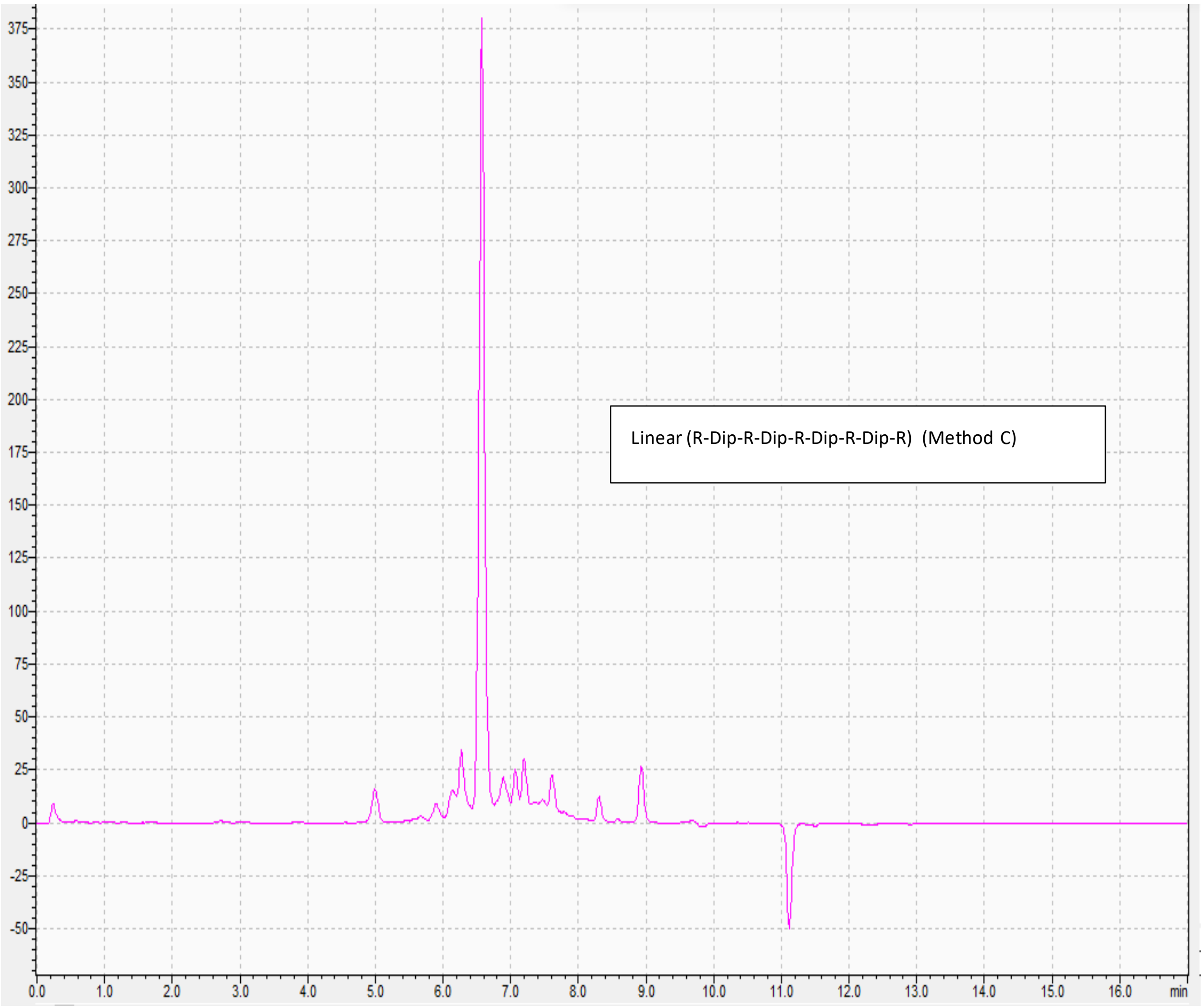

Linear (R-Dip-R-Dip-R-Dip-R-Dip-R) (Method C)
375
350
325
300
275
250
225
200
175
150
125
100
75
50
25
0
-25
-50
0.0
1.0
2.0
3.0
4.0
5.0
6.0
7.0
8.0
9.0
10.0
11.0
12.0
13.0
14.0
15.0
16.0
min

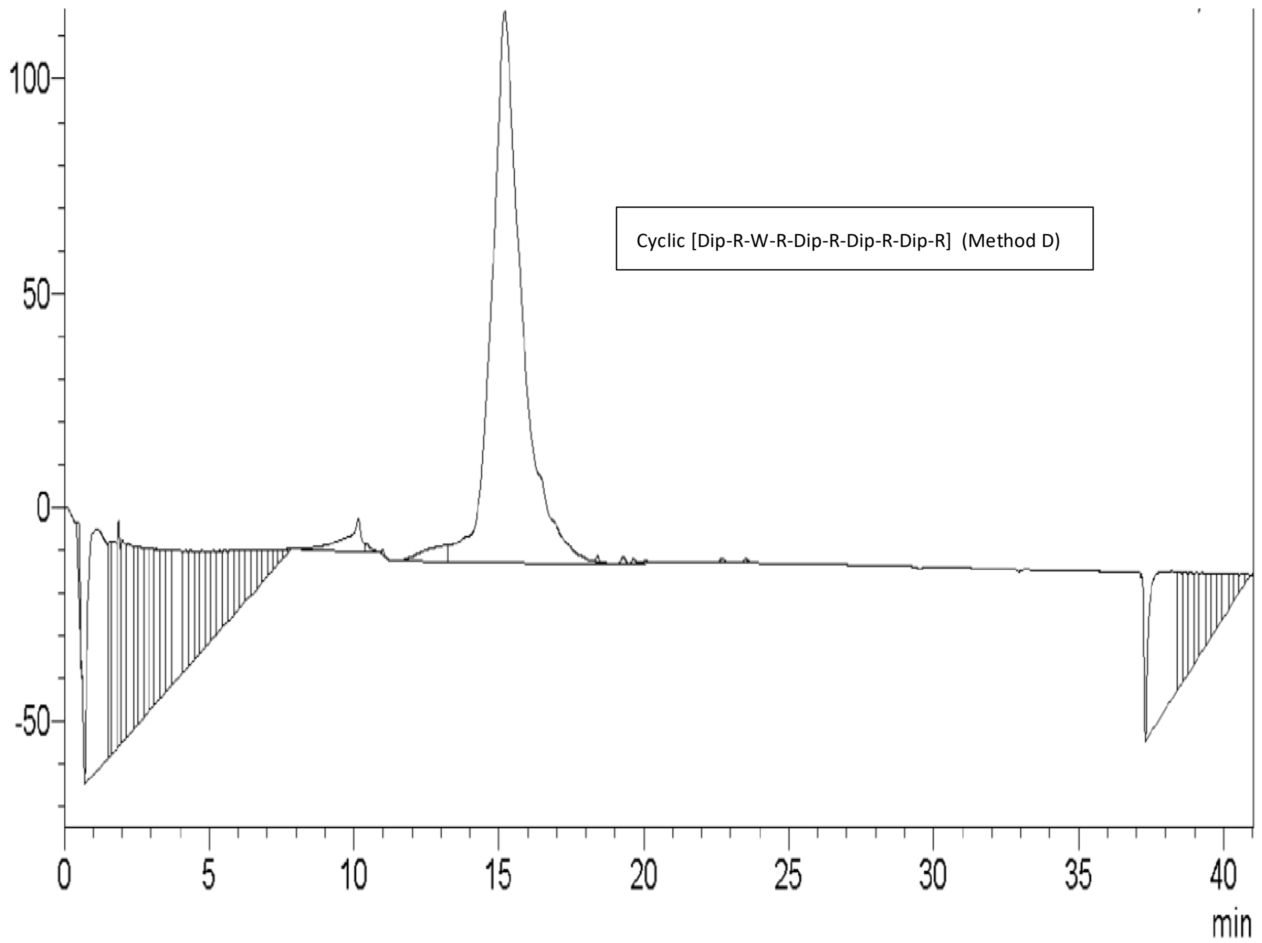

Cyclic [Dip-R-W-R-Dip-R-Dip-R-Dip-R] (Method D)
100
50
0
-50
0
5
10
15
20
25
30
35
40
min

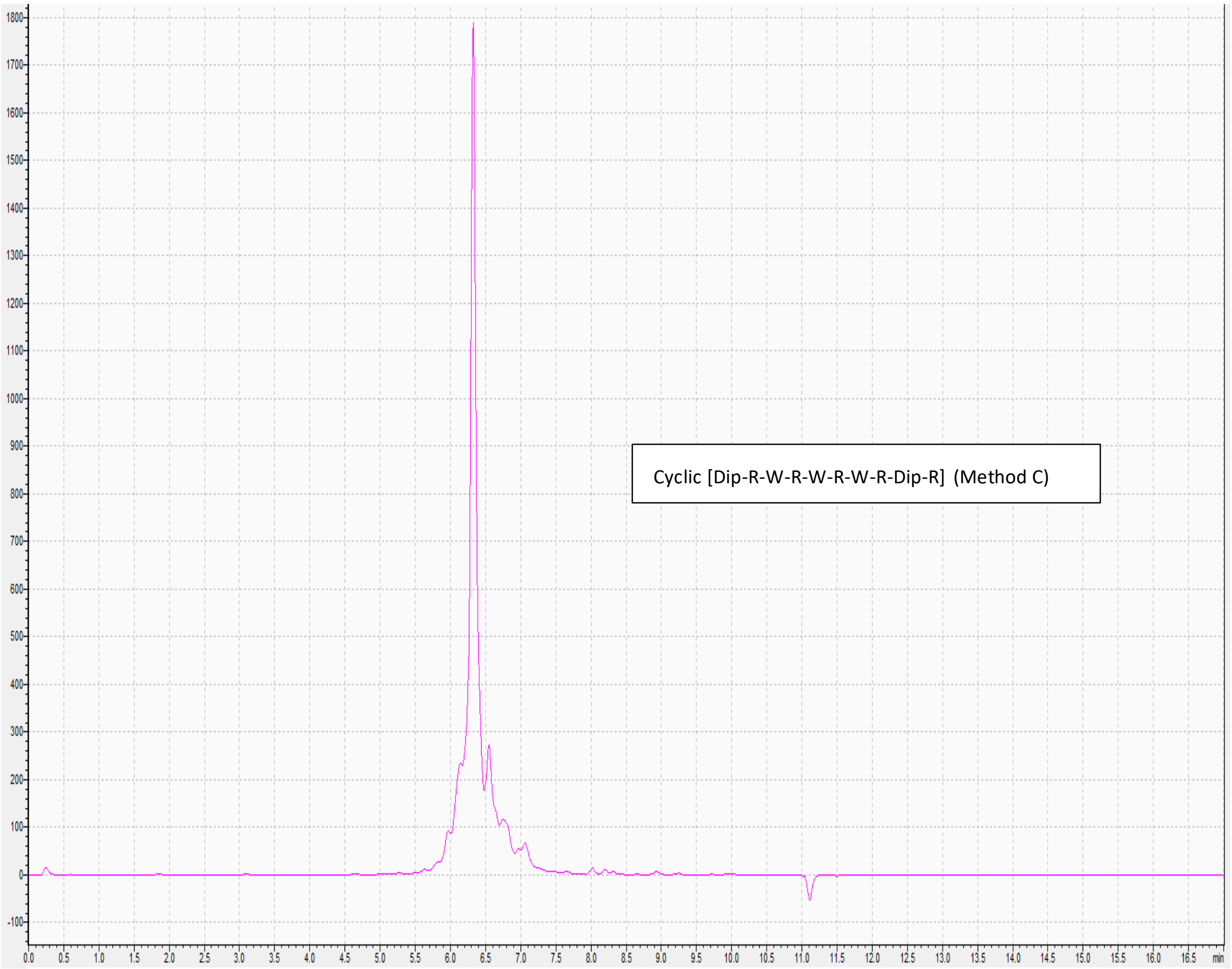

Cyclic [Dip-R-W-R-W-R-W-R-Dip-R] (Method C)
min

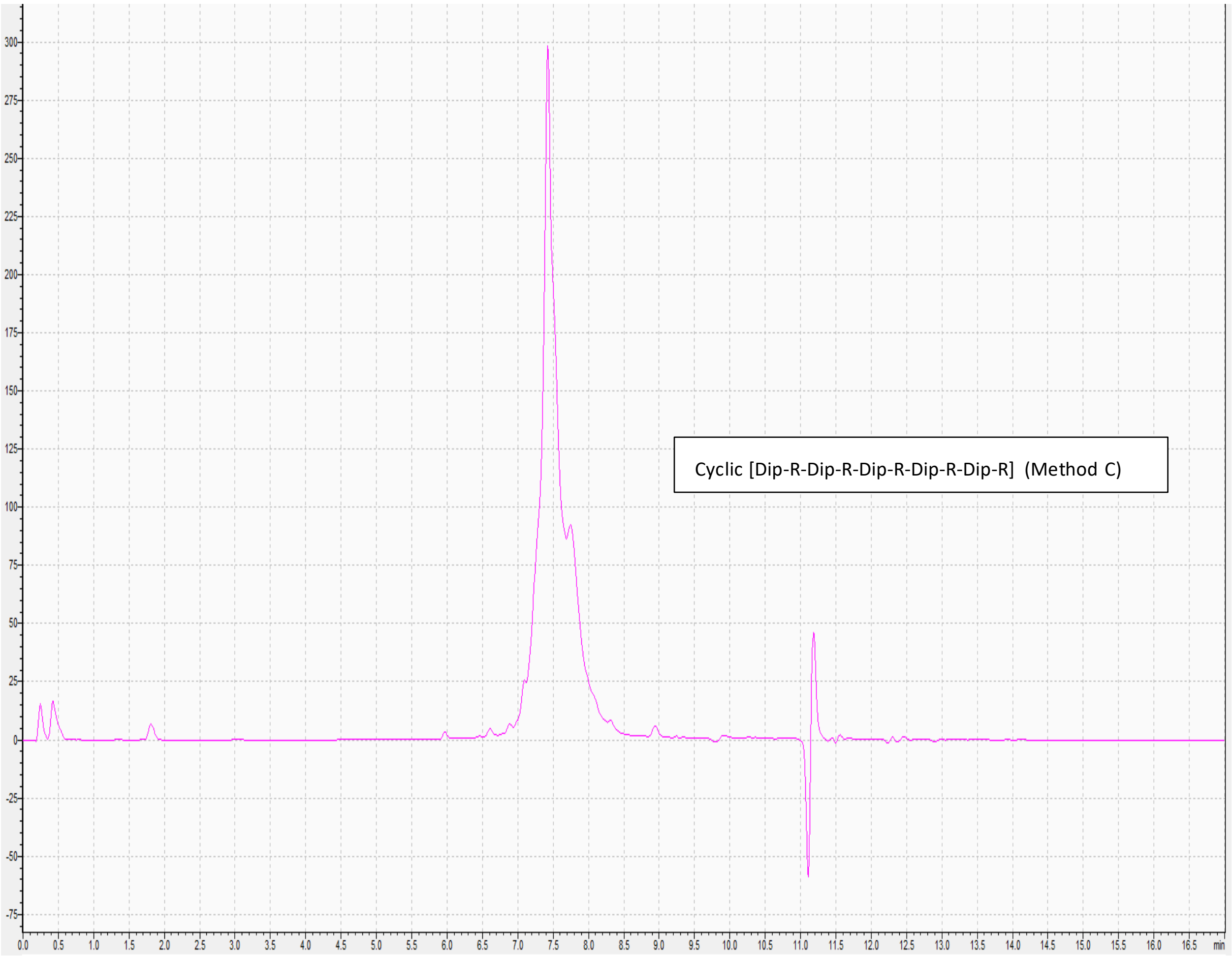
Cyclic [Dip-R-Dip-R-Dip-R-Dip-R-Dip-R] (Method C)
min

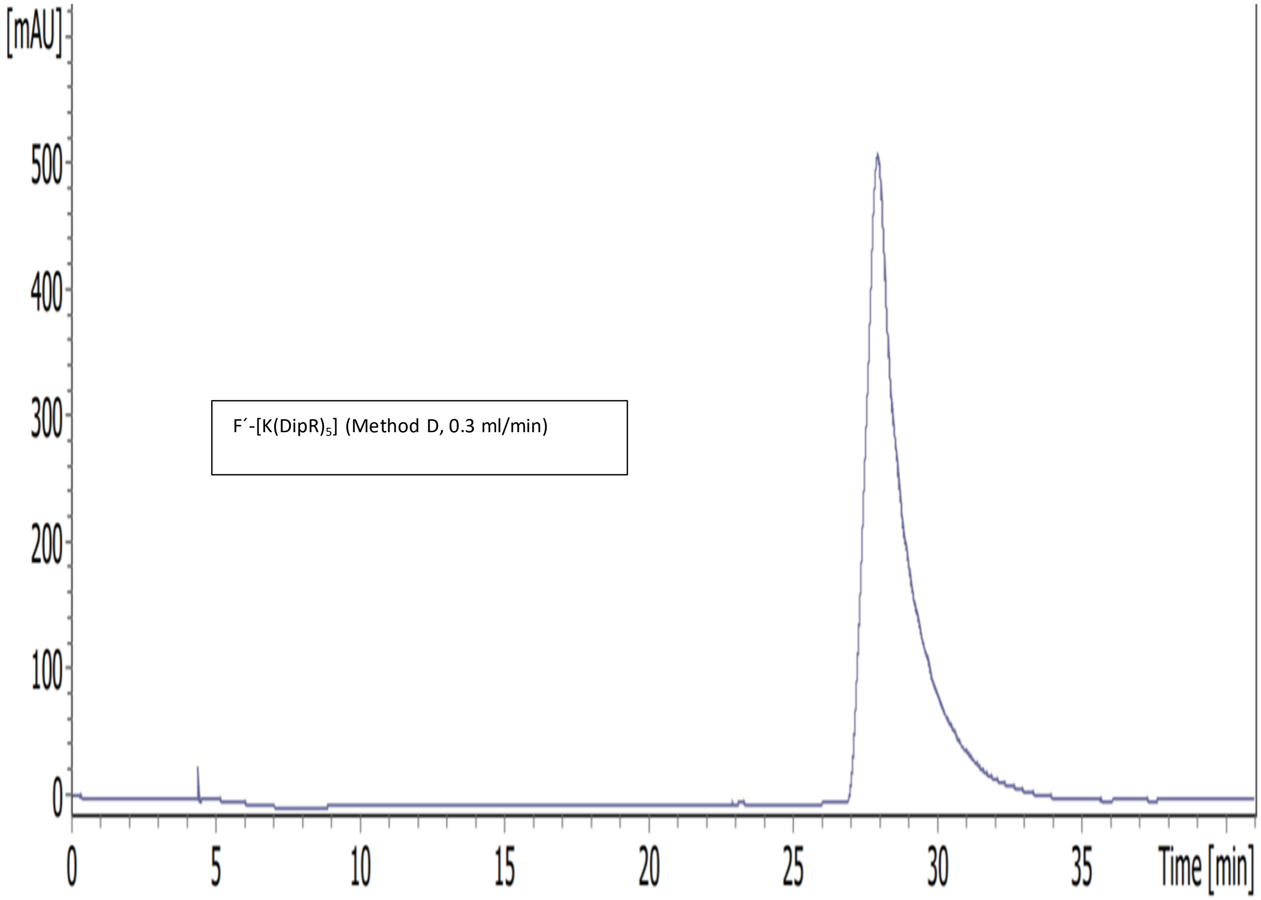

Figure S12. Chromatography Spectra of peptides.

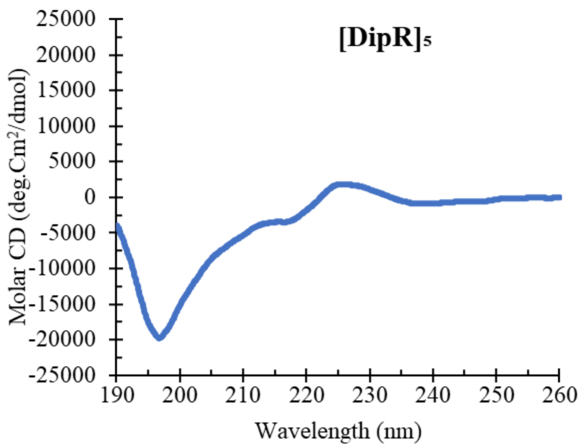
[DipR]5
Molar CD (deg.Cm2/dmol)
25000
20000
15000
10000
5000
0
-5000
-10000
-15000
-20000
-25000
190 200 210 220 230 240 250 260
Wavelength (nm)

(**A**)

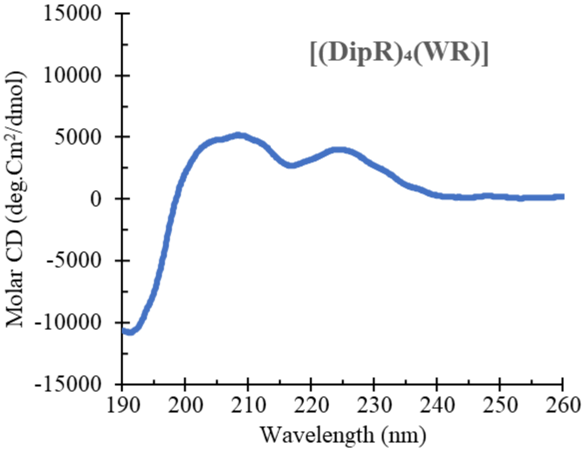
[(DipR)4(WR)]
Molar CD (deg.Cm2/dmol)
15000
10000
5000
0
-5000
-10000
-15000
190 200 210 220 230 240 250 260
Wavelength (nm)

(**B**)

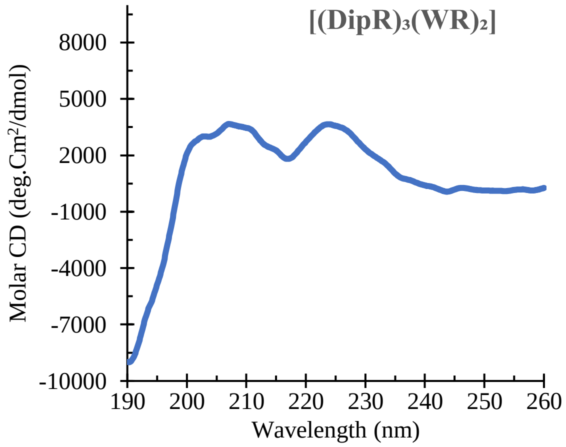
[(DipR)3(WR)2]
Molar CD (deg.Cm2/dmol)
8000
5000
2000
-1000
-4000
-7000
-10000
190 200 210 220 230 240 250 260
Wavelength (nm)

(**C**)

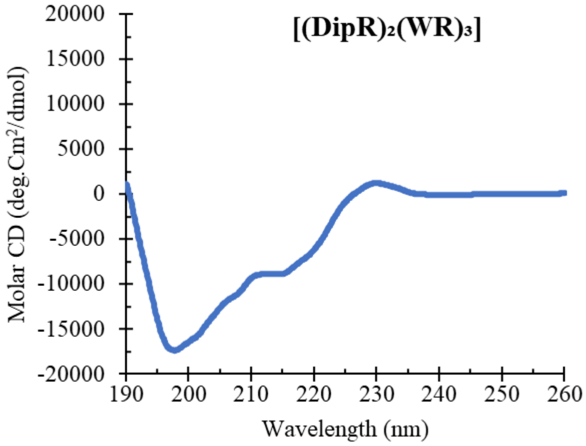
[(DipR)2(WR)3]
Molar CD (deg.Cm2/dmol)
20000
15000
10000
5000
0
-5000
-10000
-15000
-20000
190 200 210 220 230 240 250 260
Wavelength (nm)

(**D**)

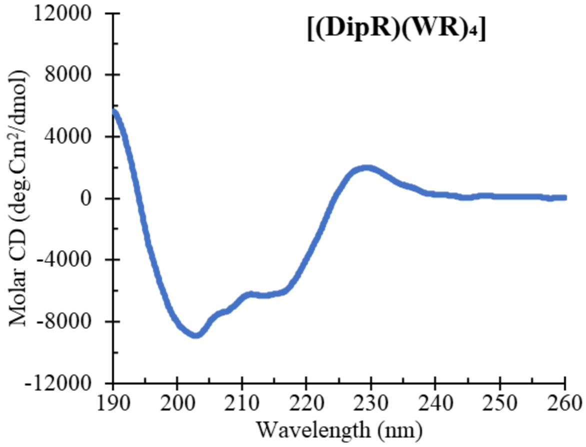
[(DipR)(WR)4]
Molar CD (deg.Cm2/dmol)
12000
8000
4000
0
-4000
-8000
-12000
190 200 210 220 230 240 250 260
Wavelength (nm)

(**E**)

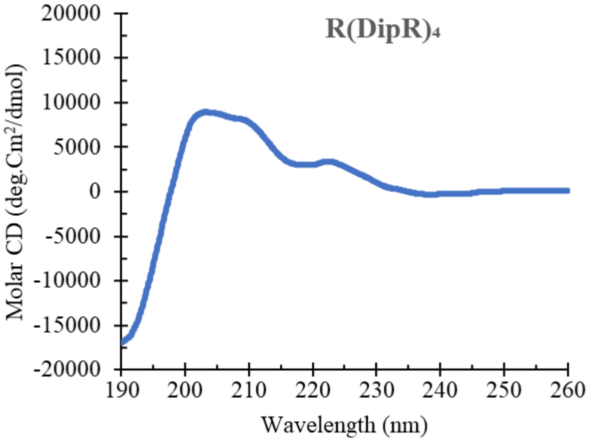
R(DipR)4
Molar CD (deg.Cm2/dmol)
20000
15000
10000
5000
0
-5000
-10000
-15000
-20000
190 200 210 220 230 240 250 260
Wavelength (nm)

(**F**)

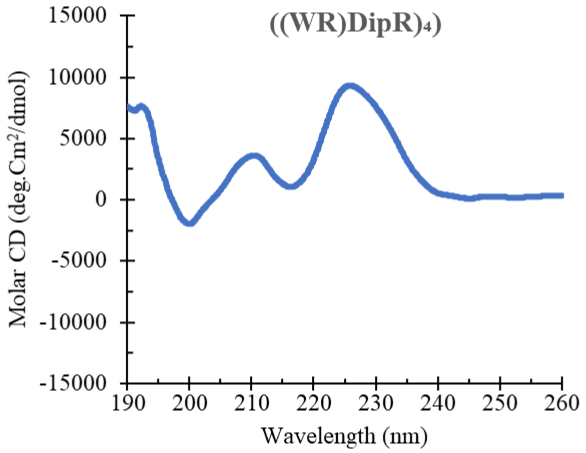

(G)

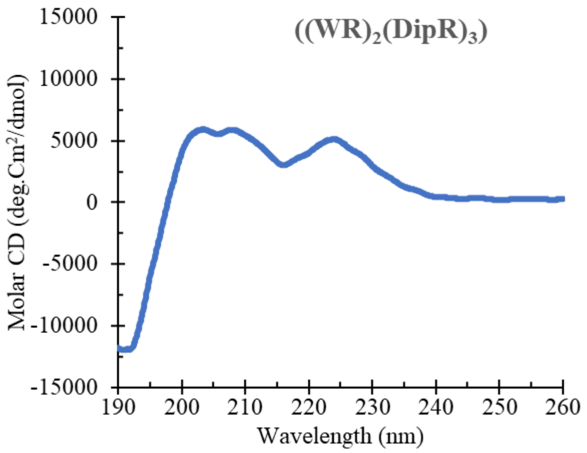

(H)

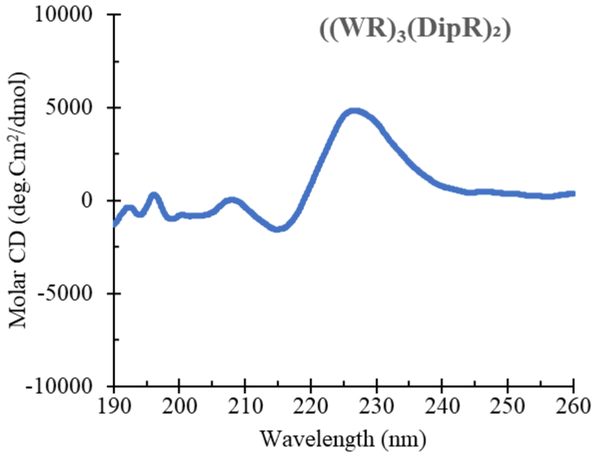

(I)

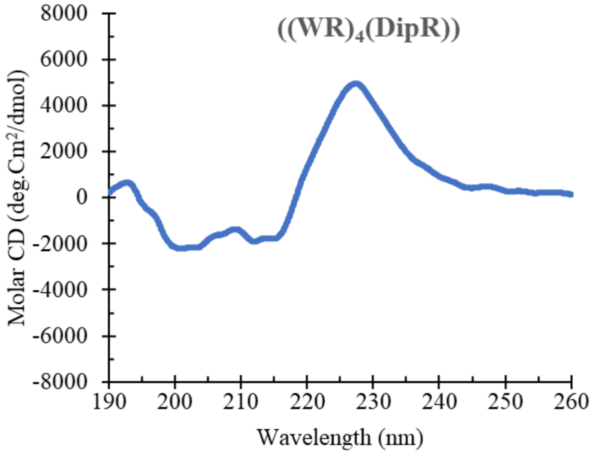

(J)

Figure S13. Circular dichroism spectra of (**A**) $[DipR]_5$; (**B**) $[(DipR)_4(WR)]$; (**C**) $[(DipR)_3(WR)_2]$; (**D**) $[(DipR)_2(WR)_3]$; (**E**) $[(DipR)(WR)_4]$; (**F**) $R(DipR)_4$; (**G**) $((WR)(DipR)_4)$; (**H**) $((WR)_2(DipR)_3)$; (**I**) $((WR)_3(DipR)_2)$; (**J**) $((WR)_4DipR))$ were conducted using 100 µM peptide concentration.

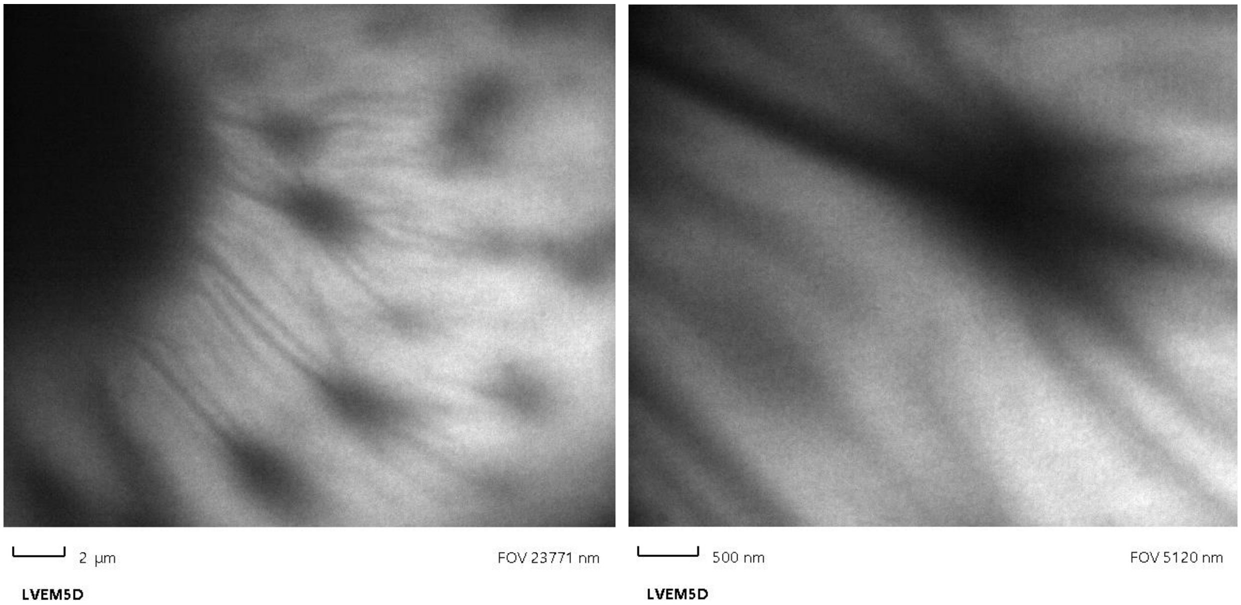

Figure S14. Transmission electron microscopy image of [(DipR)$_2$(WR)$_3$].

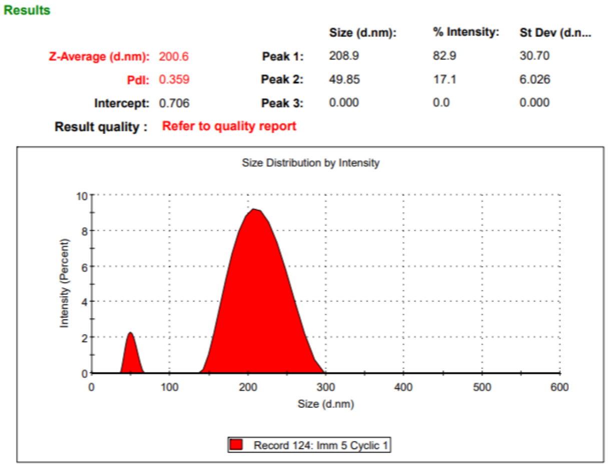

Figure S15. Dynamic light scattering of [DipR]$_5$ (10.0 µM), Z Average 200.6, PI 0.359.

Videos of Cellular Uptake

1. Multiple Images of Cells for Only Phosphopeptide in the Absence of $[DipR]_5$ (MDA-MB-231 Cells) video: https://photos.app.goo.gl/WbnNz5n4g932rp9G8
2. Multiple Images of Cells for Phosphopeptide and $[DipR]_5$ (10 μM) Physical Mixture (MDA-MB-231 Cells) video: https://photos.app.goo.gl/71cfQ5RHVMQyTQo26
3. 3D Video of Cells for Only Phosphopeptide in the Absence of $[DipR]_5$ (MDA-MB-231 Cells) video: https://photos.app.goo.gl/eLEcPGdybmbNK8pZ7
4. 3D Video of Cells for Phosphopeptide and $[DipR]_5$ (10 μM) Physical Mixture (MDA-MB-231 Cells) video: https://photos.app.goo.gl/r4gHanTkgBVKQr8i6
5. Multiple Images of Cells for No Treatment in the Absence of F′-[K(DipR)$_5$] (MDA-MB-231 Cells) video: https://photos.app.goo.gl/jafzMa6sLw1yMcUTA
6. Multiple Images of Cells for F′-[K(DipR)$_5$] (10 μM) Conjugate (MDA-MB-231 Cells) video: https://photos.app.goo.gl/nB4zksjpUddazbUo6

   3D Video of Cells for No Treatment in the Absence of F′-[K(DipR)$_5$] (MDA-MB-231 Cells) video: https://photos.app.goo.gl/rEw9K8wuJ4cj41q97
7. 3D Video of Cells for F′-[K(DipR)$_5$] (10 μM) Conjugate (MDA-MB-231 Cells) video: https://photos.app.goo.gl/5y9EFUC9uarrqcMy5
8. All the videos: https://photos.app.goo.gl/axHW8J5VvuKKzQ537

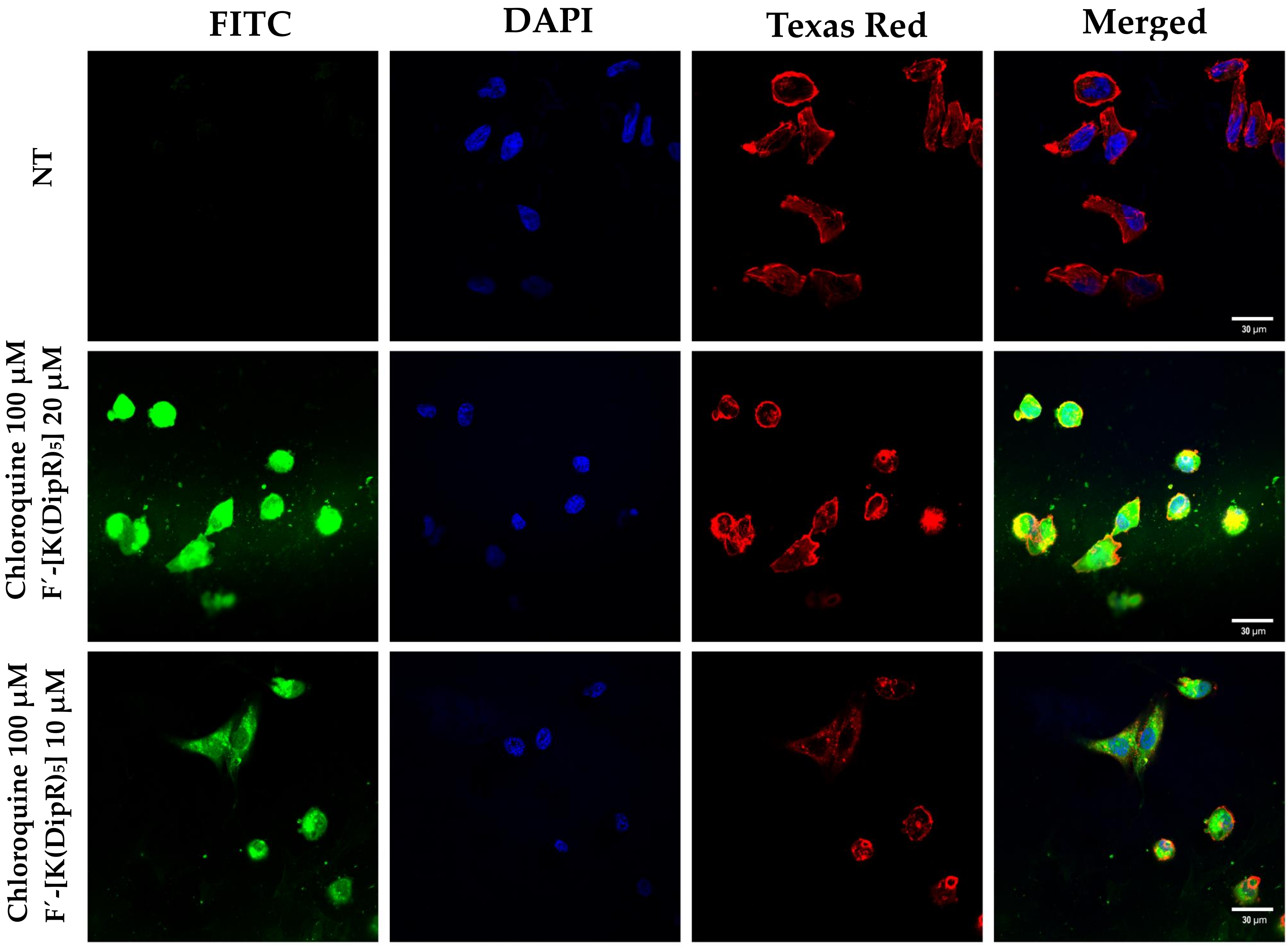
FITC
DAPI
Texas Red
Merged
NT
Chloroquine 100 µM
F´-[K(DipR)5] 20 µM
Chloroquine 100 µM
F´-[K(DipR)5] 10 µM
30 µm
30 µm
30 µm

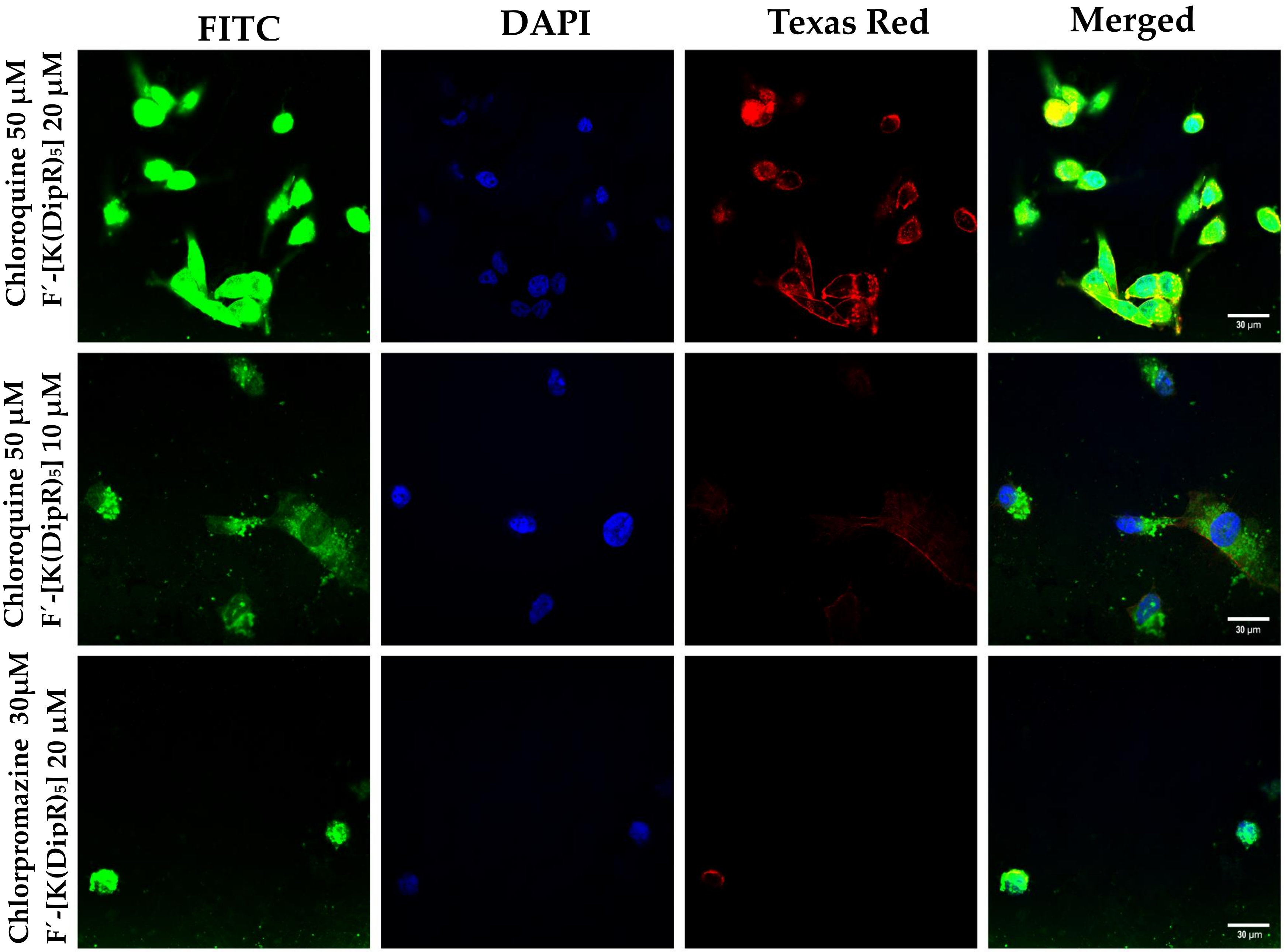
FITC
DAPI
Texas Red
Merged
Chloroquine 50 µM
F′-[K(DipR)5] 20 µM
Chloroquine 50 µM
F′-[K(DipR)5] 10 µM
Chlorpromazine 30µM
F′-[K(DipR)5] 20 µM
30 µm
30 µm
30 µm

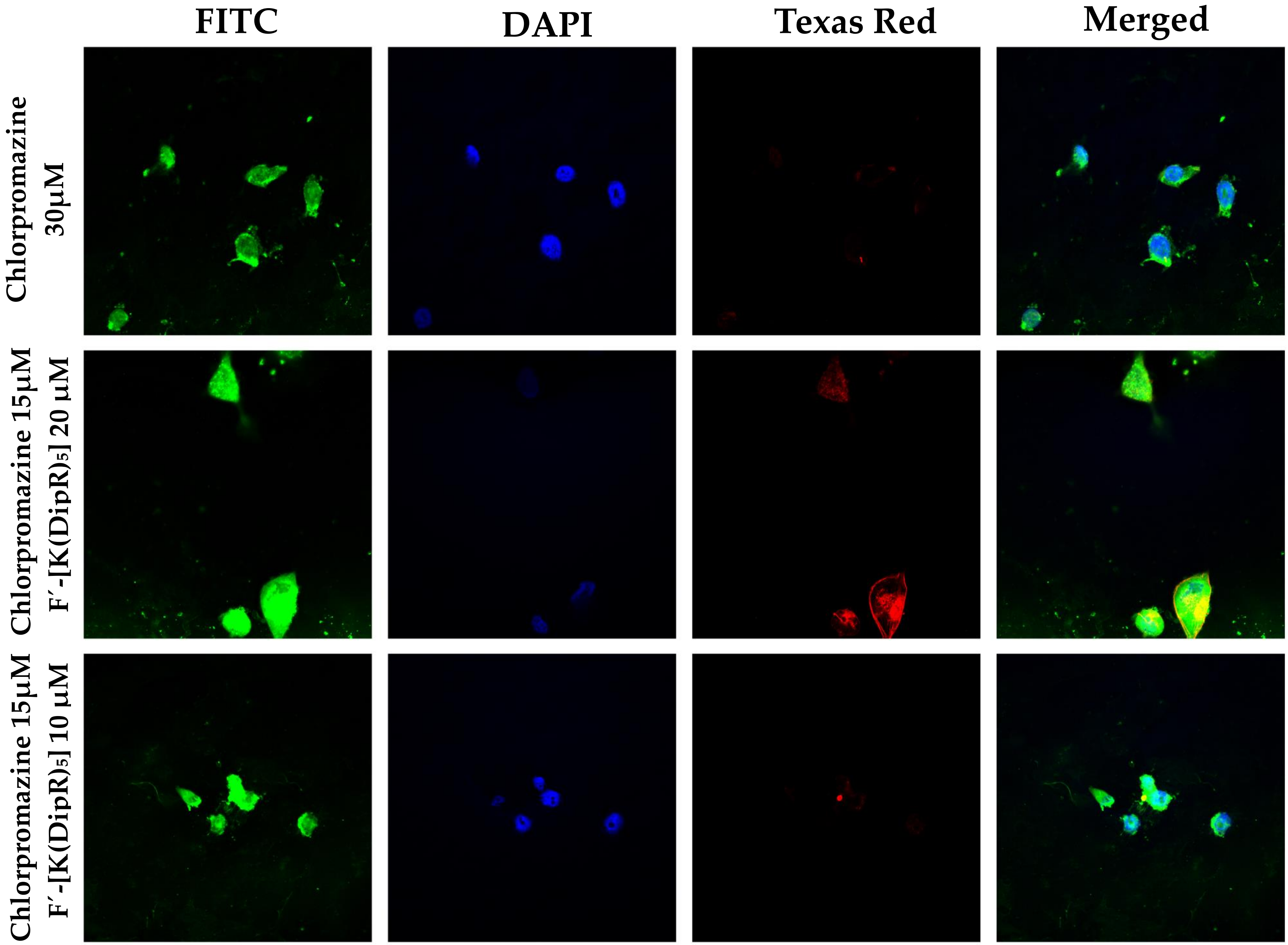

Figure S16. Confocal microscopy images of F´-[K(DipR)$_5$] (10 and 20 μM), chloroquine (100 and 50 μM), and chlorpromazine (30 and 15 μM), in MDA-MB-231 cells after 3 h incubation. NT = No Treatment.

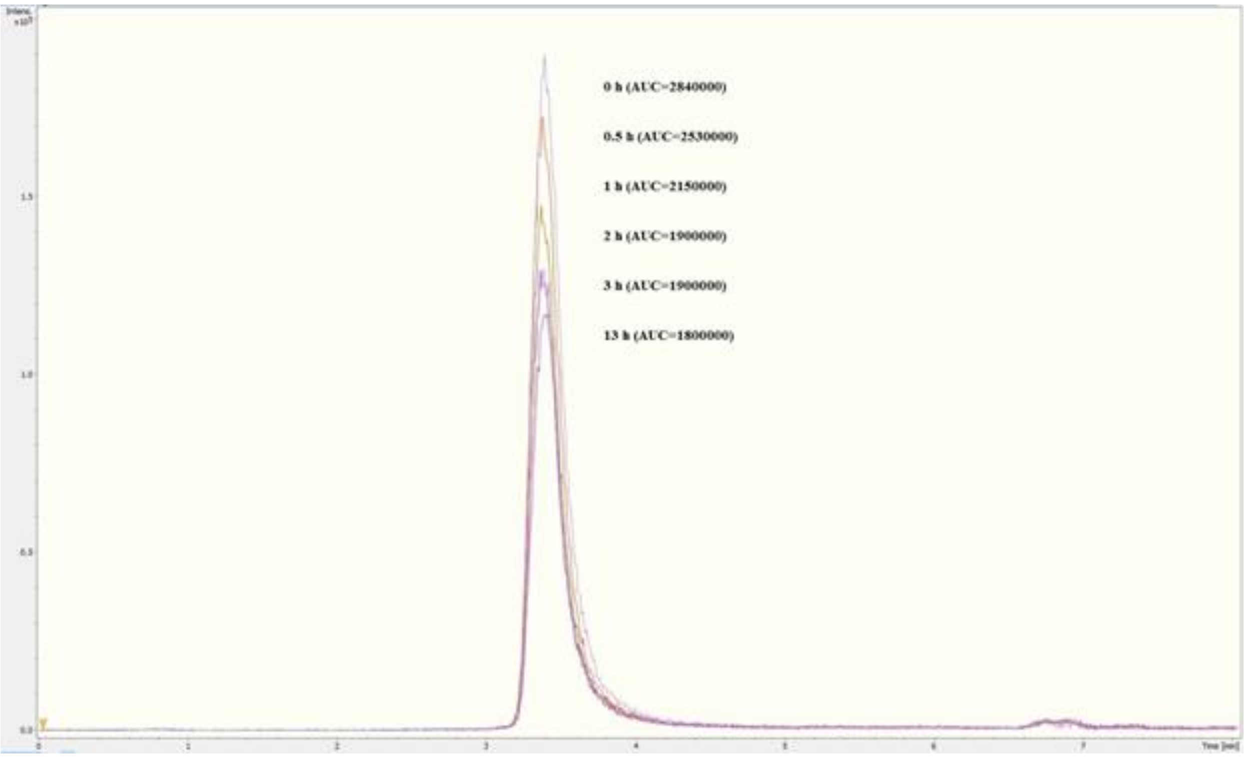

Figure S17. The plasma stability of $[DipR]_5$ chromatogram.

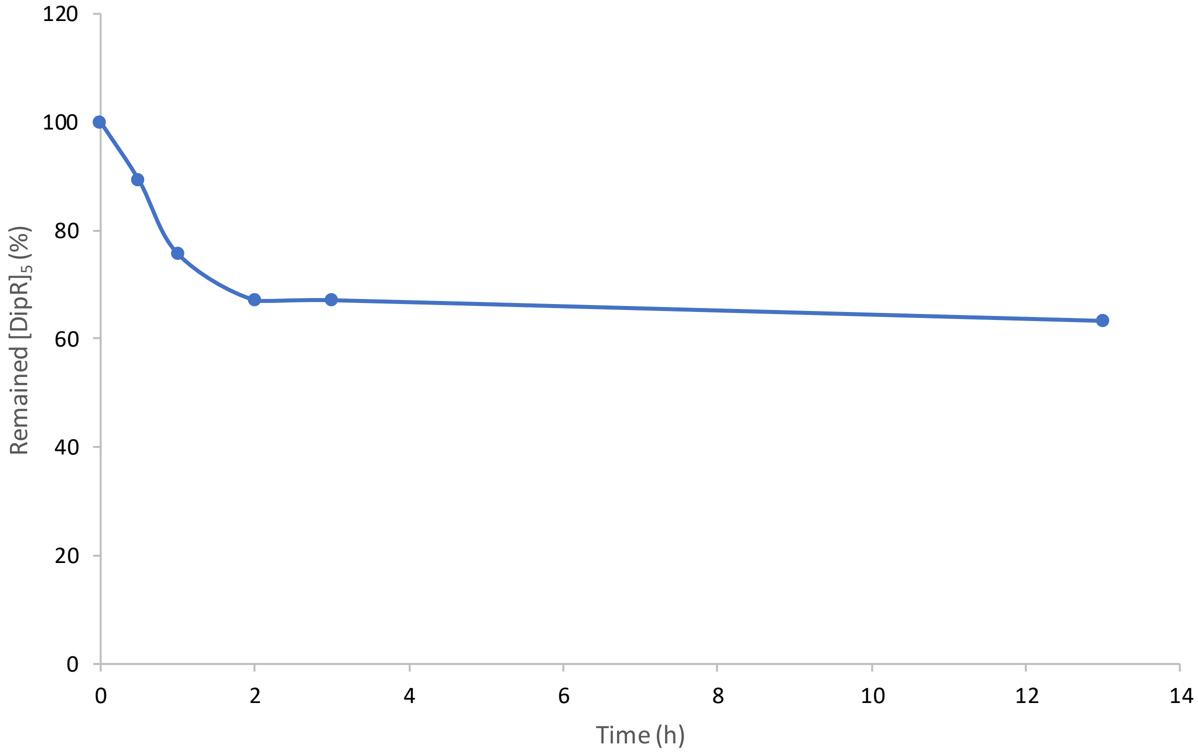

Figure S18. The plasma stability of $[DipR]_5$.

Table S1. Estimate of peptide secondary structures.[a.]

Peptide	α-helix	β-sheet	Turn	Other
$[DipR]_5$	21.4%	12.1%	16.6%	49.9%
$R(DipR)_4$	6.0%	36.5%	17.7%	39.8%
$((WR)_4(DipR))$	23.4%	26.9%	15.2%	34.5%
$[(DipR)(WR)_4]$	28.9%	16.8%	13.8%	40.5%
$((WR)_3(DipR)_2)$	21.1%	29.5%	14.8%	34.6%
$[(DipR)_2(WR)_3]$	20.8%	18.6%	12.8%	47.8%
$((WR)_2(DipR)_3)$	8.5%	38.0%	17.1%	36.4%
$[(DipR)_3(WR)_2]$	13.1%	31.5%	17.0%	38.4%
$((WR)(DipR)_4)$	29.8%	18.6%	15.6%	36.0%
$[(DipR)_4(WR)]$	8.6%	34.9%	16.8%	39.7%

[a]calculated using principal component regression (PCR) analysis of CD Spectra (Spectra Manager Version 2, CD Multivariate SSE, Jasco Corporation).
